# On the In Silico Evaluation of the Emission Quantum Yield of Cyclometalated Ir(III) Complexes

**DOI:** 10.1021/acs.inorgchem.5c00700

**Published:** 2025-04-16

**Authors:** Iván Soriano-Díaz, Alicia Omist, Ilya D. Dergachev, Sergey A. Varganov, Enrique Ortí, Angelo Giussani

**Affiliations:** 1 Institute for Molecular Science (ICMol), 16781 Universitat de València, Catedrático José Beltrán 2, Paterna 46980, España; 2 Department of Chemistry, 6851 University of Nevada, Reno, 1664 N. Virginia Street, Reno, Nevada 89557-0216, United States

## Abstract

The in silico determination of the emission quantum yield (Φ_em_) of a cyclometalated Ir­(III) complex requires the evaluation of all its possible radiative and nonradiative decays. The task is challenging, particularly when more than one minimum is present on the potential energy surface of the emitting lowest-energy triplet T_1_, a situation more common than what was previously thought. In the present contribution, we study all possible radiative and nonradiative paths for the two cyclometalated Ir­(III) complexes, [Ir­(ppy)_2_(pyim)]^+^ and [Ir­(diFppy)_2_(dtb-bpy)]^+^, indeed characterized by T_1_ minima of both metal-to-ligand charge transfer and ligand-centered nature. Comparing the computed emission quantum yields accounting for all processes and for only those processes in principle more relevant, we aimed at judging the importance of characterizing all decays to provide an accurate estimation of Φ_em_. Evaluating the barrier between emitting T_1_ and nonradiative ^3^MC states obtaining the corresponding transition state or approximating the latter using the CI-NEB method, we aimed at judging the importance of performing transition state optimizations. The latter task, due to its intrinsic complexity and poor convergence behavior, is a bottleneck for the characterization of the photophysics of a complex and consequently prevents a more efficient screening of cyclometalated Ir­(III) complexes for technological applications.

## Graphical abstract

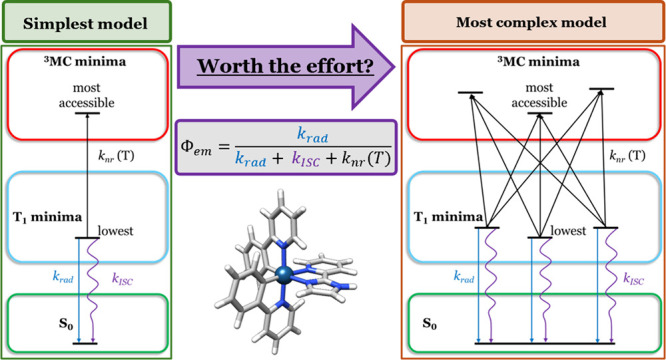

## Introduction

Ionic transition-metal complexes (iTMCs) have risen in popularity due to their promising electrochemical and photophysical properties in different fields such as light-emitting electrochemical cells (LECs),^1^ photocatalysis hydrogen production,^2^ biological imaging,^3,4^ luminescence sensitizers,^5^ chemosensors,^6–8^ and photodynamic therapy.^9–11^ As solid-state lighting devices, LECs present some advantages compared to organic light-emitting diodes (OLEDs) owing to their less rigid encapsulation leading to lower-cost simpler structures.^1^ The main feature that iTMCs must display for their use in LECs, and also in other applications, is a high-emission quantum yield (Φ_em_). To understand the differences in the emission properties and to conduct a chemical design of iTMCs with adjusted Φ_em_, a comprehensive theoretical investigation is essential to elucidate the radiative and nonradiative pathways (characterized by the respective *k*_rad_ and *k*_nr_ rate constants) contributing to their photophysical behavior and Φ_em_. Among iTMCs, cationic heteroleptic cyclometalated Ir­(III) complexes with the general formula [Ir­(C^N)_2_(N^N)]^+^ have gained significant attention due to their appealing photophysical properties, such as high Φ_em_ values, long-lived excited states, and strong spin–orbit coupling.

In heteroleptic [Ir­(C^N)_2_(N^N)]^+^ complexes, emission comes as phosphorescence from the lowest-energy triplet state T_1_, which normally presents a mixed metal-to-ligand/ligand-to-ligand charge transfer nature (^3^MLCT/^3^LLCT, hereafter the ^3^MLCT state).^12–14^ This state is usually characterized by a HOMO→LUMO transition, where the HOMO is composed by the *d* orbitals of the Ir center and the π orbitals of the C^N ligand, and the LUMO relies almost exclusively on the π* orbital of the N^N ligand. Previously, it was assumed that the involvement of the metal in the emitting state was essential to promote the T_1_-to-S_0_ spin-forbidden transition. However, recent works^13,14^ have shown that triplet ligand-centered (^3^LC) excited states can display phosphorescence lifetimes of the same order of magnitude as ^3^MLCT states and, therefore, can also play a fundamental role in the emission process. Owing to the two distinct ligands present in [Ir­(C^N)_2_(N^N)]^+^ complexes, two types of ^3^LC states arise, those localized on the C^N ligands (consequently resulting in two ^3^LC_C^N_ states) and one localized on the N^N ligand (^3^LC_N^N_). If ^3^MLCT and ^3^LC states are present in the T_1_ potential energy surface (PES), their relative relevance has to be considered to evaluate the global emission properties.

Regarding the intramolecular nonradiative decay path, it is normally accepted that it can be broken down into two main processes. The first process involves direct intersystem crossing (ISC) between the emitting triplet state (T_1_) and the ground state (S_0_) at the T_1_ minima.^15,16^ The corresponding rate constant is indicated as *k*_*ISC*_. This phenomenon is allowed due to the strong spin–orbit coupling (SOC) present in the complexes. The second process relies on the population of triplet metal-centered (^3^MC) states. These ^3^MC states are basically characterized by a t_2g_ → e_g_* transition, as the Ir­(III) complexes are d^6^ with a strong ligand field splitting, resulting in low-spin complexes. The energy minima of the ^3^MC states are characterized by a significant geometrical distortion compared with the S_0_ minimum, which produces a huge destabilization of the S_0_ state. This in turn determines the presence of accessible singlet–triplet crossing regions (STCs) between the ^3^MC and S_0_ states, which allow for an efficient nonradiative decay. As the ^3^MC and T_1_ states belong to the same PES, there must be a transition state (TS) connecting them, which in turn determines the presence of an energy barrier for activating the T_1_→^3^MC process. The rate constant for this process is then denoted *k*_nr_(*T*), so as to remark its dependence on the temperature due to the presence of the mentioned energy barrier.

According to previous work from our group and others,^17–21^ there are two distinct kinds of ^3^MC states. On the one hand, the so-called symmetric or asymmetric axial ^3^MC states (hereafter ^3^MC_ax_) are characterized by the elongation of one or two Ir—N_C^N_ bonds. On the other hand, the so-called equatorial ^3^MC states (hereafter ^3^MC_eq_) imply the elongation of one Ir—N_N^N_ bond and the posterior rotation of the ring involved. This determines that in ^3^MC_eq_ minima, the coordination number of Ir is decreased from 6 to 5 to get a distorted bipyramidal trigonal conformation.

To provide an accurate estimation of Φ_em_ is essential to consider both the radiative and nonradiative decay paths, which can be characterized at different levels of modeling. In this contribution, we analyze how different approaches affect the computed value of Φ_em_ by studying the role of the number of decay paths considered in the calculation and the way the associated barriers are evaluated. We performed such an analysis by theoretically investigating the photophysics of two green-emitter Ir­(III) complexes: [Ir­(ppy)_2_(pyim)]^+^ and [Ir­(diFppy)_2_(dtb-bpy)]^+^ (complexes **1** and **2**, respectively, see Figure 1). Here, ppy denotes 2-phenylpyridine and pyim denotes 2-(1H-imidazol-2-yl)­pyridine, while diFppy refers to 2-(2,4-difluorophenyl)­pyridine and dtb-bpy is 4,4′-di*tert*-butyl-2,2′-bipyridine. These two complexes were selected since their emission quantum yields are experimentally known (Φ_em_ = 20.40 and 71.00% for complex **1** and **2** in acetonitrile, respectively).^22,23^ In addition, according to previously published studies,^12,14,22,23^ they are both characterized by different T_1_ minima, and so they provide a testing case of the importance of accounting for all the present emitting minima. Finally, as green emitters, the *k*_*ISC*_ rate constant is expected to be less relevant than the *k*_nr_(*T*), which in turn is important for the reliability of the computed Φ_em._ In fact, due to the inaccurate description provided by the harmonic approximation at the basis of the formula used to estimate *k*_*ISC*_, the computation of the latter is associated with higher errors, which determines that when the T_1_-to-S_0_ ISC process is expected to be the most relevant (as in red-emitting complexes) the accuracy of the Φ_em_ can be questioned.^16,24^

**Figure 1:**
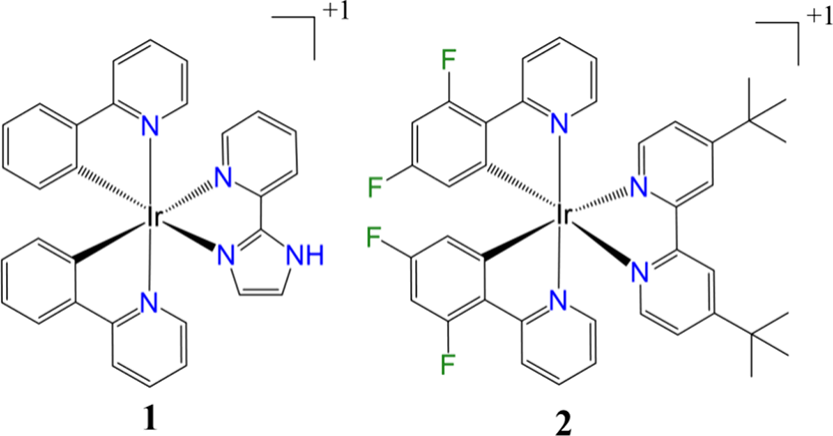
Chemical structure of complexes **1** ([Ir­(ppy)_2_(pyim)]^+^) and **2** ([Ir­(diFppy)_2_(dtb-bpy)]^+^).

## Computational Details

Unless otherwise stated, all electronic structure calculations were done with the ORCA software^25^ (version 5.0.4). All the triplet and singlet states were optimized at the DFT level of theory, without imposing any symmetry constraint, using PBE0 ^26^ as an exchange-correlation hybrid functional. The def2-SVP basis set was selected for all the atoms.^26–28^ The inner electrons of the Ir center were substituted by the Stuttgart–Dresden effective core potential while explicitly treating the outer core [(5s)^2^(5p)^6^] and the valence (5d)^6^ electrons.^27^ This strategy was previously employed by our group for similar systems.^14,17^ For all the calculations implying triplet states, the unrestricted DFT approximation was employed (UDFT), checking that the spin contaminations were always between 1.95 and 2.05 to guarantee they were true triplets states.

To characterize the minimum energy path (MEP) connecting two states and to obtain a good and reliable starting structure to get the transition state (TS) between the corresponding energy minima, the climbing-image nudged-elastic band (CI-NEB)^28^ method was employed. The optimization of the minimum energy crossing points (MECPs) between singlet and triplet states was performed using the optimized geometry of the corresponding ^3^MC minimum as a starting point. Solvent effects were considered in all the calculations using the conductor-like polarizable continuum model (CPCM)^29^ and acetonitrile as solvent. Frequency calculations were conducted to check the nature of all the critical points. All the TSs were further characterized employing the intrinsic reaction coordinate (IRC)^30^ approach to guarantee they connected the desired triplet minima.

Time-dependent DFT (TD-DFT) calculations were performed to check the nature of the optimized triplet minima, alongside a natural transition orbital (NTO)^31^ analysis using the TheoDORE software.^32^ Such an analysis is important for the here-studied two complexes, since up to three emitting T_1_ minima of different nature can, in principle, be located.

To calculate the radiative and intersystem crossing rate constants (*k*_rad_ and *k*_ISC_, respectively) from the emitting triplet state to the ground state, additional TD-DFT calculations, including spin–orbit coupling (TD-DFT SOC), were carried out using the ZORA Hamiltonian^33^ to simulate the relativistic effects. A mean-field spin–orbit operator^34^ was selected to compute the first 25 singlet and triplet excited states. In these calculations, the basis set employed was ZORA-def2-SVP^35^ for non-Ir atoms and ZORA-def2-TZVP^36^ for the Ir center. The TD-DFT SOC calculations provide the transition dipole moment between each triplet substate and the singlet ground state that, together with the energies afforded by the same calculation, allows to estimate the radiative rate constant for each triplet substate using the following equation.^37,38^

$$k_{\mathrm{rad}}(A,X)=\frac{4\alpha }{3e^{2}c^{2}\hbar^{3}}(E_{A}-E_{X}{)}^{3}|\mu_{\mathrm{el}}(A,X){|}^{2}$$ 1

In eq 1, *A* indicates the substate from which the radiation is produced, *X* is the ground state, α is the fine-structure constant, *e* is the electron charge, *c* is the speed of light, ℏ is the reduced Planck constant, and μ_el_ is the electric transition dipole moment. Once the radiative rate constants are obtained for the three triplet substates, it is possible to get the total radiative rate constant (*k*_rad_) for a given temperature by considering their different thermal population using the Boltzmann distribution^39^ and employing the following equation.

$$k_{\mathrm{rad}}(T_{1})=\frac{k_{1}+k_{2}e^{-E_{1,2}/k_{B}T}+k_{3}e^{-E_{1,3}/k_{B}T}}{1+e^{-E_{1,2}/k_{B}T}+e^{-E_{1,3}/k_{B}T}}$$ 2

where *k*_1_, *k*_2_, and *k*_3_ are the radiative rates of the three triplet substates in which the emitting T_1_ state splits, *E*_1,2_ and *E*_1,3_ are the energy differences between sublevels 2 and 1 and 3 and 1, respectively, *k*_*B*_ is the Boltzmann constant, and *T* is the temperature (298.15 K).

To compute the *k*_ISC_ rate constant, the Condon approximation, within the Fermi’s Golden rule,^16,40^ was employed (eq 3).

$$k_{\mathrm{ISC}}(T_{1})=\frac{2\pi }{\hbar }|\langle \psi_{T_{1}}|{Ĥ}_{\mathrm{SO}}|\psi_{S_{0}}\rangle {|}^{2}(E_{T_{1}}-E_{S_{0}})$$ 3

In this expression, ℏ is the reduced Planck constant, *Ĥ*_SO_ is the spin–orbit operator, and *E*_*T*_1__ – *E*_*S*_0__ represents the vertical energy difference between the triplet and singlet states at the T_1_ minimum-energy geometry.

To compute the *k*_nr_(*T*) rate constant, we need in turn to compute the rate constant *k*_*x*_ of the process leading from one minimum to another minimum (both belonging to the same PES), overcoming the energy barrier *E*_*x*_. Such a rate constant is here computed using the NAST software.^41–43^ The latter employs an Arrhenius-like equation, within the classical transition state theory (TST),^44,45^ that utilizes the partition functions of the TS and reactant species to account for the electronic, vibrational, and rotational levels, and includes the zero-point energy (see Section S1 of the Supporting Information). The *k*_*x*_ rate constant is then evaluated using eq 4.

$$k_{x}=\kappa \frac{k_{B}T}{h}\left(\frac{Q_{\mathrm{TS}}}{Q_{R}}\right)e^{-E_{x}/k_{B}T}$$ 4

where $\kappa \frac{k_{b}T}{h}$ is the preexponential factor, κ is equal to 1 within the classical TST, *k*_*B*_ is the Boltzmann constant, *T* is the temperature (298.15 K), *h* is the Planck constant, *E*_*x*_ is the zero-point-corrected activation energy through the TS, and $\left(\frac{Q_{\mathrm{TS}}}{Q_{R}}\right)$ is the ratio between the partition functions of the TS (*Q*_TS_) and the reactant (*Q*_*R*_).

## Results and Discussion

A full theoretical characterization of the photophysics of complexes **1** and **2** was initially performed, the results of which are summarized in section S2 of the Supporting Information. As expected, we indeed found two types of emitting T_1_ minima in the two complexes, of ^3^MLCT and ^3^LC_C^N_ nature, respectively, which will then allow us to study the effect of modeling all of them or just the lowest one when estimating the value of Φ_em_.

After such a characterization, we proceeded to study the impact that the number of paths considered and the way they are described have on the evaluation of Φ_em_. As presented in Figure 2, we have adopted four models (**a**, **b**, **c**, and **d**) that differ on the number of T_1_ and ^3^MC minima included. For each T_1_ minimum, the corresponding radiative and nonradiative decay paths, the latter depending on the number of ^3^MC minima, are investigated. For each ^3^MC minimum, its connection to all the included T_1_ minima is considered. In this framework, the simplest model **a** includes only the lowest-energy T_1_ minimum and the most accessible ^3^MC state. Meanwhile, the most complex model **d** includes all T_1_ and ^3^MC minima and their corresponding radiative and nonradiative decay pathways. We also analyzed the intermediate cases either using only the lowest energy T_1_ state and all the ^3^MC states, model **b**, or using all the T_1_ states but only the most accessible ^3^MC state, model **c**. It is to note that, within this approach, the lowest energy ^3^MC state is not always the most accessible ^3^MC state, as for instance happens for complex **2**.

**Figure 2:**
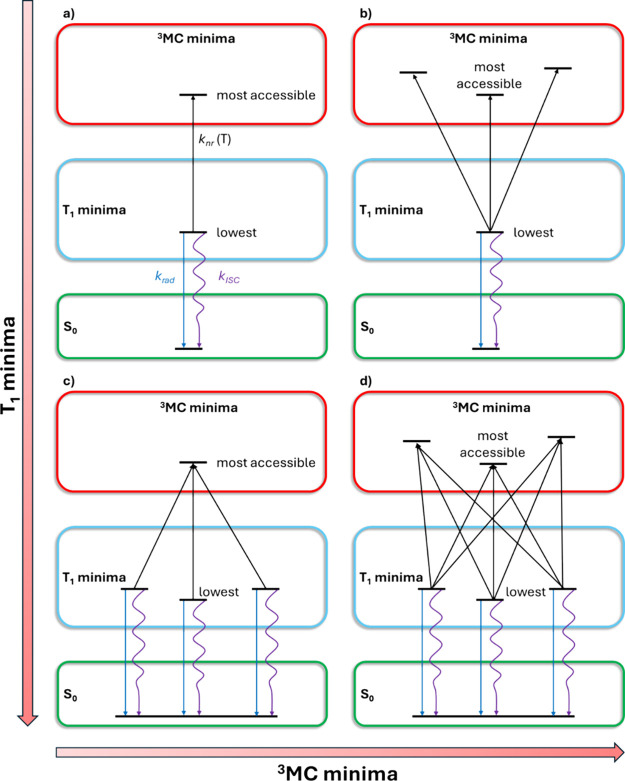
Schematic diagram representing the four models (**a**, **b**, **c**, and **d**) adopted to evaluate the photophysics of complexes **1** and **2**. The vertical axis represents the number of T_1_ minima considered while the horizontal axis represents the number of ^3^MC states included.

In the case that more than one T_1_ minimum play a role (**c** and **d** approaches, Figure 2), it is assumed that all T_1_ minima are accessible after the excitation process. In this sense, the Boltzmann distribution was employed to determine their relative population, and average Boltzmann values were computed for the *k*_rad_, *k*_ISC_, and *k*_nr_(*T*) rate constants. eq 2 was then used, where now *k*_1_, *k*_2_, and *k*_3_ would be the *k*_rad_, *k*_ISC_, or *k*_nr_(*T*) rate constants calculated for the different T_1_ states, and Δ*E* would be the energy difference between the T_1_ minima.

Each ^3^MC-mediated nonradiative decay path is computed using the kinetic model represented in Figure 3,^24,46^ which involves the T_1_ and ^3^MC minima and a ^3^MC/S_0_ MECP. The first step relies on the population of the ^3^MC state from the T_1_ minimum. Once the ^3^MC state is populated, two different processes are possible: either to repopulate the T_1_ minimum or to reach an MECP region. The energy barriers (rate constants) for each step are referred to as *E*_*a*_ (*k*_*a*_), *E*_*b*_ (*k*_*b*_), and *E*_*c*_ (*k*_*c*_), respectively. The *E*_*a*_ and *E*_*b*_ barriers are obtained through the computation of the TS separating the T_1_ and ^3^MC minima. The *E*_*c*_ barrier is estimated from the characterization of the nearby ^3^MC/S_0_ MECP. A possible simplification is the evaluation of the *E*_*a*_/*E*_*b*_ barriers through the computation of the corresponding CI-NEB instead of the actual TS. CI-NEBs are in fact computationally easier to obtain and in general constitute a good estimation of the corresponding TS.^28^ Independently on how the *E*_*a*_/*E*_*b*_ barriers are evaluated, once the three rate constants are calculated (using in each case eq 4), the *k*_nr_(*T*) nonradiative rate constant is computed using eq 5.

$$k_{\mathrm{nr}}=\frac{k_{a}k_{c}}{k_{b}+k_{c}}$$ 5

**Figure 3:**
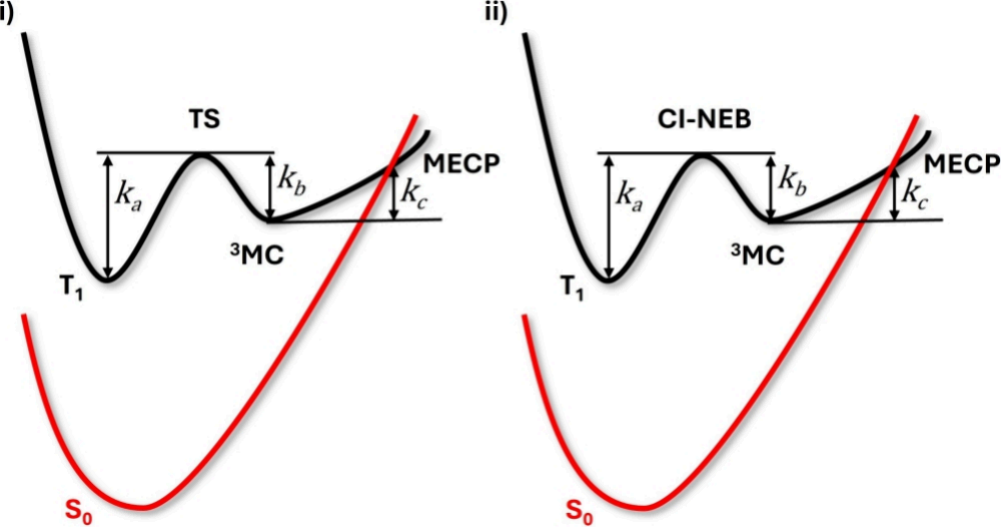
Kinetic models used to compute the nonradiative decay rate constant through a ^3^MC state. The *E*_a_/*E*_b_ barrier can be evaluated either computing the TS separating the T_1_ and ^3^MC minima (i) or approximating it using the corresponding CI-NEB (ii). *k*_*a*_, *k*_*b*_, and *k*_*c*_ represent the rate constants for each step.

Strictly speaking, the *k*_*c*_ value is also determined by the strength of the coupling between the ^3^MC and S_0_ states, but since these states are expected to have a very high SOC, the value of *k*_*c*_ is computed evaluating only the corresponding energy barrier (*E*_*c*_).

Of the three rate constants that determine the emission quantum yield, i.e., *k*_rad_, *k*_ISC_, and *k*_nr_(*T*), the number of T_1_ minima included directly affects the *k*_rad_ and *k*_ISC_ values, while both the number of T_1_ minima and ^3^MC states have a relevant effect on *k*_nr_(*T*). Once these three rate constants are computed, the emission quantum yield can be calculated using eq 6.

$$\Phi_{\mathrm{em}}(\%)=\frac{k_{\mathrm{rad}}}{k_{\mathrm{rad}}+k_{\mathrm{ISC}}+k_{\mathrm{nr}}(T)}\times 100$$ 6

In the following section, we first analyze how the models proposed influence the values of *k*_rad_ and *k*_ISC_. Then, we study the effect on *k*_nr_(*T*), and finally on the Φ_em_ calculated for complexes **1** and **2**.

### Radiative (*k*_rad_) and Intersystem Crossing (*k*_ISC_) Rate Constants

According to the models proposed (Figure 2), the evaluation of *k*_rad_ and *k*_ISC_ is affected only by the number of T_1_ minima included in the calculation. Thus, models **a** and **b**, on one hand, and models **c** and **d**, on the other hand, will lead to the same *k*_rad_ and *k*_ISC_ values. We then only focus on models **a** and **c**, representing the case of incorporating only the lowest and all the T_1_ minima, respectively.

After geometry optimization (see Section S2 in the Supporting Information), three distinct T_1_ minima were characterized for complex **1**, one of ^3^MLCT nature and the other two of ^3^LC_ppy_ character (hereafter ^3^MLCT, ^3^LC_ppy 1_, and ^3^LC_ppy 2_, respectively). The three minima are almost degenerate with differences within 0.01 eV, the ^3^LC_ppy 2_ state being the lowest-energy one (Table 1). The ^3^LC_ppy 2_ minimum is consequently the only one included in model **a**, whereas the three minima are accounted for in model **c**. For complex **2**, two distinct T_1_ minima of ^3^MLCT and ^3^LC_ppy_ nature (hereafter ^3^MLCT and ^3^LC_ppy 1_) were characterized. A second ^3^LC_ppy_ state is present, whose minimum, due to the symmetry of the complex, is actually equivalent to the ^3^LC_ppy 1_ structure. In this case, the adiabatic energy difference is higher (0.07 eV) with the ^3^MLCT state as the lowest T_1_ minimum (Table 1). The ^3^MLCT minimum is consequently the only one included in model **a**, whereas, in model **c**, the two equivalent ^3^LC_ppy_ structures are present in addition to the ^3^MLCT minimum.

**Table 1:** DFT PBE0/def2-SVP­(CH_3_CN) Relative Energies (in eV) Computed for the T_1_ state of Complexes **1** and **2** at the Optimized T_1_, ^3^MC, and MECP Geometries^a^

**complex**	**geometry**	**T**_**1**_
**1**	(^3^MLCT)_min_	0.00
	(^3^LC_ppy 1_)_min_	0.01
	(^3^LC_ppy 2_)_min_	0.00
	(^3^MC_ax 1_)_min_	0.46
	(^3^MC_ax 1_/S_0_)_stc‑mecp_	0.50
	(^3^MC_ax 2_)_min_	**0.45**
	(^3^MC_ax 2_/S_0_)_stc‑mecp_	0.47
	(^3^MC_eq 1_)_min_	0.53
	(^3^MC_eq 1_/S_0_)_stc‑mecp_	0.55
	(^3^MC_eq 2_)_min_	**0.34**
	(^3^MC_eq 2_/S_0_)_stc‑mecp_	0.36
**2**	(^3^MLCT)_min_	0.00
	(^3^LC_ppy 1_)_min_	0.07
	(^3^MC_ax 1_)_min_	**0.45**
	(^3^MC_ax 1_/S_0_)_stc‑mecp_	**0.52**
	(^3^MC_eq 1_)_min_	**0.51**
	(^3^MC_eq 1_/S_0_)_stc‑mecp_	**0.53**

a All the reported energies are referred with respect to the T_1_ energy computed for the lowest T_1_ minimum of the corresponding complex. Key values discussed in the main text are reported in bold.

The emission energies (*E*_em_) from the obtained T_1_ minima (computed as the vertical energy difference between the triplet and the singlet ground state calculated at the UDFT and DFT level of theory, respectively) agree with the experimental emission energies, then supporting their involvement in the emission process of the two complexes (see Table S4).

For complex **1**, the value of *k*_rad_ using model **a** is 9.96 × 10^4^ s^–1^, which leads to a lifetime (τ) of 10.04 μs (Table 2). These values are relatively far from the experimental data of 6.50 × 10^5^ s^–1^ and 1.54 μs for *k*_rad_ and τ, respectively.^22^ On the other hand, when the three T_1_ minima are considered (model **c**), the values obtained (3.98 × 10^5^ s^–1^ and 2.52 μs for *k*_rad_ and τ, respectively) are in better agreement with the experimental data. It is interesting to note that the experimental *k*_*rad*_ value is higher than the rate constants from the ^3^LC_ppy 1_ and ^3^LC_ppy 2_ minima but lower than that from the ^3^MLCT minimum (see Table S6), thus suggesting that emission comes from the three minima instead of one. These results indicate that the inclusion of the three T_1_ minima is needed to account for the emission properties of complex **1**.

**Table 2:** Radiative Rate Constants (*k*_rad_), Lifetimes (τ), and Intersystem-Crossing Rate Constants (*k*_ISC_) Computed for Complexes **1** and **2** Accordingly to the Four Adopted Models **a**–**d** (The Corresponding Experimental Values Are Also Included)

**complex**	**model**	**τ (μs)**	*k*_rad_ **(s^–1^)**	*k*_ISC_ **(s^–1^)**
**1**	**a/b**	10.04	9.96 × 10^4^	1.28 × 10^3^
	**c/d**	2.52	3.98 × 10^5^	1.80 × 10^4^
	experimental^a^	1.54	6.50 × 10^5^	
**2**	**a/b**	2.38	4.20 × 10^5^	2.22 × 10^4^
	**c/d**	2.28	4.04 × 10^5^	3.12 × 10^4^
	experimental^b^	1.76	5.68 × 10^5^	

a Experimental data from ref 22.

b Experimental data from ref 23.

For complex **2**, the calculation of *k*_*rad*_ with only the lowest T_1_ minima (^3^MLCT) provides a value of 4.20 × 10^5^ s^–1^ (Table 2) in good agreement with the experimental value of 5.68 × 10^5^ s^–1^.^23^ The Boltzmann averaged result from model **c** gives almost the same estimated value (4.04 × 10^5^ s^–1^) since, at the temperature considered (298.15 K), the energy difference between the T_1_ minima (0.07 eV) determines that nearly 90% of the population remains in the ^3^MLCT state. Moreover, the *k*_rad_ value computed for the ^3^LC_ppy 1_ minimum is of the same order of magnitude as the one characterizing the ^3^MLCT minimum-energy structure (see Table S6). Then, for complex **2**, the simplified model **a** already provides a reasonable value for *k*_rad_.

Experimentally, the *k*_ISC_ value cannot be measured because only the total nonradiative decay constant can be recorded. For complex **1**, models **a** and **c** lead to *k*_ISC_ values equal to 1.28 × 10^3^ and 1.80 × 10^4^ s^–1^, respectively (Table 2). As the *k*_ISC_ values for ^3^MLCT and ^3^LC states differ in 1 order of magnitude (Table S6) and these states are almost degenerate (Table 1), employing only the lowest ^3^LC_ppy 2_ minimum leads to a significantly smaller rate constant (1.28 × 10^3^ s^–1^). For complex **2**, both approaches provide similar results with *k*_ISC_ values 2.22 × 10^4^ and 3.12 × 10^4^ s^–1^ from models **a** and **c**, respectively. As for the *k*_rad_ values, this can be associated with the fact that according to the adopted Boltzmann distribution, 90% of the population remains in the lowest state (^3^MLCT). Thus, although the *k*_ISC_ values for the ^3^MLCT and ^3^LC states differ in 1 order of magnitude (Table S6), the average *k*_ISC_ is dominated by the ^3^MLCT contribution.

### Temperature-Dependent Nonradiative *k*_nr_(*T*) Rate Constant

Regarding the evaluation of *k*_nr_(*T*), according to the models sketched in Figure 2, we first need to determine the most accessible ^3^MC state. For complex **1**, four ^3^MC minima (hereafter ^3^MC_ax 1_, ^3^MC_ax 2_, ^3^MC_eq 1_, and ^3^MC_eq 2_) were characterized, and the corresponding MECPs with the ground state were determined (hereafter (^3^MC_ax 1_/S_0_)_stc‑mecp_, (^3^MC_ax 2_/S_0_)_stc‑mecp_, (^3^MC_eq 1_/S_0_)_stc‑mecp_, and (^3^MC_eq 2_/S_0_)_stc‑mecp_) (see Section S2). A first way of judging the importance of each ^3^MC state is simply by looking at their relative energies. From the data collected in Table 1, the ^3^MC_eq 2_ route should be the most relevant since the corresponding minimum and MECP are lower in energy than all the other three ^3^MC minima by at least 0.11 eV. In fact, the energy of the ^3^MC_eq 2_ minimum_,_ relative to the lowest T_1_ minimum (^3^LC_ppy 2_), is equal to 0.34 eV, while the relative energy of the next ^3^MC minimum, i.e., the ^3^MC_ax 2_, is 0.45 eV. It is however fundamental to confirm the accessibility of each ^3^MC state through the evaluation of the energy barriers between the T_1_ and the^3^MC minima. This should be done, in principle, by computing the corresponding TS. As an upper bound of the true barrier, CI-NEBs can be computed in the first approximation. CI-NEBs are indeed much easier to obtain than TSs and constitute a good starting point for the localization of true TSs. Using the corresponding CI-NEB, the energy barrier leading to the ^3^MC_eq 2_ minimum is significantly lower (by 0.09 eV) than the adiabatic energy difference separating the T_1_ minima with all the other three ^3^MC states (see Tables 2 and 3). In fact, the highest *E*_*a*_ barrier to the ^3^MC_eq 2_ minimum from the three T_1_ minima is equal to 0.36 eV (Table 3), while the second lowest ^3^MC state, i.e., the ^3^MC_ax 2_ state, has an energy of 0.45 eV above the lowest T_1_ minimum (^3^LC_ppy 2_) (Table 1). This suggests that the ^3^MC_eq 2_ minimum is indeed the most relevant so that the inclusion of the other ^3^MC states will not significantly affect the values of the *k*_nr_(*T*) constant of complex **1** and then the theoretical estimation of the emission quantum yield. Moreover, the CI-NEB *E*_*a*_ barriers from the (^3^MLCT)_min_, (^3^LC_ppy 1_)_min_, and (^3^LC_ppy 2_)_min_ minima to the ^3^MC_eq 2_ (equal to 0.35, 0.36, and 0.36 eV, respectively, Table 3) are not more than 0.02 eV above the corresponding adiabatic energy differences (equal to 0.34, 0.35, and 0.34 eV, respectively, Table 1). Consequently, in principle, there would be no need to optimize the corresponding TSs since the *E*_*a*_ values obtained using TSs geometries can only be lower than the *E*_*a*_ values obtained with CI-NEBs and, of course, still larger than the corresponding adiabatic energy differences.

**Table 3:** Computed *E*_*a*_ Energy Barriers for All Possible ^3^MC-Mediated Nonradiative Decay Paths of **1** and **2**, using Either the Corresponding TS of CI-NEB Structures^a^

**complex**	**path**	*E*_*a*_ **(eV)**		
		**TS**	**CI-NEB**	**Δ** *E*_**adi**_
**1**	(^3^MLCT)_min_/(^3^MC_ax 1_)_min_	0.63	0.67	0.46
	(^3^MLCT)_min_/(^3^MC_ax 2_)_min_	0.61	0.63	0.45
	(^3^MLCT)_min_/(^3^MC_eq 1_)_min_	0.53	0.55	0.53
	(^3^MLCT)_min_/(^3^MC_eq 2_)_min_	**0.35**	**0.35**	0.34
	(^3^LC_ppy 1_)_min_/(^3^MC_ax 1_)_min_	0.63	0.68	0.45
	(^3^LC_ppy 1_)_min_/(^3^MC_ax 2_)_min_	0.60	0.64	0.44
	(^3^LC_ppy 1_)_min_/(^3^MC_eq 1_)_min_	**0.53**	0.54	0.52
	(^3^LC_ppy 1_)_min_/(^3^MC_eq 2_)_min_	**0.34**	**0.36**	0.33
	(^3^LC_ppy 2_)_min_/(^3^MC_ax 1_)_min_	0.64	0.66	0.46
	(^3^LC_ppy 2_)_min_/(^3^MC_ax 2_)_min_	0.60	0.62	0.45
	(^3^LC_ppy 2_)_min_/(^3^MC_eq 1_)_min_	0.54	0.56	0.53
	(^3^LC_ppy 2_)_min_/(^3^MC_eq 2_)_min_	**0.35**	**0.36**	0.34
**2**	(^3^MLCT)_min_/(^3^MC_ax 1_)_min_	**0.62**	**0.65**	**0.45**
	(^3^MLCT)_min_/(^3^MC_eq 1_)_min_		**0.56**	**0.51**
	(^3^LC_ppy 1_)_min_/(^3^MC_ax 1_)_min_			0.38
	(^3^LC_ppy 1_)_min_/(^3^MC_eq 1_)_min_		0.50	0.44

a The corresponding adiabatic energy difference, Δ*E*_adi_, is also reported. Key values discussed in the main text are marked in bold.

We however optimized all nonradiative T_1_ → ^3^MC decay paths through the computation of the corresponding TSs to prove the forecast results. The difference between the *E*_*a*_ barrier to the ^3^MC_eq 2_ minimum and the barriers leading to the others^3^MC minima is at least of 0.18 eV (Table 3). In fact, the higher *E*_*a*_ barrier involving the ^3^MC_eq 2_ minimum is equal to 0.35 eV, while the lowest *E*_*a*_ barrier involving any of the other three ^3^MC states is equal to 0.53 eV (associated with the (^3^LC_ppy 1_)_min_/(^3^MC_eq 1_)_min_ path). This in turn determines a difference of at least 3 orders of magnitude in the corresponding *k*_*a*_ rate constant (Table S7).

For each characterized nonradiative T_1_ → ^3^MC decay path, *E*_*b*_ is either larger or similar to the *E*_*c*_ value (Table S8), so that *k*_*b*_ is either smaller or of the same order of magnitude than *k*_*c*_ (Table S7). This in turn makes it so that the denominator of eq 5 can be approximated as equal to either *k*_*c*_ or 2*k*_*c*_, so that *k*_nr_ will be equal to either *k*_*a*_ or 0.5*k_*a*_. Consequently, the value of *k*_nr_ is mostly determined by *k*_*a*_, so the above-reported difference in *k*_*a*_ should be reflected in the *k*_nr_ values. Indeed, using TS energies, there is only a slight difference between the values of *k*_nr_(*T*) for complex **1** obtained with models **a** and **b** (1.77 × 10^6^ s^–1^) and with models **c** and **d** (2.24 × 10^6^ s^–1^) (Table 4). This is clearly the result of the much higher *k*_nr_(*T*) values associated with the ^3^MC_eq 2_ decay than to the other ^3^MC-mediated paths. Moreover, minor changes are found when recalculating the *k*_nr_(*T*) values using the energy barriers based on CI-NEB structures (1.14 × 10^6^ s^–1^ for models **a** and **b**; 1.70 × 10^6^ s^–1^ for models **c** and **d**) (Table 4).

**Table 4:** Temperature-Dependent Nonradiative Decay Rate Constant *k*_nr_(*T*) Computed at the PBE0/def2-SVP CPCM (CH_3_CN) Level of Theory for Complexes **1** and **2** Using Models **a, b, c**, and **d**, and Evaluating the *E*_*a*_/*E*_*b*_ Barriers Either Computing the Corresponding TS or Using the CI-NEB Structure

		*k*_nr_ **(*T*) (s^–1^)**	
**complex**	**model**	**NAST (TS)**	**NAST (CI-NEB)**
**1**	**a**	1.77 × 10^6^	1.14 × 10^6^
	**b**	1.77 × 10^6^	1.14 × 10^6^
	**c**	2.24 × 10^6^	1.70 × 10^6^
	**d**	2.24 × 10^6^	1.70 × 10^6^
**2**	**a**	2.69 × 10^3^	2.69 × 10^3^
	**b**	4.67 × 10^3^	3.61 × 10^3^
	**c**	3.38 × 10^3^	2.64 × 10^3^
	**d**	4.61 × 10^3^	3.46 × 10^3^

Regarding complex **2**, two nonequivalent ^3^MC states (hereafter ^3^MC_ax 1_ and ^3^MC_eq 1_) were optimized together with their corresponding MECPs (hereafter (^3^MC_ax 1_/S_0_)_stc‑mecp_ and (^3^MC_eq 1_/S_0_)_stc‑mecp_, see Section S2 in the Supporting Information). The simple estimation of their relative importance based on their energies is here less clear because the ^3^MC_ax 1_ minimum is only 0.06 eV below the ^3^MC_eq 1_ minimum, and their MECPs are almost degenerated (see Table 1). While for complex **1** the accessibility of the different ^3^MC states was assessed for all T_1_ minima, for complex **2**, we will focus only on the ^3^MLCT minimum because its energy is lower than that of the ^3^LC_ppy_ minimum by 0.07 eV (Table 1). The evaluation of the corresponding CI-NEBs points to the ^3^MC_eq 1_ as the most accessible ^3^MC state, since the corresponding *E*_*a*_ values are equal to 0.56 and 0.65 eV for the (^3^MLCT)_min_/(^3^MC_eq 1_)_min_ and (^3^MLCT)_min_/(^3^MC_ax 1_)_min_ paths, respectively (Table 3). The CI-NEB *E*_*a*_ barrier from the ^3^MLCT minimum to the ^3^MC_eq 1_ minimum is 0.05 eV above the adiabatic energy difference (0.51 eV, Table 3) and, therefore, does not need to be refined by the computation of the corresponding TS. However, this does not apply for the CI-NEB barrier between the ^3^MLCT and ^3^MC_ax 1_ minima because it is 0.20 eV above their adiabatic energy difference (0.45 eV, Table 3). The refinement of the latter by calculating the corresponding TS lowers the barrier by only 0.03 eV (making it equal to 0.62, Table 3), thus confirming the greater accessibility of the ^3^MC_eq 1_ state compared to the ^3^MC_ax 1_ state. These results emphasize the fact that judging the accessibility of minima on the basis of the sole consideration of adiabatic energy differences can be misleading. This is the case for complex 2, for which the most accessible ^3^MC_eq 1_ minimum is actually 0.06 eV above the ^3^MC_ax 1_ (Table 1).

The 0.09 eV difference found for complex **2** between the energy barrier leading from the ^3^MLCT minimum to the most accessible ^3^MC_eq 1_ minimum compared to the ^3^MC_ax 1_ structure is however not as large as the one characterizing complex **1** (of at least 0.18 eV). This determines that the corresponding *k*_*a*_ values are actually not so different (2.08 × 10^3^ and 1.18 × 10^3^ s^–1^, Table S7), and then the inclusion of the ^3^MC_ax 1_ minimum has a non-negligible effect. As a consequence, a more substantial difference appears for the values of *k*_nr_(*T*) of complex **2** obtained with models **a** and **b** (2.69 × 10^3^ and 4.67 × 10^3^ s^–1^) and with models **c** and **d** (3.38 × 10^3^ and 4.61 × 10^3^ s^–1^). As for complex **1**, no big difference is observed for complex **2** when the *k*_nr_(*T*) constants are computed using the barriers obtained from CI-NEB geometries instead of TS structures (see Table 4). This is attributable to the fact that only one of the four TSs was indeed characterized, while the remaining three were approximated with the CI-NEB structures (see Table 3).

### Emission Quantum Yield (Φ_em_)

Once analyzed and computed the three rate constants that influence the emission quantum yield, we can calculate Φ_em_ using eq 6. Table 5 presents the values computed for Φ_em_ using the four approaches sketched in Figure 2 (models **a**–**d**) and evaluating the *E*_*a*_/*E*_*b*_ barriers with both TS and CI-NEB geometries (Figure 3), as well as the corresponding experimental data.

**Table 5:** Emission Quantum Yield (Φ_em_) Computed at the PBE0/def2-SVP CPCM (CH_3_CN) Level of Theory for Complexes **1** and **2** Using Models **a**, **b**, **c**, and **d**, and Evaluating the *E*_*a*_/*E*_*b*_ Barriers Either Computing the Corresponding TS or Using the CI-NEB Structure; the Experimental Φ_em_ Values Are Also Included

**complex**	**model**	**Φ**_**em**_ **(%)**	
		**TS**	**CI-NEB**
**1**	**a**	5.30	8.04
	**b**	5.30	8.04
	**c**	14.97	18.81
	**d**	14.97	18.83
	experimental^a^	20.40	
**2**	**a**	94.41	94.41
	**b**	93.99	94.22
	**c**	90.59	91.02
	**d**	90.34	90.57
	experimental^b^	71.00	

a Experimental data extracted from ref 22.

b Experimental data extracted from ref 23.

As discussed above, theoretical calculations predict for complex **1** that (1) it is important to include all T_1_ minima to properly describe both *k*_*rad*_ and *k*_*ISC*_, and (2) the effect of including all the ^3^MC states and not only the most accessible one on the *k*_nr_(*T*) value is negligible. Consequently, similar Φ_em_ values should be expected using models **a** and **b**, on one hand, and using models **c** and **d**, on the other hand, but a significant difference should emerge when comparing both groups. This is indeed the case, as the Φ_em_ calculated according to models **a**, **b**, **c**, and **d** using TS structures is equal to 5.30, 5.30, 14.97, and 14.97%, respectively (Table 5), better approaching the experimental value of 20.40% when using the more complex models **c** and **d**. The computed Φ_em_ values are slightly higher when using the more approximate CI-NEB structures.

For complex **2**, an almost opposite situation is obtained. Due to the non-negligible separation between the lowest T_1_ and the other T_1_ minima (0.07 eV), the inclusion of the latter is not significantly affecting *k*_rad_ and *k*_ISC_, since the population of the lowest T_1_ is predominant when performing the Boltzmann average. On the other hand, the relatively low separation between the most accessible and the other ^3^MC states and the larger separation between the lowest and higher T_1_ minima determine that both the number of T_1_ minima and ^3^MC states has a non-negligible effect on *k*_nr_(*T*). This situation is however not reflected in the value of the Φ_em_, which remains mostly constant when using models **a** (94.41%), **b** (93.99%), **c** (90.59%), and **d** (90.34%) (Table 5). The answer to this behavior is simple: for complex **2**, the value of *k*_ISC_ (about 10^4^ s^–1^) is always significantly larger than the value of *k*_nr_(*T*) (10^3^ s^–1^), so possible variations in *k*_nr_(*T*) are covered by the higher value of *k*_ISC_. This also determines that the changes of the *k*_nr_(*T*) values resulting from the evaluation of the *E*_*a*_/*E*_*b*_ barriers on the basis of CI-NEB structures (Table 4) do not manifest in the corresponding Φ_em_ values, which remain almost equal to the data with TS barriers (see Table 5).

## Conclusions

In the present contribution, we have explored the importance of including all possible radiative and nonradiative decay paths for the computation of the emission quantum yield of the two green-emitting cyclometalated Ir­(III) complexes, [Ir­(ppy)_2_(pyim)]^+^ (**1**) and [Ir­(diFppy)_2_(dtb-bpy)]^+^ (**2**).

For complex **1**, in order to obtain a proper evaluation of its Φ_em_, it is important to include all T_1_ minima, while the inclusion of just the most accessible ^3^MC state is sufficient. Moreover, the ^3^MC-mediated nonradiative decay path can be described using CI-NEBs structures, with no need to obtain actual TSs. For complex **2** instead, due to the significant separation between the lower T_1_ minima (0.07 eV), which determines that nearly 90% of the population remains in the lowest ^3^MCLT state, and to the fact that *k*_ISC_ is 1 order of magnitude larger than *k*_nr_(*T*), no significant difference is observed in the Φ_em_ obtained accordingly to the four presented models, independently on how the *E*_*a*_/*E*_*b*_ barriers separating the T_1_ and ^3^MC states are evaluated.

Would it have been possible to predict these results? The answer is unfortunately no, since only through the computation of all T_1_ and ^3^MC minima did we arrive at this conclusion. The situation is however not so dramatic because the evaluation of all T_1_ and ^3^MC minima for a general [Ir­(C^N)_2_(N^N)]^+^ complex is not the bottleneck in the theoretical description of its photophysics. The most delicate task is instead the characterization of the TSs connecting the different minima, which sometime appears to be impossible to converge, as indeed occurs for some of the here presented paths. With the present results we can however arrive at the important conclusion that if CI-NEBs (which are far easier to obtain than TSs) are only slightly above the corresponding ^3^MC minima (<0.05 eV), the evaluation of the corresponding TSs is not worth it, since the energy refinement derived from the energy difference between CI-NEBs and TSs will be too small to be appreciable at any level. Additionally, when the value of *k*_ISC_ clearly dominates the nonradiative behavior of the complex, it will not even be particularly useful to characterize the ^3^MC-mediated radiationless routes.

## Supplementary Material



## Notes

Computational details regarding the calculation of rate constants with the NAST software (Section S1), theoretical characterization for complexes **1** and **2** (Section S2), radiative and nonradiative rate constants employed for the four models used in the present contribution (Section S3), and optimized geometries of all the critical points (minima, TS, CI-NEB, and MECPs) for complexes **1** and **2** (Section S4) (PDF)

## ∥

Department of Chemistry, New York University, New York, New York 10003, United States
