# Supplementary material for: On the In Silico Evaluation of the Emission Quantum Yield of Cyclometalated Ir(III) Complexes
Source: Inorg Chem. 2025 Apr 16;64(16):8313–21. doi: 10.1021/acs.inorgchem.5c00700 (PMC12381845; doi:10.1021/acs.inorgchem.5c00700)
Supplement: Supplementary file 1 [file ic5c00700_si_001.pdf]

Supporting Information
(Total of 66 pages)
for

On the In-Silico Evaluation of the Emission Quantum Yield of Cyclometalated Ir(III) Complexes

Iván Soriano-Díaz,[1] Alicia Omist,[1] Ilya D. Dergachev,[2] Sergey A. Varganov,[3] Enrique Ortí,[1,*] and Angelo Giussani[1,*]

[1] Institute for Molecular Science (ICMol), Universitat de València, Catedrático José Beltrán 2, 46980 Paterna, España.

[2] Department of Chemistry, New York University, New York, New York 10003, USA

[3] Department of Chemistry, University of Nevada, Reno, 1664 N. Virginia Street, Reno, NV 89557-0216, USA

Email: enrique.orti@uv.es, angelo.giussani@uv.es

Outline

1. Section S1: Computational details regarding the calculation of rate constant with the NAST software. (page S3)
2. Section S2: Full theoretical characterization of the photophysics of complexes **1** and **2**. (page S4)
   2.1. Geometrical features of the optimized ground and excited states and electronic structure characterization. (page S4)
   2.2. T_1 minima. (page S7)
   2.3. Characterization of the ^{3}MC mediated non-radiative decay process. (page S12)
3. Section S3: Computation of the radiative and non-radiative rate constants. (page S19)
4. Section S4: Additional data. (page S21)
5. Bibliography (page S66)

Section S1. Computational details regarding the calculation of rate constants with the NAST software.

We calculated the canonical TST rate constant using the following expression as implemented in the NAST program package:[1,2]

$$k(T) = \sigma \frac{k_B T}{h} \frac{Q_{\mathrm{TS}}}{Q_{\mathrm{R}}} e^{-E_{\mathrm{TS}}/k_{\mathrm{B}}T}, \tag{S1}$$

where σ is the reaction symmetry factor, T is the temperature, k_{B} is the Boltzmann constant, h is the Planck's constant, Q_{TS} and Q_{R} are partition functions of TS and reactant, respectively, and E_{TS} is the activation energy. Partition function Q can be calculated from the rovibrational density of states ρ:

$$Q(T) = \int_0^{\infty} \rho(E) e^{-E/k_{\mathrm{B}}T}\, dE. \tag{S2}$$

The rovibrational density of states ρ is calculated at the given internal energy E as the convolution of the vibrational ρ_{vib} and rotational ρ_{rot} densities of states:

$$\rho(E) = \int_0^{E} \rho_{\mathrm{vib}}\,(E - E_{\mathrm{rot}}) \rho_{\mathrm{rot}}(E_{\mathrm{rot}}) dE_{\mathrm{rot}}. \tag{S3}$$

The vibrational density of states is calculated using the direct counting of the vibrational levels populated at a given energy. The rotational density of states is calculated using the classical asymmetric top model as[1]

$$\rho_{\mathrm{rot}}(E_{\mathrm{rot}}) = \frac{4\sqrt{2E_{\mathrm{rot}}}}{h^3} \sqrt{I_{\mathrm{A}} I_{\mathrm{B}} I_{\mathrm{C}}}, \tag{S4}$$

where I_{A}, I_{B} and I_{C} are the three principal moments of inertia of a molecule.

NAST can account for the zero-point energy (ZPE) by correcting the transition state energy E_{TS} with the ZPEs of transition state and reactant:

$$E'_{\mathrm{TS}} = E_{\mathrm{TS}} + ZPE_{\mathrm{TS}} - ZPE_{\mathrm{R}}. \tag{S5}$$

Section S2. Full theoretical characterization of the photophysics of complexes 1 and 2

Geometrical features of the optimized ground and excited states and electronic structure characterization

The optimized geometry of the electronic ground state, hereafter $(S_0)_{min}$, of complexes **1** and **2** was determined at the DFT PBE0/DEF2-SVP CPCM (CH_3CN) level of theory. Table S1 summarizes the coordination bond lengths of the Ir center that keeps the octahedral disposition for this state. For both complexes, the Ir–$N_{N\wedge N}$ bond distances are around 2.2 Å, significantly longer than the distances involving the ppy ligands, which are around 2.08 and 2.01 Å for Ir–$N_{C\wedge N}$ and Ir–$C_{C\wedge N}$ bonds, respectively.

Table S1. DFT PBE0/DEF2-SVP CPCM (CH_3CN) optimized bond lengths (in Å) computed for the fully-relaxed minima of the different electronic states characterized for complexes **1** and **2**.

Minimum	Bond	Complex	
		1	2
$(S_0)_{min}$	Ir–$N_{1-N\wedge N}$	2.178	2.167
	Ir–$N_{2-N\wedge N}$	2.236	2.168
	Ir–$N_{3-C\wedge N}$	2.071	2.060
	Ir–$N_{6-C\wedge N}$	2.072	2.059
	Ir–$C_{4-C\wedge N}$	2.009	2.008
	Ir–$C_{5-C\wedge N}$	2.014	2.008
$(^3MLCT)_{min}$	Ir–$N_{1-N\wedge N}$	2.155	2.155
	Ir–$N_{2-N\wedge N}$	2.179	2.159
	Ir–$N_{3-C\wedge N}$	2.059	2.059
	Ir–$N_{6-C\wedge N}$	2.062	2.059
	Ir–$C_{4-C\wedge N}$	1.991	1.998
	Ir–$C_{5-C\wedge N}$	1.983	1.991
$(^3LC_{ppy\ 1})_{min}$	Ir–$N_{1-N\wedge N}$	2.179	2.185
	Ir–$N_{2-N\wedge N}$	2.241	2.175
	Ir–$N_{3-C\wedge N}$	2.043	2.036
	Ir–$N_{6-C\wedge N}$	2.074	2.068
	Ir–$C_{4-C\wedge N}$	1.963	1.987
	Ir–$C_{5-C\wedge N}$	2.004	2.005
$(^3LC_{ppy\ 2})_{min}$	Ir–$N_{1-N\wedge N}$	2.185	----
	Ir–$N_{2-N\wedge N}$	2.242	----
	Ir–$N_{3-C\wedge N}$	2.073	----
	Ir–$N_{6-C\wedge N}$	2.043	----
	Ir–$C_{4-C\wedge N}$	1.998	----
	Ir–$C_{5-C\wedge N}$	1.966	----
$(^3MC_{ax\ 1})_{min}$	Ir–$N_{1-N\wedge N}$	2.192	2.213
	Ir–$N_{2-N\wedge N}$	2.208	2.173
	Ir–$N_{3-C\wedge N}$	2.274	2.244
	Ir–$N_{6-C\wedge N}$	2.551	2.521
	Ir–$C_{4-C\wedge N}$	2.022	2.026
	Ir–$C_{5-C\wedge N}$	2.019	2.018
$(^3MC_{ax\ 2})_{min}$	Ir–$N_{1-N\wedge N}$	2.168	----
	Ir–$N_{2-N\wedge N}$	2.313	----
	Ir–$N_{3-C\wedge N}$	2.521	----
	Ir–$N_{6-C\wedge N}$	2.228	----
	Ir–$C_{4-C\wedge N}$	2.017	----
	Ir–$C_{5-C\wedge N}$	2.026	----
$(^3MC_{eq\ 1})_{min}$	Ir–$N_{1-N\wedge N}$	3.252	2.207
	Ir–$N_{2-N\wedge N}$	2.521	3.356
	Ir–$N_{3-C\wedge N}$	2.059	2.053
	Ir–$N_{6-C\wedge N}$	2.054	2.057
	Ir–$C_{4-C\wedge N}$	2.021	2.038
	Ir–$C_{5-C\wedge N}$	2.045	2.023
$(^3MC_{eq\ 2})_{min}$	Ir–$N_{1-N\wedge N}$	2.189	----
	Ir–$N_{2-N\wedge N}$	3.294	----
	Ir–$N_{3-C\wedge N}$	2.058	----
	Ir–$N_{6-C\wedge N}$	2.050	----
	Ir–$C_{4-C\wedge N}$	2.042	----
	Ir–$C_{5-C\wedge N}$	2.016	----

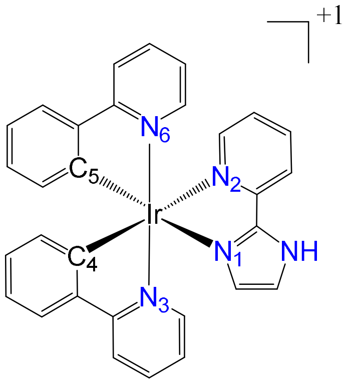

1

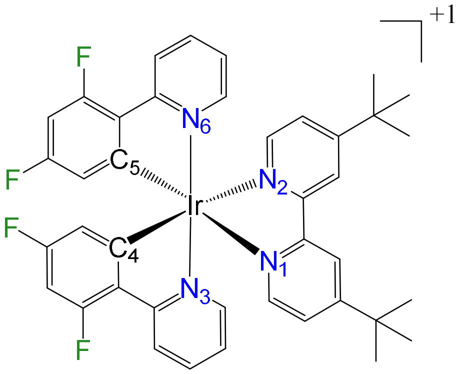

2

Figures S1 and S2 show the main frontier molecular orbitals (MOs) involved in the six lowest excited triplet states (see Table S2) computed at the $(S_0)_{min}$ geometry for complex **1** and **2**, respectively. The lowest e_g* MO is also included. As expected, the highest-occupied molecular orbital (HOMO) presents a mixed nature involving the π electrons of the phenyl rings of the main ligand and the *d* orbitals of the metal. In contrast, the lowest-unoccupied molecular orbital (LUMO) corresponds, almost exclusively, to the π* LUMO orbital of the ancillary ligand. The HOMO-LUMO gap is 3.94 eV for complex **1** and 4.00 eV for complex **2**.

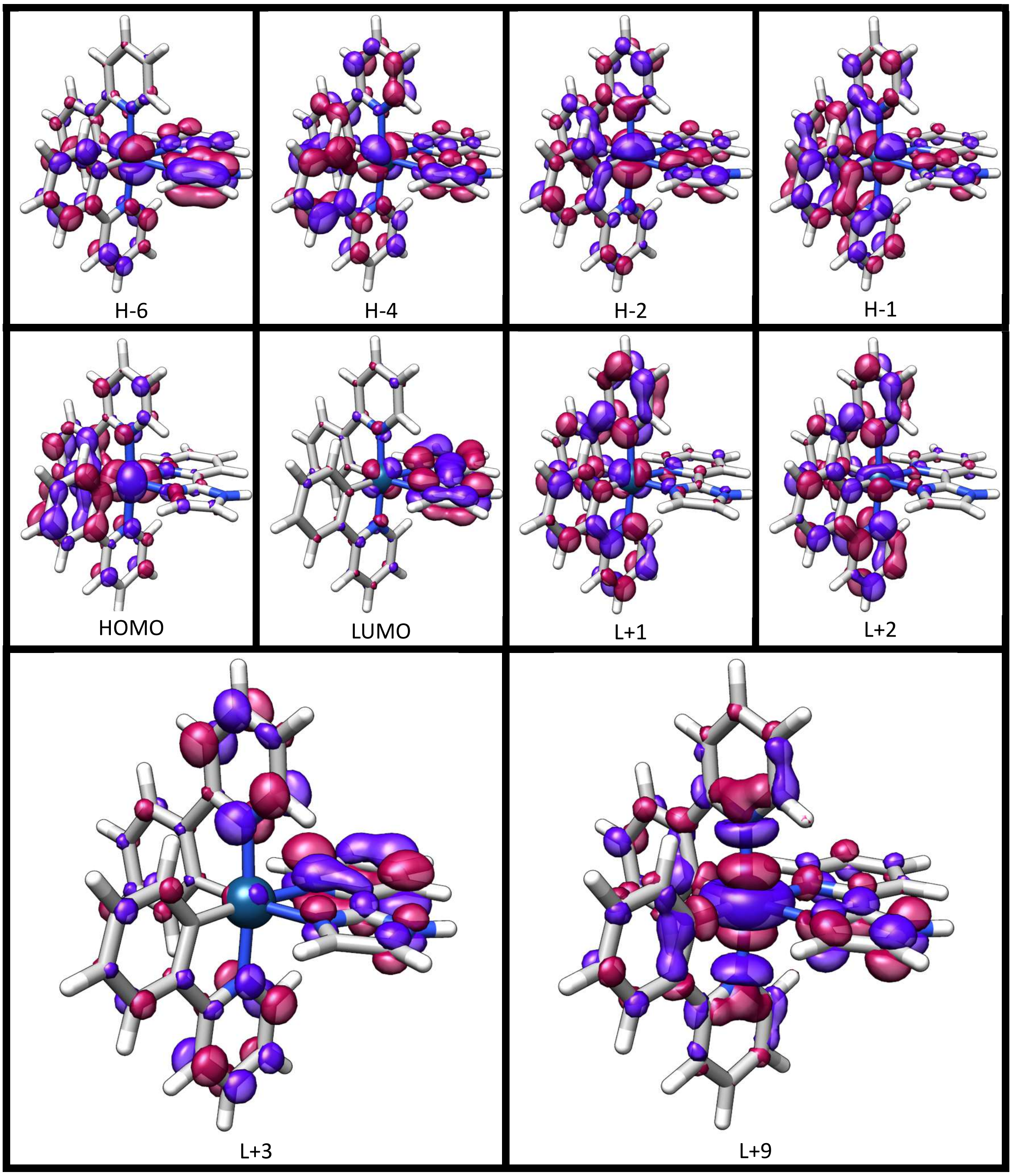

Figure S1. Molecular frontiers orbitals (isosurface contour plots, ±0.03 a.u.) computed at the PBE0/DEF2-SVP CPCM (CH_3CN) level on the $(S_0)_{min}$ geometry for complex **1**.

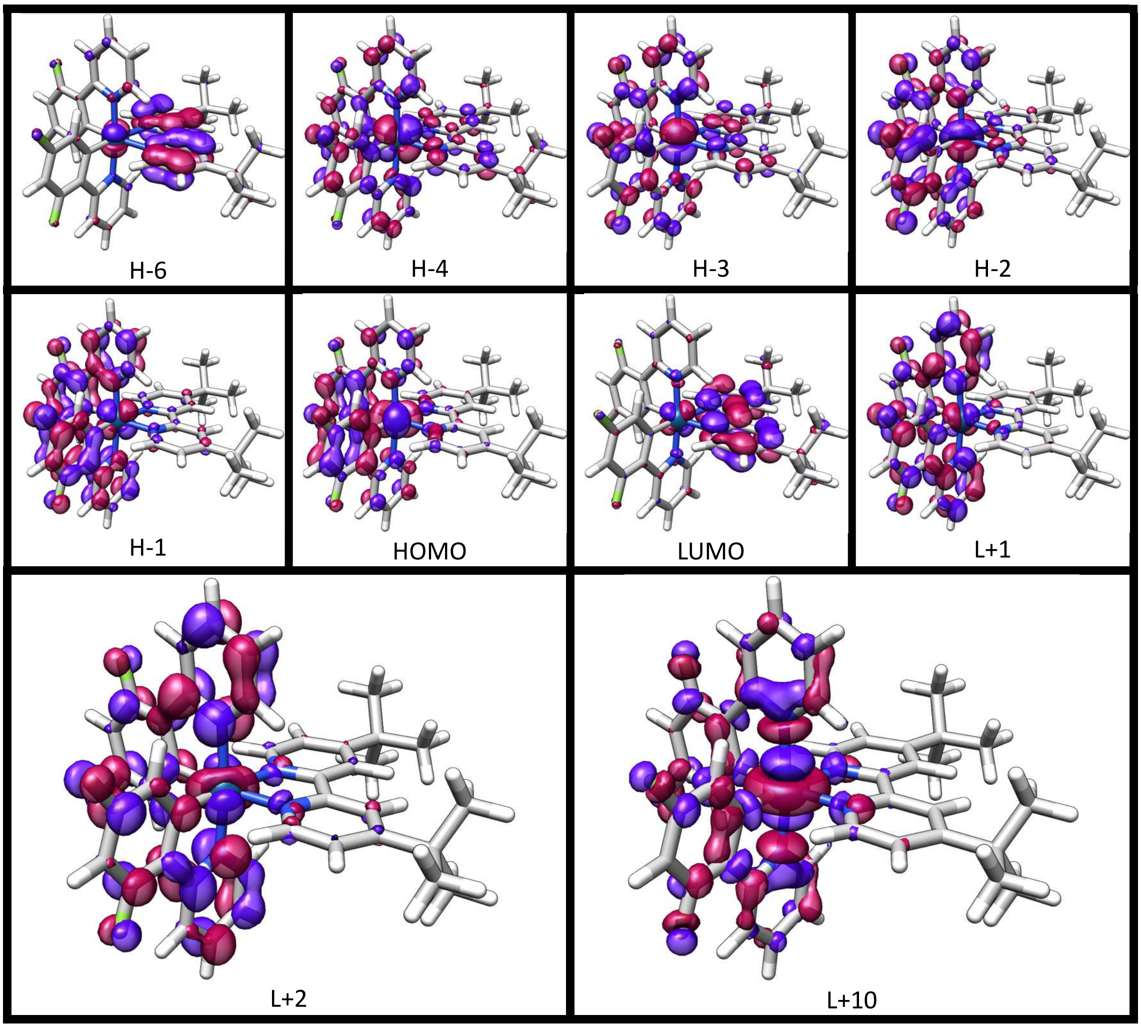

Figure S2. Molecular frontiers orbitals (isosurface contour plots, ±0.03 a.u.) computed at the PBE0/DEF2-SVP CPCM (CH_3CN) level on the $(S_0)_{min}$ geometry for complex **2**.

Table S2 collects the TD-DFT energies and electronic nature calculated for the six lowest triplet excited states and the lowest ^{3}MC state at the ground state minima. The electronic nature was established with the help of the fragment-based analysis performed with TheoDORE software and the MOs that characterize the main monoexcitations describing each state.

For complex **1** the two first triplet excited states present a ^{3}LC nature and are localized on the main C^N ligands. The third triplet is also of ^{3}LC character but now located on the ancillary N^N ligand. The first ^{3}MLCT/^{3}LLCT state (hereafter ^{3}MLCT) corresponds to the fourth state in the Franck-Condon region. As expected, the energies of the two $^3LC_{C^\wedge N}$ states are similar (2.72 and 2.75 eV, respectively). The $^3LC_{N^\wedge N}$ state rise up to 2.89 eV and the ^{3}MLCT state is at 3.06 eV at this geometry and theory level. These states indeed present a mixed nature since all of them show non-negligible MLCT and LC contributions (see Table S2). For instance, the $^3LC_{C^\wedge N}$ states present a MLCT contribution around 10−15%. A similar distribution of triplet states is computed for complex **2** with two $^3LC_{C^\wedge N}$ states as the lowest triplets (2.86 and 2.88 eV), and the $^3LC_{N^\wedge N}$ and ^{3}MLCT states slightly higher in energy at 2.95 and 3.13 eV, respectively (Table S2). The relevant role of the $^3LC_{C^\wedge N}$ states on the photophysics of both complex **1** and **2** can be therefore expected. To prove that, the geometries of these two states and the ^{3}MLCT state were optimized for both complexes at the unrestricted (UDFT) level of theory. However, despite several efforts, the $^3LC_{N^\wedge N}$ was impossible to optimize.

Table S2. TD-DFT PBE0/DEF2-SVP (CH_3CN) characterization of the sixth lowest triplet excited states and the lowest ^{3}MC state calculated on the $(S_0)_{min}$ geometry for complex **1** and **2**. The main contribution to the electronic nature of each state is indicated within parentheses. H and L denote HOMO and LUMO, respectively. Vertical excitation energies (*E*) and monoexcitations (contributing more than 10% to the wavefunction) were obtained directly from the ORCA software. The nature of each state is decomposed in MC, LC, MLCT, LMCT, and LLCT contributions using the TheoDORE software.

Complex	State	*E* (eV)	Monoexcitations (%)	Nature (%)				
				MC	LC	MLCT	LMCT	LLCT
1	T_1 ($^3LC_{C^\wedge N}$)	2.72	H → L + 1 (48)<br>H – 1 → L + 3 (16)	0.9	76.7	15.1	2.2	5.1
	T_2 ($^3LC_{C^\wedge N}$)	2.75	H → L + 3 (38)<br>H – 1 → L + 2 (22)	0.8	81.3	12.4	2.4	3.1
	T_3 ($^3LC_{N^\wedge N}$)	2.89	H – 2 → L (25)<br>H – 6 → L (19)<br>H → L (15)<br>H – 4 → L (12)	0.4	77.7	14.3	1.3	6.3
	T_4 (^{3}MLCT)	3.06	H → L (79)	1.3	15.4	39.1	1.6	42.6
	T_5	3.22	H → L + 1 (37)	1.4	54.7	28.1	1.3	14.5
	T_6	3.24	H → L + 3 (47)	1.6	52.1	30.3	1.5	14.5
	T_{27} (^{3}MC)	4.39	H → L + 9 (40)	34.4	9.9	26.3	11.3	18.2
2	T_1 ($^3LC_{C^\wedge N}$)	2.86	H → L + 1 (36)<br>H – 1 → L + 2 (26)<br>H – 2 → L + 1 (14)	0.6	83.2	10.5	2.1	3.5
	T_2 ($^3LC_{C^\wedge N}$)	2.88	H – 1 → L + 1 (34)<br>H → L + 2 (28)<br>H – 2 → L + 2 (13)	0.6	86.2	8.6	2.3	2.3
	T_3 ($^3LC_{N^\wedge N}$)	2.95	H – 6 → L (43)<br>H – 3 → L (19)<br>H → L (11)	0.4	80.6	13.0	1.1	4.9
	T_4 (^{3}MLCT)	3.13	H → L (85)	1.3	13.5	39.8	1.4	44.1
	T_5	3.41	H – 4 → L (42)<br>H – 1 → L (21)	2.2	37.1	45.6	1.6	13.5
	T_6	3.42	H → L + 1 (48)<br>H – 2 → L + 1 (17)	1.8	49.9	31.2	1.7	15.3
	T_{34} (^{3}MC)	4.59	H → L + 10 (39)<br>H – 3 → L + 10 (24)	38.2	11.1	31.0	11.2	8.4

T_1 minima

The radiative decay path was studied by the optimization of the emitting states at the UPBE0 level of theory for complexes **1** and **2**. For complex **1** a total of three different triplet minima were obtained (hereafter $(^3MLCT)_{min}$, $(^3LC_{ppy\,1})_{min}$, and $(^3LC_{ppy\,2})_{min}$). Due to the molecular asymmetry of the N^N ligand, the two $^3LC_{ppy}$ states in complex **1** are not equivalent. The ^{3}MLCT state at its minimum-energy geometry indeed shows a highly mixed character with a 29.5% $LC_{N^\wedge N}$, 33.7% MLCT, and 33.8% LLCT (see Table S3). For this state, the fragment analysis (Table S3), the spin density plots (Figure S3), and the NTO analysis (Figure S4) indicate that there is one unpaired electron residing on the phenyl rings of the C^N ligands, the ancillary N^N ligand, and the Ir atom, and the other unpaired electron on the N^N ligand, confirming the ^{3}MLCT/^{3}LLCT/^{3}LC nature. The $^3LC_{ppy}$ states also present MLCT contributions of 13.5 and 13.8% for $^3LC_{ppy\,1}$ and $^3LC_{ppy\,2}$ respectively (Table S3). Fragment analysis, spin density plots, and NTO analysis show that both unpaired electron mainly reside on one ppy ligand, with a small contribution from the metal, thus confirming the ^{3}LC/^{3}MLCT nature.

For complex **2**, two emitting states, $(^3MLCT)_{min}$ and $(^3LC_{ppy\,1})_{min}$, were characterized. For the $(^3MLCT)_{min}$ state, there is a discrepancy between the electronic nature predicted by the spin density, the NTO, and the TheoDORE results. According to the spin density (0.569, 0.203, 0.199, and 1.03 unpaired electrons for Ir, the two ppy ligands, and bpy, respectively, see Figure S5) it corresponds to a prototypical ^{3}MLCT/^{3}LLCT state. However, according to the NTO (Figure S6) and the fragment-based analysis from TheoDORE (Table S3), this state mostly presents a $^3LC_{N^\wedge N}$ nature (61.5, 20.9, and 15.3% for LC, MLCT and LLCT, respectively, see Table S3). This difference can be attributed as the latter rely on the electron transition density matrix calculated at TD-DFT level, whereas the former comes from the UDFT calculation. The $(^3LC_{ppy\,1})_{min}$ state presents small MLCT contributions (86.1 and 7.9% for LC and MLCT, respectively, see Table S3). For this state, the fragment-based analysis provided by TheoDORE software agrees with the spin density (Figure S5) and NTO results (Figure S6).

Table S3. TheoDORE fragment analysis for the characterized T_1 minima of complexes **1** and **2**.

Complex	State	Nature (%)				
		MC	LC	MLCT	LMCT	LLCT
1	$(^3MLCT)_{min}$	1.2	29.5	33.7	1.8	33.8
	$(^3LC_{ppy\,1})_{min}$	0.8	78.4	13.5	3.0	4.3
	$(^3LC_{ppy\,2})_{min}$	0.9	77.4	13.8	3.2	4.8
	$(^3MC_{ax\,1})_{min}$	47.5	3.7	27.3	15.5	6.0
	$(^3MC_{ax\,2})_{min}$	46.6	4.1	27.9	15.3	6.2
	$(^3MC_{eq\,1})_{min}$	26.3	11.2	29.8	18.5	14.2
	$(^3MC_{eq\,2})_{min}$	24.9	12.2	29.6	18.5	14.8
2	$(^3MLCT)_{min}$	0.7	61.5	20.9	1.6	15.3
	$(^3LC_{ppy\,1})_{min}$	0.5	86.1	7.9	2.9	2.6
	$(^3MC_{ax\,1})_{min}$	45.6	4.1	27.9	15.7	6.6
	$(^3MC_{eq\,1})_{min}$	22.5	9.7	28.0	22.2	17.6

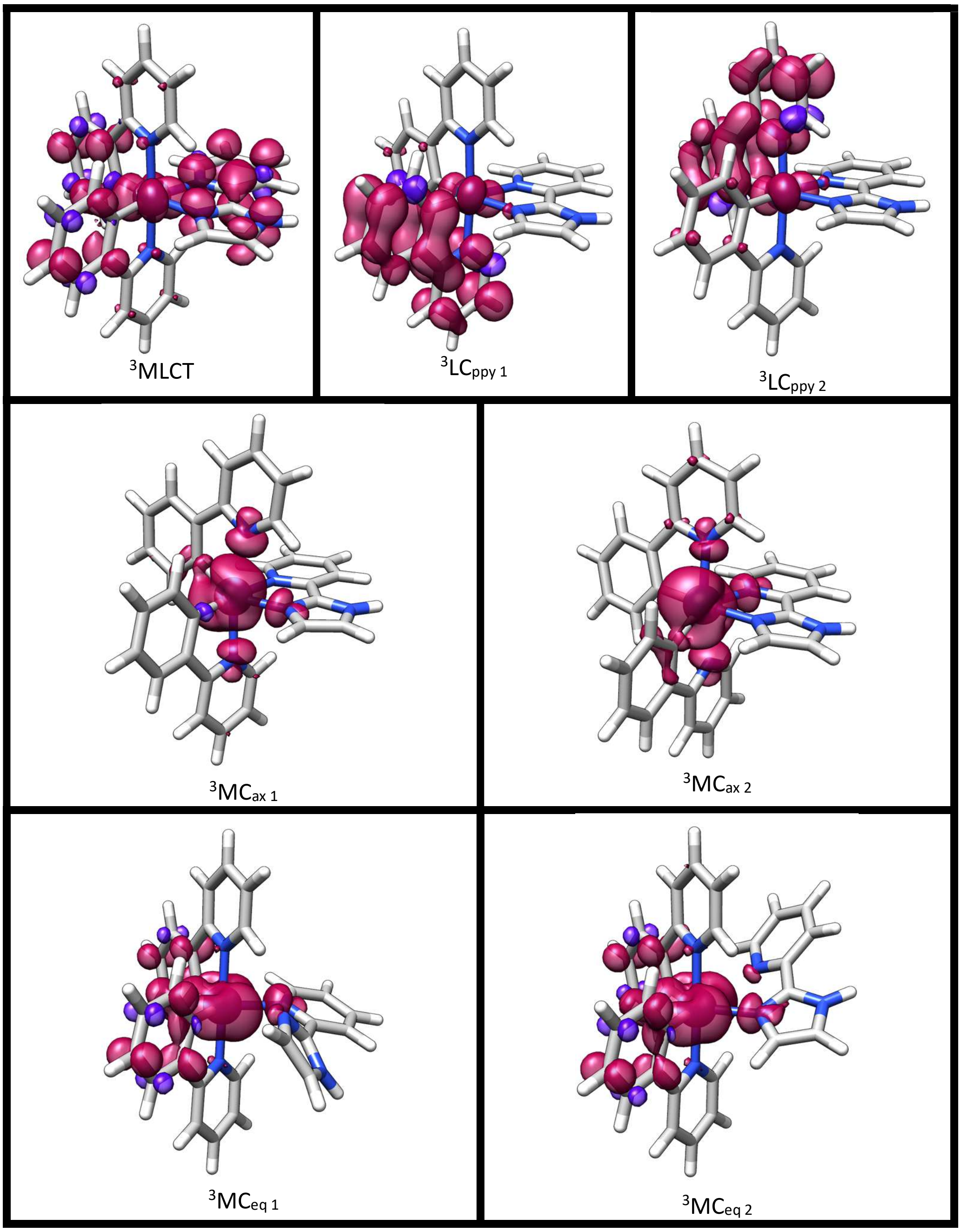

Figure S3. Spin densities (isosurface contour plots, ±0.003 a.u.) computed at the UPBE0/DEF2-SVP CPCM (CH_3CN) level for all the T_1 and ^{3}MC triplet states minima characterized for complex **1**.

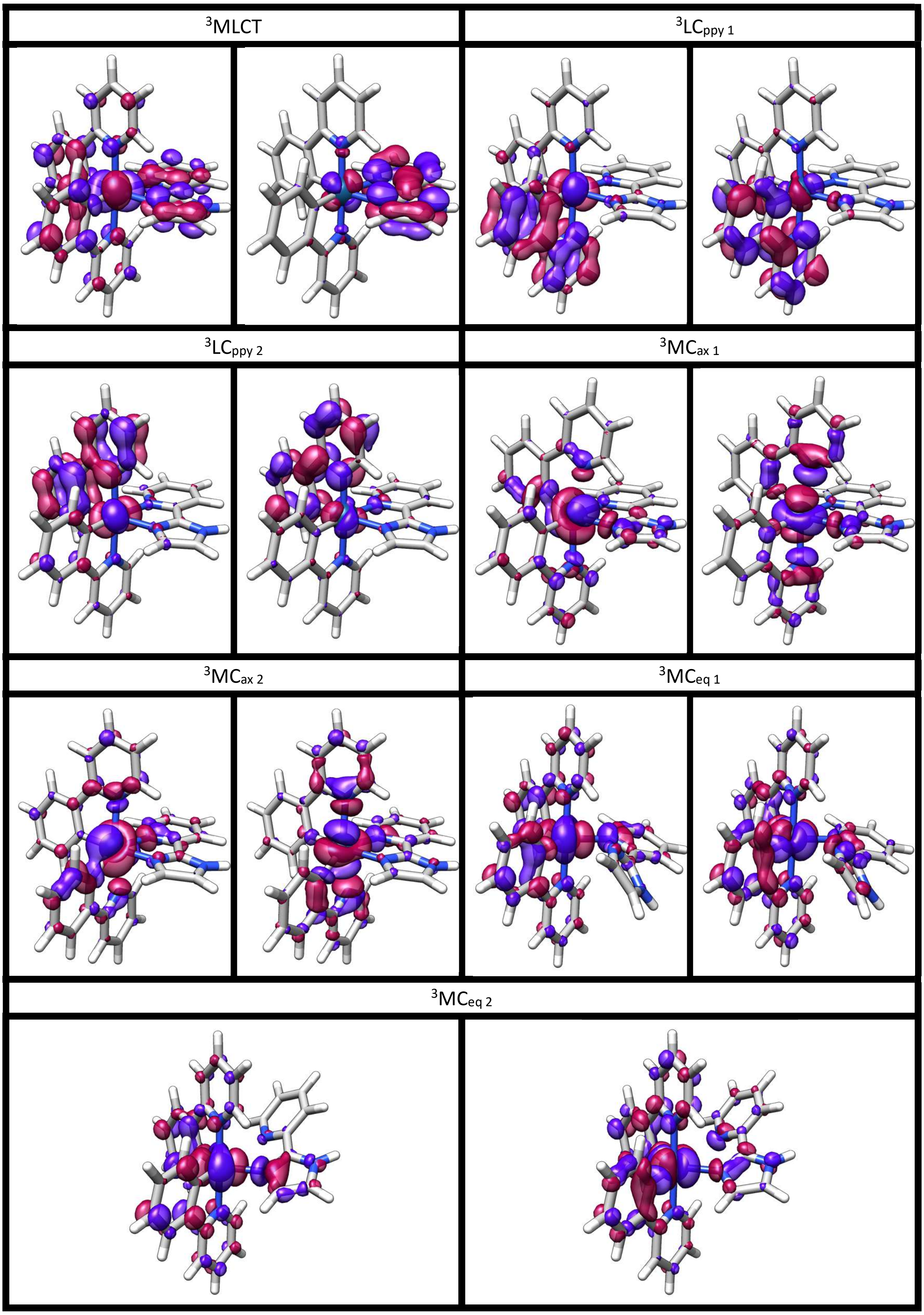

Figure S4. Natural transition orbitals (NTOs) (isosurface contour plots, ±0.03 a.u.) that characterize the electronic transition to the the T_1 and ^{3}MC states from S_0 state of complex **1** computed at the respective optimized minima at the PBE0/DEF2-SVP CPCM (CH_3CN) level of theory.

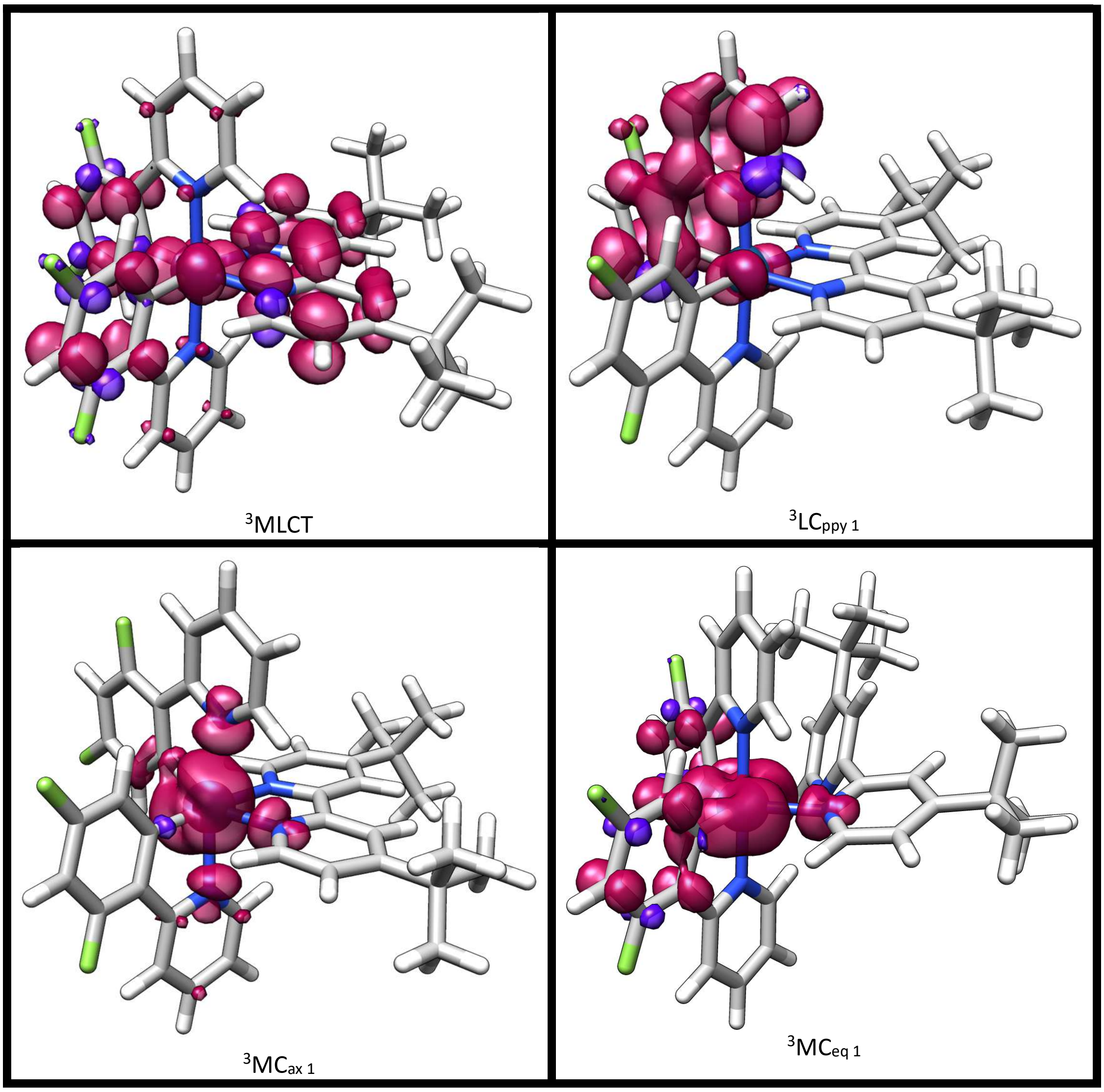

Figure S5. Spin densities (isosurface contour plots, ±0.003 a.u.) computed at the UPBE0/DEF2-SVP CPCM (CH_3CN) level for all the T_1 and ^{3}MC triplet states minima characterized for complex **2**

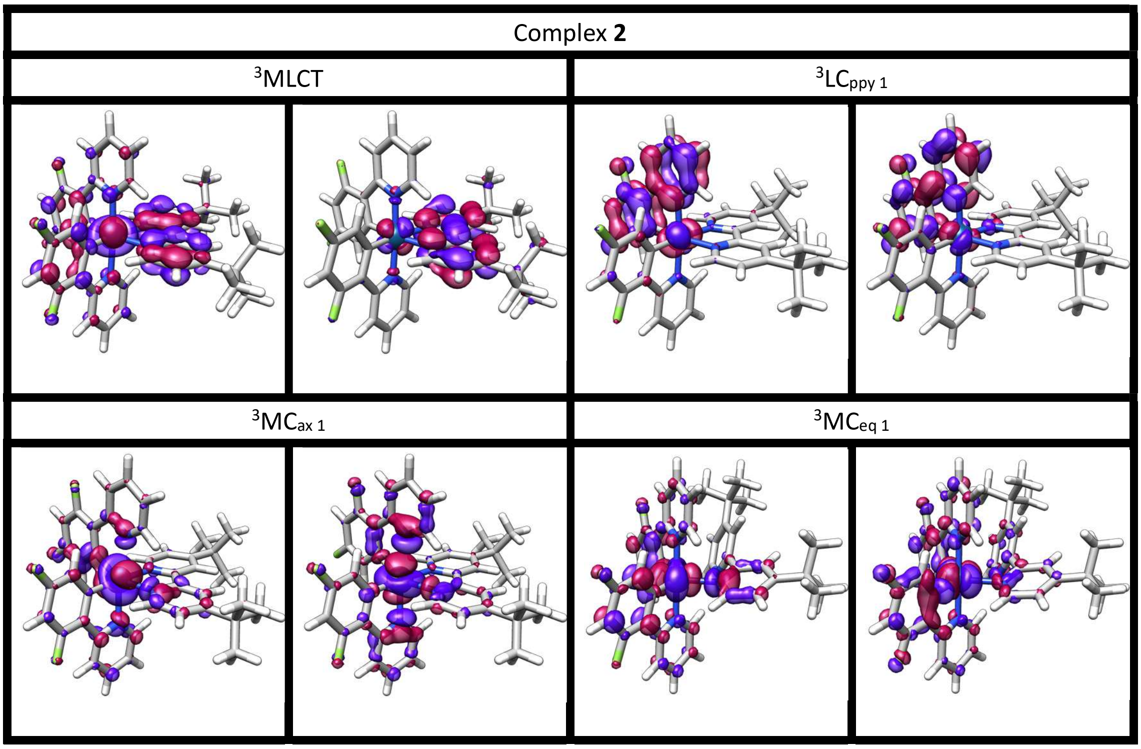

Figure S6. Natural transition orbitals (NTOs) (isosurface contour plots, ±0.03 a.u.) that characterize the electronic transition to the T_1 and ^{3}MC states from S_0 state of complex **2** computed at the respective optimized minima at the PBE0/DEF2-SVP CPCM (CH_3CN) level of theory.

The emission energies (E_{em}) from the obtained T_1 minima (computed as the vertical energy difference between the triplet and the singlet state calculated at the UDFT and DFT level of theory, respectively, at the respective $(T_1)_{min}$) agree with the experimental emission energies, then supporting their involvement in the emission process of the two complexes (see Table S4)

Table S4. Vertical emission energies (E_{em}), computed at the UPBE0/DEF2-SVP-CPCM(CH_3CN) level of theory, from the characterized T_1 minima of complexes **1** and **2**. The corresponding experimental values are also reported.

Complex	State	E_{em} (eV)
**1**	$(^3MLCT)_{min}$	2.38
	$(^3LC_{ppy\ 1})_{min}$	2.38
	$(^3LC_{ppy\ 2})_{min}$	2.38
	Experimental[a]	2.40
**2**	$(^3MLCT)_{min}$	2.47
	$(^3LC_{ppy\ 1})_{min}$	2.49
	Experimental[b]	2.37

[a] Experimental data from reference [3]
[b] Experimental data from reference [4]

Characterization of the ^{3}MC-mediated non-radiative decay processes

A total of four ^{3}MC states were localized and optimized for complex **1**, two asymmetrical $^3MC_{ax}$ (hereafter $^3MC_{ax\ 1}$ and $^3MC_{ax\ 2}$) and two $^3MC_{eq}$ states (hereafter, $^3MC_{eq\ 1}$ and $^3MC_{eq\ 2}$). Two minima have to be considered for each ^{3}MC state due to the asymmetry of the N^N ligand. For complex **2**, two asymmetric ^{3}MC states were first optimized ($^3MC_{ax\ 1}$ and $^3MC_{eq\ 1}$), for which only Ir–N bond (Ir-$N_{C\wedge N}$ or Ir-$N_{N\wedge N}$, respectively) is lengthened. The symmetrical $^3MC_{ax}$ state, in which both Ir–$N_{C\wedge N}$ bonds are simultaneously elongated, was also optimized for complex **2**. Nevertheless, this state was not considered for neither of the two complexes since, according to our previous work,[5] this minimum normally presents a higher adiabatic energy difference (ΔE_{adi}) and MECPs. Therefore, it should be much less relevant to the photophysical behavior of complexes **1** and **2** than the others MC minima.

To optimize the $^3MC_{ax}$ states, one Ir–$N_{C\wedge N}$ bond was elongated up to 2.6 Å starting from the $(^3MLCT)_{min}$ geometry. The optimized length converged 2.551 and 2.521 Å for $^3MC_{ax\ 1}$ and $^3MC_{ax\ 2}$ states, being 0.489 and 0.462 longer than the respective bond in the S_0 state (see Table S1). Their ΔE_{adi} respect to the lowest

emitting state is 0.46 and 0.45 eV for $^3MC_{ax\,1}$ and $^3MC_{ax\,2}$, respectively(see Table S5). For complex **2**, the Ir–N_{C^N} optimized distance of the $^3MC_{ax\,1}$ state is elongated by 0.462 Å up to 2.521 Å (Table S1), and the ΔE_{adi} is 0.45 eV (Table S5). The spin densities calculated (Figures S3 and S5), the fragment-based analysis (Table S3), and the NTO analysis (Figures S4 and S6) agree with the primary ^{3}MC nature of these states. However, non-negligible ^{3}MLCT and ^{3}LMCT contributions are also present.

To optimize the $^3MC_{eq}$ states, one Ir–N_{N^N} bond was elongated starting from the $(^3MLCT)_{min}$ geometry, and the corresponding azole or pyridine ring was rotated up to almost 90º to reduce the coordination around the Ir center from 6 to 5. For complex **1**, the Ir–N_{N^N} bond length increases by 1.097 and 1.115 Å up to 3.252 and 3.294 Å for $(^3MC_{eq\,1})_{min}$ and $(^3MC_{eq\,2})_{min}$ states, respectively (Table S1). The respective ΔE_{adi} values are 0.53 and 0.34 eV for $(^3MC_{eq\,1})_{min}$ and $(^3MC_{eq\,2})_{min}$ (Table S5). For complex **2**, the Ir–N_{N^N} bond elongates by 1.197 Å up to 3.356 Å with a ΔE_{adi} value of 0.51 eV. The spin density (Figure S3), the fragment-based analysis (Table S3), and the NTO analysis (Figure S4) agree with the primary ^{3}MC nature of these states. However, non-negligible contributions from ^{3}MLCT and ^{3}LMCT are also observed (Table S3) for both complexes.

To locate the MECPs connecting the triplet ^{3}MC states and the singlet ground state S_0, the ^{3}MC optimized minima were used as starting points. These singlet-triplet crossings (STCs) accessible from the ^{3}MC minima are hereafter referred as $(^3MC_{ax\,1}/S_0)_{stc\text{-}mecp}$, $(^3MC_{ax\,2}/S_0)_{stc\text{-}mecp}$ $(^3MC_{eq\,1}/S_0)_{stc\text{-}mecp}$ and $(^3MC_{eq\,2}/S_0)_{stc\text{-}mecp}$ respectively. In agreement with recent works,[5–9] these points are almost degenerate with the corresponding ^{3}MC minimum, the largest difference being of only 0.07 eV for $(^3MC_{ax\,1}/S_0)_{stc\text{-}mecp}$ in complex **2** (see Table S5).

To locate the transition state (TS) connecting a T_1 minimum with a ^{3}MC state, the climbing-image nudged-elastic band (CI-NEB) method was employed to find the minimum energy path (MEP) between them. Once the CI converged, its geometry was used as the starting point for the TS calculation. To confirm that the TS connected the desired triple minima, the intrinsic reaction coordinate (IRC) was additionally computed.

For complex **1**, CI-NEB approach successfully connects the two desired minima in most cases. Nevertheless, exceptions were found in three paths: $^3MLCT/^3MC_{ax\,1}$, $^3LC_{ppy\,2}/^3MC_{ax\,1}$, and $^3LC_{ppy\,2}/^3MC_{ax\,2}$ which instead passed through intermediate states: $^3LC_{ppy\,1}$, $^3LC_{ppy\,1}$, and ^{3}MLCT states respectively. In addition, the IRC analysis indicated that most of the obtained TSs connect the $^3LC_{ppy\,2}$ state to various ^{3}MC minima, except for the $^3LC_{ppy\,1}/^3MC_{ax\,1}$ path. This indicates that non-radiative decay primarily involves the $^3LC_{ppy\,2}$ state, the lowest-energy T_1 state for complex **1** (which correspond to the **b** model presented in the main manuscript, see Figure 2). Notably, the IRC for the $^3MLCT/^3MC_{ax\,1}$ path incorrectly led to the $^3LC_{ppy\,1}$ state, likely because the initial CI-NEB path also connected these states.

For complex **2**, we were unable to optimize the TS connecting the ^{3}MLCT and $^3LC_{ppy}$ states with the $^3MC_{eq}$ state, which is expected to be the most relevant pathway. Consequently, the CI geometry instead of the TS geometry was used for the cases in which the TS was not localized. However as explained in the main text, since the CI-NEB is only 0.05 eV above the $^3MC_{eq\,1}$ minimum the true TS will not significantly change the barrier. Additionally, the CI-NEB calculation for the $^3LC_{ppy\,1}/^3MC_{ax\,1}$ path did not converge. In such a case, we used the CI-NEB geometry of $^3MLCT/^3MC_{ax\,1}$ path as a approximation. The relative energies of the lowest singlet (S_0) and triplet (T_1) states computed at the optimized geometries of all the critical points characterized are summarized in Table S5. Notice that in the Table S5 "T_1" refers to the lowest triplet state for a given geometry independently to its nature (^{3}MLCT, ^{3}LC or ^{3}MC). Figures S7 and S8 show the optimized geometries and the bond lengths of the Ir coordination sphere for the different critical points of complexes **1** and **2**, respectively. The coordination bond lengths of the Ir atom are also summarized in Table S1.

Table S5. Relative energy differences (versus $(S_0)_{min}$, in eV) computed at the DFT PBE0/DEF2-SVP level for the lowest singlet (S_0) and triplet (T_1) states at the optimized geometries of all the critical points localized for complex **1** and **2**.

Complex	Geometry	State	
		S_0	T_1
**1**	$(S_0)_{min}$	0.00	3.03
	$(^3MLCT)_{min}$	0.28	2.66
	$(^3LC_{ppy\,1})_{min}$	0.28	2.67
	$(^3LC_{ppy\,2})_{min}$	0.28	2.66
	$(^3MLCT/^3MC_{ax\,1})_{ts}$	1.35	3.29
	$(^3LC_{ppy\,1}/^3MC_{ax\,1})_{ts}$	1.34	3.30
	$(^3LC_{ppy\,2}/^3MC_{ax\,1})_{ts}$	1.34	3.30
	$(^3MC_{ax\,1})_{min}$	2.40	3.12
	$(^3MC_{ax\,1}/S_0)_{stc\text{-}mecp}$	3.16	3.16
	$(^3MLCT/^3MC_{ax\,2})_{ts}$	1.40	3.27
	$(^3LC_{ppy\,1}/^3MC_{ax\,2})_{ts}$	1.41	3.27
	$(^3LC_{ppy\,2}/^3MC_{ax\,2})_{ts}$	1.41	3.26
	$(^3MC_{ax\,2})_{min}$	2.58	3.11
	$(^3MC_{ax\,2}/S_0)_{stc\text{-}mecp}$	3.13	3.13
	$(^3MLCT/^3MC_{eq\,1})_{ts}$	2.44	3.19
	$^3LC_{ppy\,1}/^3MC_{eq\,1})_{ts}$	2.42	3.20
	$^3LC_{ppy\,2}/^3MC_{eq\,1})_{ts}$	2.44	3.20
	$(^3MC_{eq\,1})_{min}$	2.75	3.19
	$(^3MC_{eq\,1}/S_0)_{stc\text{-}mecp}$	3.21	3.21
	$(^3MLCT/^3MC_{eq\,2})_{ts}$	1.58	3.01
	$^3LC_{ppy\,1}/^3MC_{eq\,2})_{ts}$	1.57	3.01
	$^3LC_{ppy\,2}/^3MC_{eq\,2})_{ts}$	1.56	3.01
	$(^3MC_{eq\,2})_{min}$	2.50	3.00
	$(^3MC_{eq\,2}/S_0)_{stc\text{-}mecp}$	3.02	3.02
**2**	$(S_0)_{min}$	0.00	3.01
	$(^3MLCT)_{min}$	0.28	2.76
	$(^3LC_{ppy\,1})_{min}$	0.34	2.83
	$(^3MLCT/^3MC_{ax\,1})_{ts}$	1.27	3.37
	$(^3LC_{ppy\,1}/^3MC_{ax\,1})_{ts}$	----	----
	$(^3MC_{ax\,1})_{min}$	2.41	3.21
	$(^3MC_{ax\,1}/S_0)_{stc\text{-}mecp}$	3.28	3.28
	$(^3MLCT/^3MC_{eq\,1})_{ts}$	----	----
	$(^3LC_{ppy\,1}/^3MC_{eq\,1})_{ts}$	----	----
	$(^3MC_{eq\,1})_{min}$	3.08	3.27
	$(^3MC_{eq\,1}/S_0)_{stc\text{-}mecp}$	3.29	3.29

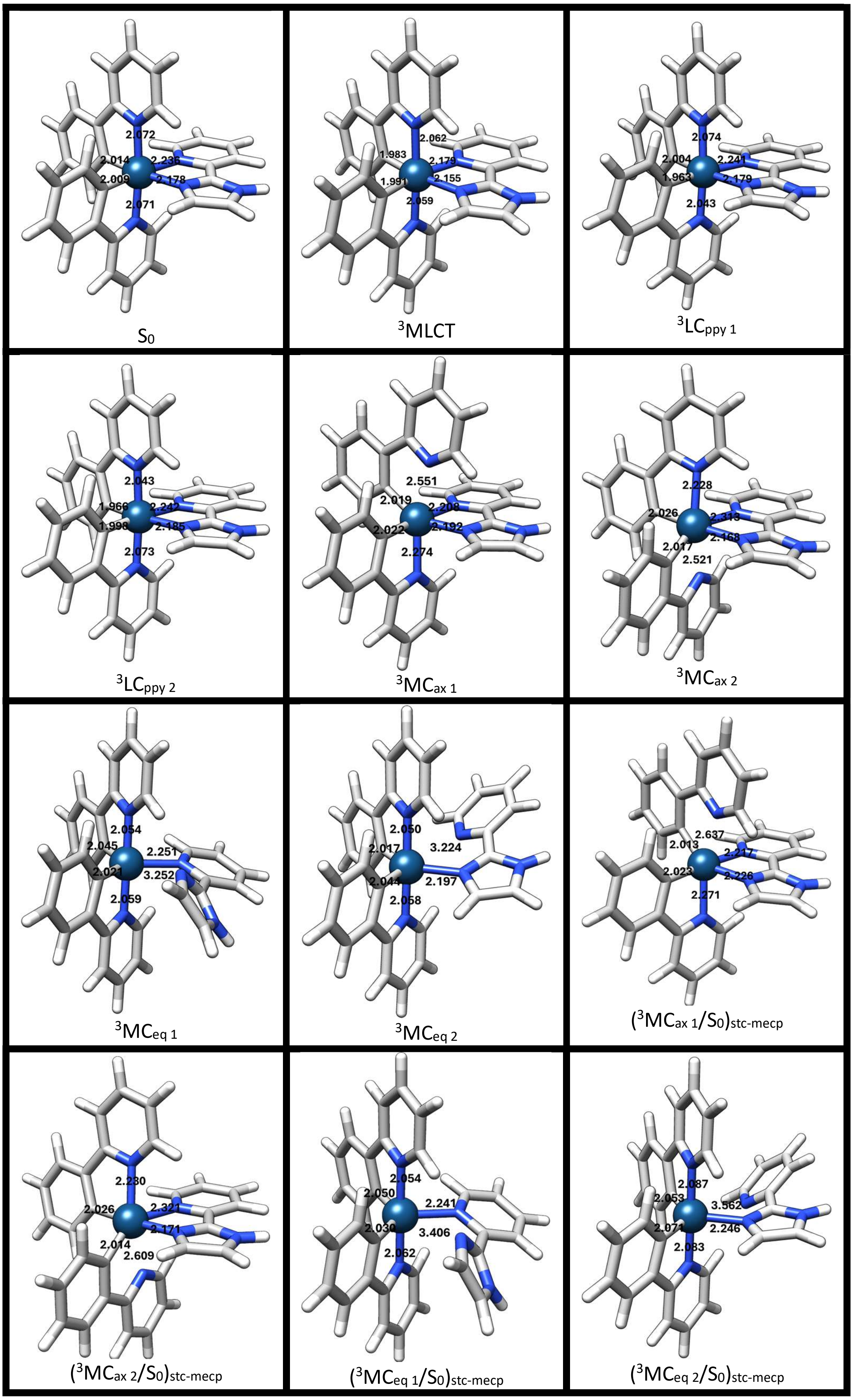
S0
3MLCT
3LCppy 1
3LCppy 2
3MCax 1
3MCax 2
3MCeq 1
3MCeq 2
(3MCax 1/S0)stc-mecp
(3MCax 2/S0)stc-mecp
(3MCeq 1/S0)stc-mecp
(3MCeq 2/S0)stc-mecp

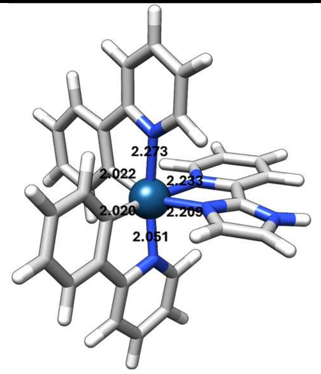

$(^{3}MLCT/^{3}MC_{ax\ 1})_{CI\text{-}NEB}$

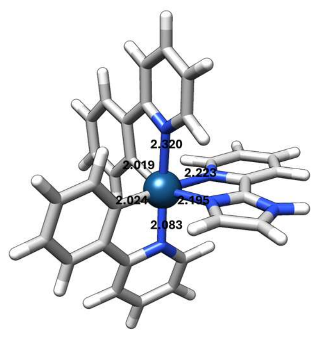

$(^{3}MLCT/^{3}MC_{ax\ 1})_{ts}$

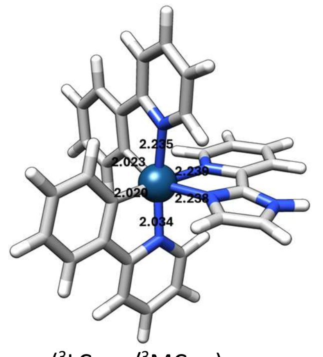

$(^{3}LC_{ppy\ 1}/^{3}MC_{ax\ 1})_{CI\text{-}NEB}$

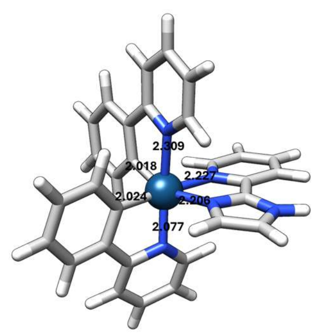

$(^{3}LC_{ppy\ 1}/^{3}MC_{ax\ 1})_{ts}$

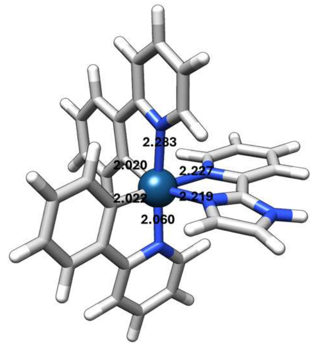

$(^{3}LC_{ppy\ 2}/^{3}MC_{ax\ 1})_{CI\text{-}NEB}$

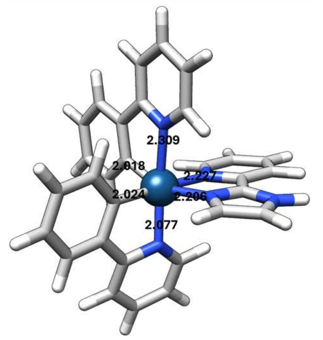

$(^{3}LC_{ppy\ 2}/^{3}MC_{ax\ 1})_{ts}$

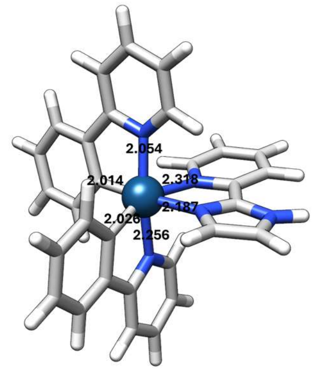

$(^{3}MLCT/^{3}MC_{ax\ 2})_{CI\text{-}NEB}$

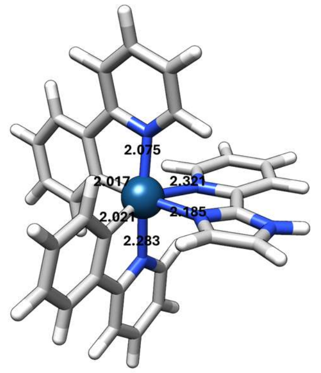

$(^{3}MLCT/^{3}MC_{ax\ 2})_{ts}$

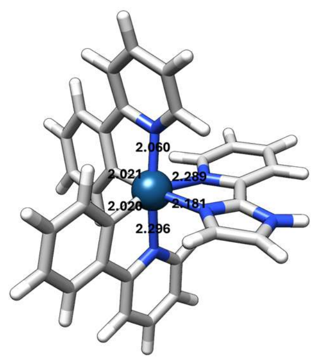

$(^{3}LC_{ppy\ 1}/^{3}MC_{ax\ 2})_{CI\text{-}NEB}$

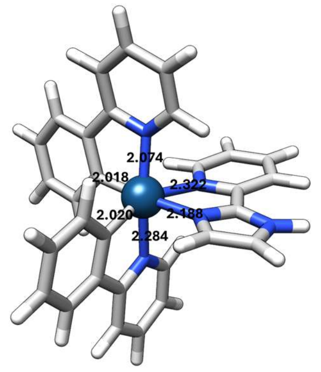

$(^{3}LC_{ppy\ 1}/^{3}MC_{ax\ 2})_{ts}$

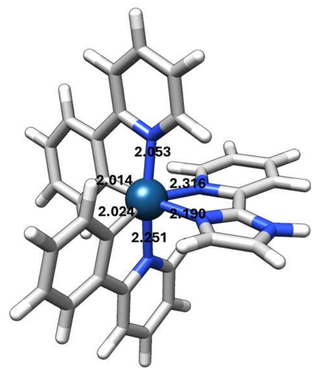

$(^{3}LC_{ppy\ 2}/^{3}MC_{ax\ 2})_{CI\text{-}NEB}$

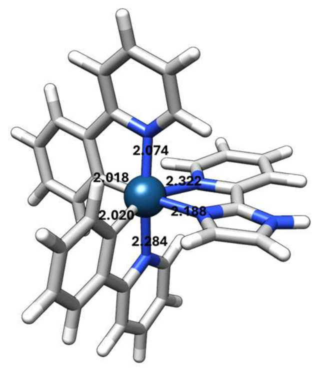

$(^{3}LC_{ppy\ 2}/^{3}MC_{ax\ 2})_{ts}$

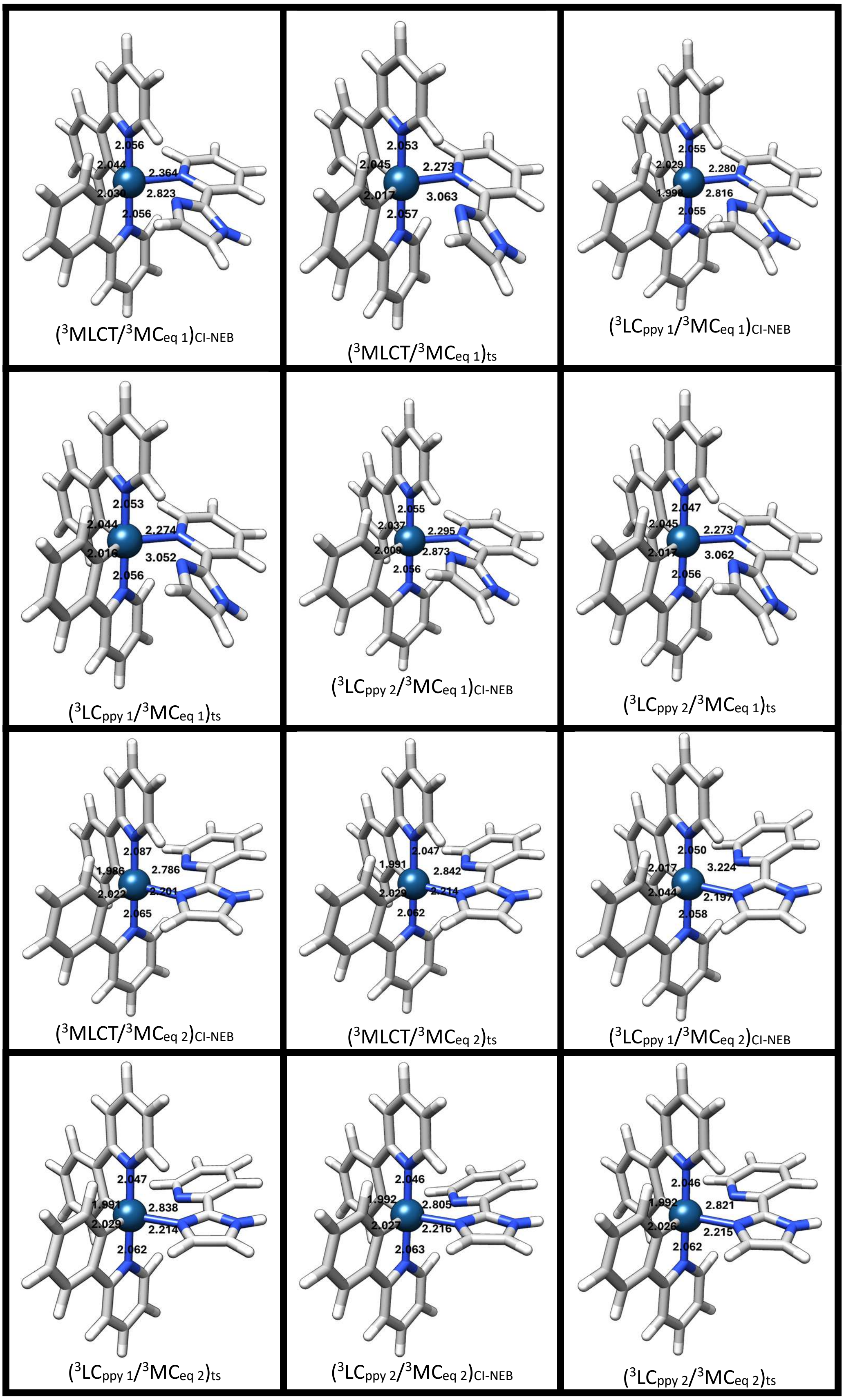

Figure S7. Geometries of complex **1.** The bond lengths (in Å) of the Ir coordination sphere are displayed.

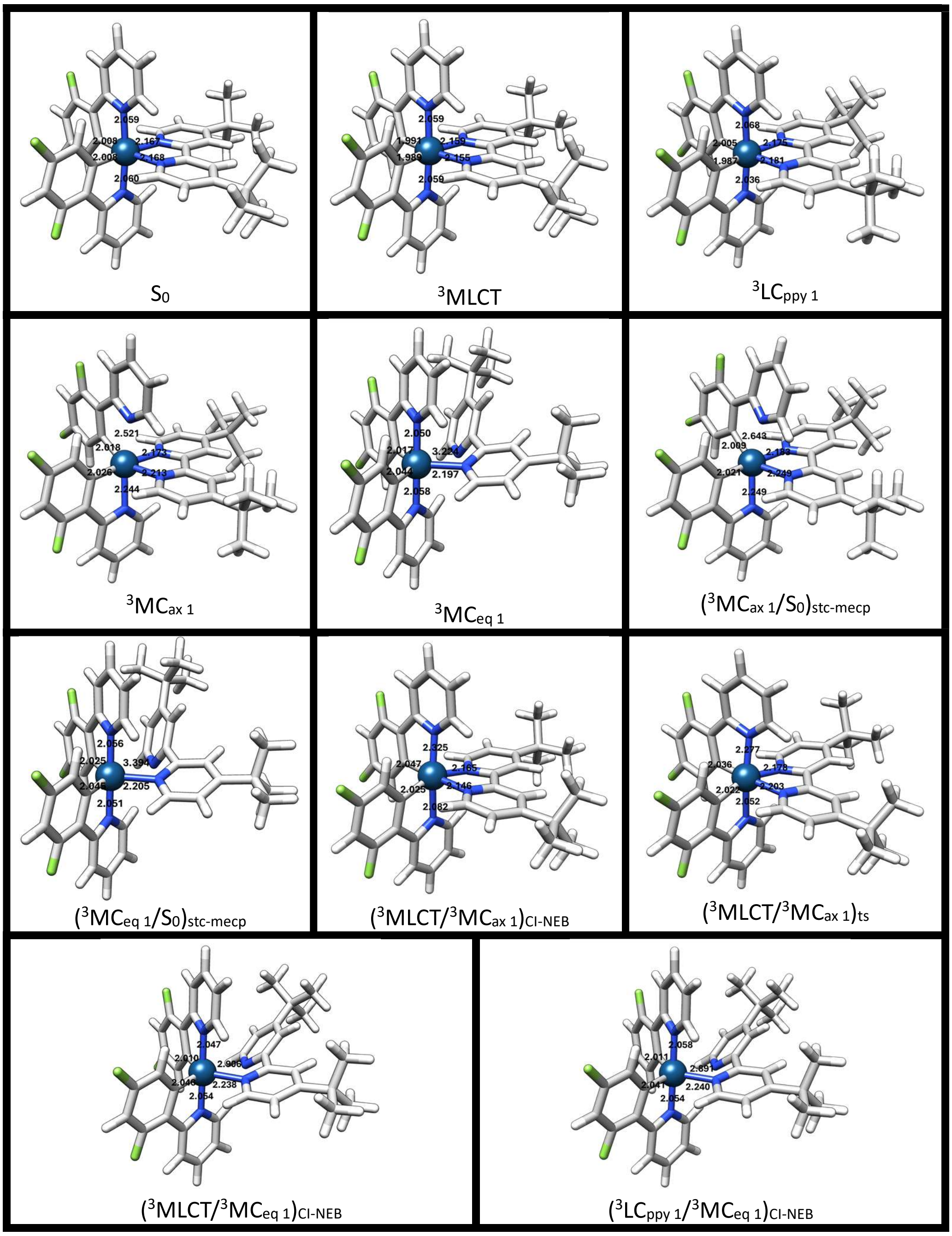

Figure S8. Geometries of complex **2.** The bond lengths (in Å) of the Ir coordination sphere are displayed.

Section S3. Computation of the radiative and non-radiative rate constants.

Once all the T_1 and ^{3}MC minima are well described and characterized, we are in the position to compute all the rate constants (k_{rad}, k_{ISC}, and $k_{nr}(T)$) that influence the emission quantum yield.Eqs. 1–5 are used to calculate the rate constants as explained in the "*Computational details*" section in the main text. Table S6 collects values estimated for the radiative (k_{rad}) and intersystem crossing (k_{ISC}) rate constants and the life-time (τ) for all the T_1 states of complexes **1** and **2**, together with the experimental data available.

Table S6. Radiative rate constants (k_{rad}), lifetimes (τ), and intersystem crossing rate constants (k_{ISC}) computed for the T_1 minima characterized for complexes **1** and **2**. The corresponding experimental values are also reported.

Complex	State	τ (μs)	k_{rad} (s^{-1})	k_{ISC} (s^{-1})
**1**	$(^3MLCT)_{min}$	1.03	9.69×10^{5}	4.94×10^{4}
	$(^3LC_{ppy\,1})_{min}$	10.93	9.15×10^{4}	1.65×10^{3}
	$(^3LC_{ppy\,2})_{min}$	10.04	9.96×10^{4}	1.28×10^{3}
	Experimental[a]	1.54	6.50×10^{5}	----
**2**	$(^3MLCT)_{min}$	2.38	4.20×10^{5}	2.22×10^{4}
	$(^3LC_{ppy\,1})_{min}$	3.71	2.70×10^{5}	1.09×10^{5}
	Experimental[b]	1.76	5.68×10^{5}	----

[a] Experimental data from reference [3]

[b] Experimental data from reference [4]

Table S7 and S8 summarizes all the ^{3}MC-mediated energies barriers (E_a, E_b, and E_c) and non-radiative decay rate constants (k_a, k_b, and k_c, see Figure 3 in the main text) that influence the calculation of $k_{nr}(T)$ computed for complexes **1** and **2** using NAST software.

Table S7. k_a, k_b, k_c, and $k_{nr}(T)$ rate constants (s^{-1}) computed for all possible ^{3}MC-mediated non-radiative decay paths ($T_1/^3MC$) of complexes **1** and **2**. The k_a and k_b values have been obtained using either the corresponding TS or CI-NEB structure.

Complex	Path	k_a (s^{-1})		k_b (s^{-1})		k_c (s^{-1})	$k_{nr}(T)$ (s^{-1})	
		TS	CI-NEB	TS	CI-NEB	MECP	TS	CI-NEB
**1**	$(^3MLCT)_{min}/(^3MC_{ax\,1})_{min}$	2.25×10^{2}	7.02×10^{1}	2.66×10^{10}	8.31×10^{9}	7.51×10^{11}	2.17×10^{2}	6.94×10^{1}
	$(^3MLCT)_{min}/(^3MC_{ax\,2})_{min}$	7.46×10^{2}	3.00×10^{2}	8.42×10^{10}	3.37×10^{10}	2.42×10^{12}	7.21×10^{2}	2.96×10^{2}
	$(^3MLCT)_{min}/(^3MC_{eq\,1})_{min}$	4.71×10^{3}	3.44×10^{3}	6.67×10^{12}[a]	6.67×10^{12}[a]	3.91×10^{12}	1.74×10^{3}	1.27×10^{3}
	$(^3MLCT)_{min}/(^3MC_{eq\,2})_{min}$	7.77×10^{6}	7.21×10^{6}	6.67×10^{12}[a]	6.67×10^{12}[a]	3.03×10^{12}	2.43×10^{6}	2.25×10^{6}
	$(^3LC_{ppy\,1})_{min}/(^3MC_{ax\,1})_{min}$	2.43×10^{2}	3.29×10^{1}	2.66×10^{10}	3.60×10^{9}	7.51×10^{11}	2.35×10^{2}	3.27×10^{1}
	$(^3LC_{ppy\,1})_{min}/(^3MC_{ax\,2})_{min}$	8.06×10^{2}	1.36×10^{2}	8.42×10^{10}	1.41×10^{10}	2.42×10^{12}	7.79×10^{2}	1.35×10^{2}
	$(^3LC_{ppy\,1})_{min}/(^3MC_{eq\,1})_{min}$	5.12×10^{3}	4.67×10^{3}	6.67×10^{12}[a]	6.67×10^{12}[a]	3.91×10^{12}	1.89×10^{3}	1.73×10^{3}
	$(^3LC_{ppy\,1})_{min}/(^3MC_{eq\,2})_{min}$	8.40×10^{6}	5.60×10^{6}	6.67×10^{12}[a]	6.67×10^{12}[a]	3.03×10^{12}	2.62×10^{6}	1.75×10^{6}
	$(^3LC_{ppy\,2})_{min}/(^3MC_{ax\,1})_{min}$	1.65×10^{2}	7.33×10^{1}	2.66×10^{10}	1.19×10^{10}	7.51×10^{11}	1.59×10^{2}	7.22×10^{1}
	$(^3LC_{ppy\,2})_{min}/(^3MC_{ax\,2})_{min}$	5.45×10^{2}	3.15×10^{2}	8.42×10^{10}	4.85×10^{10}	2.42×10^{12}	5.27×10^{2}	3.09×10^{2}
	$(^3LC_{ppy\,2})_{min}/(^3MC_{eq\,1})_{min}$	3.46×10^{3}	2.24×10^{3}	6.67×10^{12}[a]	6.67×10^{12}[a]	3.91×10^{12}	1.28×10^{3}	8.28×10^{2}
	$(^3LC_{ppy\,2})_{min}/(^3MC_{eq\,2})_{min}$	5.68×10^{6}	3.65×10^{6}	6.67×10^{12}[a]	6.67×10^{12}[a]	3.03×10^{12}	1.77×10^{6}	1.14×10^{6}
**2**	$(^3MLCT)_{min}/(^3MC_{ax\,1})_{min}$	1.18×10^{3}	4.94×10^{2}	7.47×10^{10}	3.12×10^{10}	3.86×10^{11}	9.89×10^{2}	4.57×10^{2}
	$(^3MLCT)_{min}/(^3MC_{eq\,1})_{min}$	2.08×10^{3}[b]	2.08×10^{3}	1.85×10^{12}[b]	1.85×10^{12}	3.39×10^{12}	1.35×10^{3}	1.35×10^{3}
	$(^3LC_{ppy\,1})_{min}/(^3MC_{ax\,1})_{min}$	1.53×10^{3}[c]	1.53×10^{3}[c]	3.12×10^{10}[c]	3.12×10^{10}[c]	3.86×10^{11}	1.42×10^{3}[c]	1.42×10^{3}[c]
	$(^3LC_{ppy\,1})_{min}/(^3MC_{eq\,1})_{min}$	1.51×10^{3}[b]	1.51×10^{3}	1.35×10^{12}[b]	1.35×10^{12}	3.39×10^{12}	1.08×10^{3}	1.08×10^{3}

[a]Using only the preexponential factor instead of k_b

[b]Using the CI-NEB structure instead of the TS.

[c]Using the CI-NEB corresponding to the $^3MLCT/^3MC_{ax\,1}$

Table S8. E_a, E_b, E_c, energies values (eV) computed for all possible ^{3}MC-mediated non-radiative decay paths (T_1/^{3}MC) of complexes **1** and **2**. The E_a and E_b values have been obtained using either the corresponding TS or CI-NEB structure.

Complex	Path	E_a (eV)		E_b (eV)		E_c (eV)
		TS	CI-NEB	TS	CI-NEB	MECP
1	$(^3MLCT)_{min}/(^3MC_{ax\ 1})_{min}$	0.63	0.67	0.18	0.21	0.04
	$(^3MLCT)_{min}/(^3MC_{ax\ 2})_{min}$	0.61	0.63	0.15	0.18	0.02
	$(^3MLCT)_{min}/(^3MC_{eq\ 1})_{min}$	0.53	0.55	0.00	0.00	0.02
	$(^3MLCT)_{min}/(^3MC_{eq\ 2})_{min}$	0.35	0.35	0.01	0.01	0.02
	$(^3LC_{ppy\ 1})_{min}/(^3MC_{ax\ 1})_{min}$	0.63	0.68	0.18	0.23	0.04
	$(^3LC_{ppy\ 1})_{min}/(^3MC_{ax\ 2})_{min}$	0.60	0.64	0.15	0.19	0.02
	$(^3LC_{ppy\ 1})_{min}/(^3MC_{eq\ 1})_{min}$	0.53	0.54	0.00	0.01	0.02
	$(^3LC_{ppy\ 1})_{min}/(^3MC_{eq\ 2})_{min}$	0.34	0.36	0.01	0.02	0.02
	$(^3LC_{ppy\ 2})_{min}/(^3MC_{ax\ 1})_{min}$	0.64	0.66	0.18	0.20	0.04
	$(^3LC_{ppy\ 2})_{min}/(^3MC_{ax\ 2})_{min}$	0.60	0.62	0.15	0.17	0.02
	$(^3LC_{ppy\ 2})_{min}/(^3MC_{eq\ 1})_{min}$	0.54	0.56	0.00	0.02	0.02
	$(^3LC_{ppy\ 2})_{min}/(^3MC_{eq\ 2})_{min}$	0.35	0.36	0.01	0.02	0.02
2	$(^3MLCT)_{min}/(^3MC_{ax\ 1})_{min}$	0.62	0.65	0.16	0.20	0.07
	$(^3MLCT)_{min}/(^3MC_{eq\ 1})_{min}$	----	0.56	----	0.04	----
	$(^3LC_{ppy\ 1})_{min}/(^3MC_{ax\ 1})_{min}$	----	----	----	----	----
	$(^3LC_{ppy\ 1})_{min}/(^3MC_{eq\ 1})_{min}$	----	0.50	----	0.06	----

Section S4: Additional data.

Table S9. Cartesian coordinates (Å) of the optimized geometries of complex **1**.

$(S_0)_{min}$ PBE0/DEF2-SVP CPCM (CH_3CN).

Ir	0.053022000	-0.003296000	-0.054306000
N	1.545567000	-1.095794000	0.876179000
C	2.815025000	-0.829949000	0.455828000
C	3.898812000	-1.524220000	1.015545000
C	3.678621000	-2.478925000	2.002135000
C	2.369732000	-2.732148000	2.422305000
C	1.334768000	-2.018714000	1.831056000
N	-1.328914000	1.251712000	-0.952723000
C	-1.588830000	2.411857000	-0.285258000
C	-2.523054000	3.323240000	-0.801447000
C	-3.178401000	3.046443000	-1.995968000
C	-2.891053000	1.855166000	-2.668388000
C	-1.960576000	0.987623000	-2.110262000
H	4.912206000	-1.310216000	0.675736000
H	4.520039000	-3.020021000	2.441655000
H	2.145566000	-3.468947000	3.195236000
H	-1.701548000	0.048572000	-2.600635000
H	-3.375098000	1.594624000	-3.610984000
H	-3.905260000	3.754444000	-2.401383000
H	-2.730242000	4.248601000	-0.263711000
C	1.662409000	0.777062000	-0.970147000
C	0.078126000	1.524670000	1.258065000
C	-0.818579000	2.586194000	0.950894000
C	-0.935999000	3.712193000	1.786712000
C	-0.163721000	3.806302000	2.942597000
C	0.727111000	2.772545000	3.261355000
C	0.845521000	1.652919000	2.431460000
H	-1.629089000	4.520204000	1.539830000
H	-0.253330000	4.680715000	3.592097000
H	1.337431000	2.842433000	4.166968000
C	2.905234000	0.206111000	-0.577929000
C	4.117607000	0.624186000	-1.157367000
C	4.118496000	1.616740000	-2.135054000
C	2.903980000	2.192079000	-2.532597000
C	1.697607000	1.779777000	-1.957766000
H	5.066301000	0.177484000	-0.849421000
H	5.059676000	1.941703000	-2.585598000
H	2.898393000	2.973132000	-3.299036000
H	0.770743000	2.253514000	-2.290983000
N	-0.245120000	-1.702194000	-1.384936000
H	1.553479000	0.867829000	2.709419000
N	-1.681393000	-1.115366000	0.813853000
C	-2.058596000	-2.231721000	0.141793000
C	-3.147070000	-3.008082000	0.553191000
C	-3.864402000	-2.614668000	1.681474000
C	-3.477448000	-1.460446000	2.364125000
C	-2.379031000	-0.741956000	1.894088000
H	-2.036952000	0.164589000	2.398188000
H	-4.718797000	-3.204545000	2.021084000
H	-4.011918000	-1.114186000	3.250327000
H	-3.432776000	-3.903344000	-0.000868000
C	-1.233960000	-2.520551000	-1.027087000
C	0.332900000	-2.229382000	-2.505247000
N	-1.306157000	-3.555571000	-1.894300000
C	-0.327845000	-3.393488000	-2.834929000
H	0.298819000	-2.181506000	2.130263000
H	-1.965334000	-4.327572000	-1.865510000
H	1.173265000	-1.753227000	-3.005052000
H	-0.182153000	-4.103669000	-3.644992000

$(^3MLCT)_{min}$ UPBE0/DEF2-SVP CPCM (CH_3CN).

Ir	-1.696729000	-2.411028000	0.606743000
N	-2.746059000	-1.927280000	-1.097324000
C	-2.877356000	-2.934731000	-1.997885000
C	-3.550714000	-2.713210000	-3.202046000

C	-4.079724000	-1.456663000	-3.471113000
C	-3.925349000	-0.436325000	-2.534007000
C	-3.247042000	-0.712808000	-1.354947000
N	-0.714372000	-2.973109000	2.329855000
C	-1.522079000	-3.275636000	3.378450000
C	-0.967824000	-3.629847000	4.610746000
C	0.413972000	-3.667243000	4.756316000
C	1.222513000	-3.348656000	3.666933000
C	0.616751000	-3.003952000	2.465979000
H	-3.656386000	-3.524132000	-3.923263000
H	-4.609478000	-1.274382000	-4.408947000
H	-4.321574000	0.565569000	-2.705555000
H	1.199582000	-2.747026000	1.579605000
H	2.311193000	-3.364085000	3.735890000
H	0.856849000	-3.943655000	5.715875000
H	-1.620156000	-3.872896000	5.449854000
C	-1.617683000	-4.176988000	-0.309470000
C	-3.276310000	-2.772477000	1.750598000
C	-2.952690000	-3.185426000	3.078888000
C	-3.959820000	-3.485518000	3.998920000
C	-5.297551000	-3.370689000	3.621091000
C	-5.635346000	-2.957390000	2.325780000
C	-4.638142000	-2.658516000	1.403732000
H	-3.715327000	-3.810227000	5.012838000
H	-6.084398000	-3.603373000	4.342626000
H	-6.686995000	-2.871266000	2.040496000
C	-2.263565000	-4.196784000	-1.580247000
C	-2.300019000	-5.368929000	-2.341075000
C	-1.680141000	-6.522632000	-1.863710000
C	-1.023604000	-6.513138000	-0.626537000
C	-0.985400000	-5.351211000	0.138677000
H	-2.807963000	-5.393217000	-3.307735000
H	-1.704739000	-7.436533000	-2.462056000
H	-0.539900000	-7.422780000	-0.261188000
H	-0.467112000	-5.361814000	1.100478000
N	0.070920000	-1.768132000	-0.445299000
H	-4.920170000	-2.336432000	0.398594000
N	-1.405484000	-0.352182000	1.259463000
C	-0.306471000	0.310241000	0.692913000
C	-0.014511000	1.648627000	1.055283000
C	-0.789496000	2.301871000	1.981328000
C	-1.892037000	1.603625000	2.572742000
C	-2.140278000	0.307633000	2.182014000
H	-2.979804000	-0.248199000	2.608208000
H	-0.565884000	3.332807000	2.264476000
H	-2.537250000	2.076103000	3.314831000
H	0.840821000	2.148653000	0.593127000
C	0.431139000	-0.486273000	-0.213892000
C	0.938342000	-2.281911000	-1.378680000
N	1.522027000	-0.198273000	-0.976849000
C	1.848035000	-1.317803000	-1.713298000
H	-3.095375000	0.044984000	-0.584123000
H	2.007111000	0.690297000	-1.010071000
H	0.860799000	-3.304807000	-1.740769000
H	2.691353000	-1.332718000	-2.398625000

$(^3LC_{ppy\ 1})_{min}$ UPBE0/DEF2-SVP CPCM (CH_3CN).

Ir	-1.620668000	-2.383202000	0.559242000
N	-2.684527000	-1.904181000	-1.117234000
C	-2.926745000	-2.997564000	-1.971336000
C	-3.691193000	-2.788335000	-3.161106000
C	-4.163860000	-1.542041000	-3.477103000
C	-3.882410000	-0.454667000	-2.601519000
C	-3.147860000	-0.697682000	-1.447610000
N	-0.668436000	-2.972418000	2.304680000
C	-1.498717000	-3.326107000	3.320776000
C	-0.966127000	-3.726857000	4.551102000
C	0.410375000	-3.759866000	4.729863000
C	1.243434000	-3.389715000	3.673996000
C	0.660579000	-3.001984000	2.476633000
H	-3.891543000	-3.639543000	-3.814234000

H	-4.748463000	-1.383015000	-4.385909000
H	-4.232683000	0.556627000	-2.811201000
H	1.267504000	-2.709983000	1.617683000
H	2.330325000	-3.401296000	3.767392000
H	0.832036000	-4.073720000	5.687630000
H	-1.635326000	-4.011480000	5.363499000
C	-1.629890000	-4.166629000	-0.261370000
C	-3.227635000	-2.801154000	1.682031000
C	-2.924809000	-3.236705000	2.998883000
C	-3.945434000	-3.549654000	3.908103000
C	-5.277725000	-3.431256000	3.524164000
C	-5.591963000	-2.997358000	2.232125000
C	-4.579529000	-2.685461000	1.324171000
H	-3.706492000	-3.886576000	4.919877000
H	-6.073210000	-3.675269000	4.232369000
H	-6.639026000	-2.903150000	1.930395000
C	-2.367075000	-4.209996000	-1.542544000
C	-2.466902000	-5.458587000	-2.238672000
C	-1.858356000	-6.576898000	-1.726458000
C	-1.128613000	-6.540291000	-0.497575000
C	-1.035759000	-5.345567000	0.211415000
H	-3.017846000	-5.521343000	-3.179474000
H	-1.932269000	-7.522739000	-2.271108000
H	-0.657048000	-7.450320000	-0.120282000
H	-0.490961000	-5.332716000	1.159014000
N	0.155031000	-1.658473000	-0.475019000
H	-4.849284000	-2.348922000	0.319825000
N	-1.349232000	-0.271135000	1.258305000
C	-0.287964000	0.388804000	0.741314000
C	0.018215000	1.697564000	1.116511000
C	-0.793477000	2.332396000	2.050738000
C	-1.882144000	1.645753000	2.583037000
C	-2.122465000	0.342955000	2.158181000
H	-2.966294000	-0.233488000	2.545133000
H	-0.573812000	3.356340000	2.361265000
H	-2.543521000	2.106049000	3.318771000
H	0.880744000	2.210346000	0.687352000
C	0.480404000	-0.395882000	-0.213527000
C	1.044377000	-2.126957000	-1.394985000
N	1.559709000	-0.045580000	-0.941970000
C	1.933381000	-1.119806000	-1.694548000
H	-2.930296000	0.113923000	-0.749415000
H	2.020402000	0.860113000	-0.945410000
H	1.000154000	-3.143089000	-1.780703000
H	2.784015000	-1.084115000	-2.370526000

$(^3LC_{ppy\ 2})_{min}$ UPBE0/DEF2-SVP CPCM (CH_3CN).

Ir	-1.776131000	-2.747853000	0.506266000
N	-2.837838000	-2.251525000	-1.166630000
C	-3.103581000	-3.339568000	-2.019719000
C	-3.863531000	-3.114521000	-3.208399000
C	-4.316102000	-1.859608000	-3.520837000
C	-4.018546000	-0.779467000	-2.641552000
C	-3.285367000	-1.036204000	-1.490817000
N	-0.818905000	-3.342753000	2.246191000
C	-1.645544000	-3.645042000	3.281287000
C	-1.110185000	-4.032238000	4.514701000
C	0.266845000	-4.103957000	4.677217000
C	1.096750000	-3.785898000	3.602078000
C	0.510415000	-3.409142000	2.402793000
H	-4.079183000	-3.960741000	-3.863227000
H	-4.898347000	-1.688954000	-4.429050000
H	-4.355406000	0.237168000	-2.847461000
H	1.116534000	-3.156344000	1.530872000
H	2.183924000	-3.828307000	3.682287000
H	0.691322000	-4.406601000	5.637335000
H	-1.777522000	-4.274674000	5.342274000
C	-1.843453000	-4.538610000	-0.302461000
C	-3.374968000	-3.100294000	1.651506000
C	-3.071043000	-3.517066000	2.973834000
C	-4.092211000	-3.780291000	3.897850000

C	-5.423903000	-3.631489000	3.522770000
C	-5.737503000	-3.217918000	2.223773000
C	-4.725744000	-2.954912000	1.300287000
H	-3.853435000	-4.103376000	4.914129000
H	-6.219636000	-3.837499000	4.242632000
H	-6.784136000	-3.101411000	1.928529000
C	-2.576078000	-4.566448000	-1.586200000
C	-2.706205000	-5.812511000	-2.280316000
C	-2.134983000	-6.947174000	-1.760459000
C	-1.414486000	-6.929236000	-0.525435000
C	-1.288911000	-5.735229000	0.178077000
H	-3.251254000	-5.862373000	-3.225374000
H	-2.232253000	-7.891778000	-2.303632000
H	-0.974697000	-7.852350000	-0.141550000
H	-0.749423000	-5.734141000	1.129104000
H	-4.994899000	-2.634606000	0.290755000
C	0.448255000	-0.809408000	-0.385561000
H	-3.052087000	-0.232293000	-0.788559000
N	-1.423692000	-0.689755000	1.150090000
C	-1.963623000	0.199321000	2.031001000
H	-2.849110000	-0.029992000	2.619630000
C	-1.208839000	1.350152000	2.008725000
H	-1.301699000	2.285765000	2.554357000
N	-0.211155000	1.133060000	1.104689000
H	0.517356000	1.799064000	0.863380000
C	-0.361463000	-0.108336000	0.600234000
N	0.048214000	-2.080427000	-0.612945000
C	0.709862000	-2.821987000	-1.505186000
H	0.349378000	-3.844073000	-1.646561000
C	1.804328000	-2.336071000	-2.213536000
H	2.312851000	-2.979953000	-2.932888000
C	2.224993000	-1.028856000	-1.980224000
H	3.081191000	-0.613886000	-2.516759000
C	1.539648000	-0.251312000	-1.052653000
H	1.845677000	0.776813000	-0.851894000

(^{3}MLCT/^{3}MC$_{ax\,1}$)$_{ts}$ UPBE0/DEF2-SVP CPCM (CH_3CN).

Ir	0.064489000	0.015628000	-0.260100000
N	0.794871000	-0.904145000	1.740888000
N	-0.257112000	0.631587000	-2.224188000
C	0.043517000	-1.808118000	3.818537000
C	-0.219607000	-1.222595000	2.573776000
C	2.056345000	-1.153294000	2.097806000
C	-1.561714000	-0.913771000	2.065222000
C	-1.629133000	-0.340069000	0.779845000
C	-2.882681000	-0.007754000	0.247155000
C	-4.048481000	-0.254990000	0.975339000
C	-3.977309000	-0.824244000	2.249107000
C	-2.738507000	-1.149528000	2.792359000
C	-0.870372000	-0.354296000	-2.978464000
C	-1.711307000	-0.015285000	-4.034765000
C	-1.306934000	2.314970000	-3.584360000
C	-0.477174000	1.938377000	-2.549871000
C	-0.589596000	-1.706928000	-2.504081000
C	-0.097308000	-1.781749000	-1.175738000
C	0.204902000	-3.046797000	-0.655797000
C	0.011248000	-4.202012000	-1.418111000
C	-0.487793000	-4.114714000	-2.721504000
C	-0.787119000	-2.868758000	-3.265482000
H	-4.889429000	-1.011544000	2.820573000
H	-2.692890000	-1.588382000	3.791788000
H	-2.961059000	0.443684000	-0.747260000
H	-5.019369000	0.003365000	0.543411000
H	0.585698000	-3.142958000	0.364312000
H	-1.158015000	-2.799231000	-4.291824000
H	0.246714000	-5.180079000	-0.988794000
H	-0.636238000	-5.019755000	-3.316097000
H	2.832623000	-0.886037000	1.378418000
C	1.356412000	-2.065488000	4.191716000
H	-0.778281000	-2.062125000	4.488755000
H	1.572102000	-2.524150000	5.159714000

C	2.388705000	-1.733530000	3.315953000
H	3.435172000	-1.920200000	3.562875000
C	-1.942034000	1.323566000	-4.352656000
H	0.065857000	2.679530000	-1.958949000
H	-1.435105000	3.376029000	-3.808161000
H	-2.604162000	1.592633000	-5.177997000
H	-2.195388000	-0.811301000	-4.604263000
C	1.606496000	2.527269000	0.601432000
C	1.922277000	3.770629000	1.151997000
C	0.910166000	4.514411000	1.748971000
C	-0.383505000	3.999286000	1.778298000
C	-0.618358000	2.750401000	1.212750000
N	0.348701000	2.028635000	0.640065000
H	2.943731000	4.152475000	1.116753000
H	1.133093000	5.489897000	2.186891000
H	-1.207893000	4.550194000	2.233835000
C	2.560958000	1.659354000	-0.073246000
N	3.859564000	1.871143000	-0.361381000
N	2.196037000	0.463788000	-0.531835000
H	-1.616750000	2.306267000	1.215765000
C	3.283970000	-0.099409000	-1.132088000
C	4.336360000	0.778841000	-1.028289000
H	3.249932000	-1.083960000	-1.593379000
H	5.366594000	0.716097000	-1.369922000
H	4.404090000	2.699144000	-0.135705000

($(^3LC_{ppy\ 1}/^3MC_{ax\ 1})_{ts}$ UPBE0/DEF2-SVP CPCM (CH_3CN).

Ir	-0.005254000	-0.014129000	-0.256885000
N	-0.696240000	0.420376000	-2.166284000
C	-1.272974000	-0.677723000	-2.781632000
C	-2.273758000	-0.507534000	-3.734587000
C	-2.701655000	0.771750000	-4.089617000
C	-2.100727000	1.878378000	-3.463879000
C	-1.111403000	1.669037000	-2.527586000
N	1.171327000	-0.675605000	1.617150000
C	0.370569000	-1.006206000	2.653931000
C	0.924271000	-1.395277000	3.879988000
C	2.304744000	-1.449624000	4.022846000
C	3.113160000	-1.116790000	2.937094000
C	2.498014000	-0.733844000	1.751374000
H	-2.724092000	-1.389474000	-4.194561000
H	-3.487781000	0.908082000	-4.834858000
H	-2.381860000	2.900839000	-3.724381000
H	3.092212000	-0.469195000	0.874774000
H	4.202145000	-1.151480000	2.998713000
H	2.746658000	-1.752697000	4.975078000
H	0.273621000	-1.653652000	4.715958000
C	-0.091948000	-1.879079000	-1.037405000
C	-1.433300000	-0.492397000	1.086896000
C	-1.068497000	-0.925576000	2.376803000
C	-2.059918000	-1.248514000	3.316211000
C	-3.405787000	-1.148529000	2.978706000
C	-3.771071000	-0.718826000	1.700450000
C	-2.792375000	-0.387254000	0.760905000
H	-1.784393000	-1.583001000	4.319230000
H	-4.172573000	-1.404336000	3.713675000
H	-4.827689000	-0.639323000	1.429895000
C	-0.768738000	-1.953247000	-2.281982000
C	-0.927303000	-3.180592000	-2.943905000
C	-0.409480000	-4.344086000	-2.382732000
C	0.267371000	-4.284035000	-1.160387000
C	0.423341000	-3.064119000	-0.496710000
H	-1.441380000	-3.224551000	-3.907976000
H	-0.526677000	-5.299333000	-2.900812000
H	0.673462000	-5.197653000	-0.716774000
H	0.946887000	-3.047044000	0.462886000
N	1.975114000	0.667991000	-0.942056000
H	-3.099589000	-0.050900000	-0.234742000
N	0.181231000	2.073197000	0.491350000
C	1.334776000	2.715788000	0.192394000
C	1.591839000	4.012810000	0.640606000

C	0.628375000	4.659478000	1.407002000
C	-0.560394000	3.996427000	1.702938000
C	-0.740102000	2.702292000	1.226077000
H	-1.654305000	2.142785000	1.438553000
H	0.807472000	5.673954000	1.770120000
H	-1.344399000	4.467001000	2.298325000
H	2.532883000	4.508832000	0.397893000
C	2.243719000	1.935286000	-0.634266000
C	2.981342000	0.214772000	-1.743832000
N	3.402747000	2.301202000	-1.216375000
C	3.885215000	1.235192000	-1.920698000
H	-0.594879000	2.505663000	-2.051929000
H	3.843675000	3.215822000	-1.170459000
H	2.999481000	-0.798733000	-2.138544000
H	4.814892000	1.287404000	-2.481967000

($^3LC_{ppy\ 2}$/$^3MC_{ax\ 1}$)$_{ts}$ UPBE0/DEF2-SVP CPCM (CH_3CN).

Ir	0.035738000	0.039366000	0.253876000
N	-1.196927000	0.660918000	-1.596540000
C	-1.460955000	-0.371918000	-2.426819000
C	-2.204063000	-0.167983000	-3.596101000
C	-2.678212000	1.102937000	-3.894681000
C	-2.402443000	2.153110000	-3.020657000
C	-1.654762000	1.881890000	-1.881243000
N	0.906181000	-0.168746000	2.127883000
C	0.107398000	-0.884580000	3.003924000
C	0.678920000	-1.622677000	4.036828000
C	2.063118000	-1.645826000	4.207823000
C	2.862036000	-0.909019000	3.315602000
C	2.258842000	-0.187444000	2.308086000
H	-2.409588000	-1.003374000	-4.266199000
H	-3.261398000	1.272015000	-4.803104000
H	-2.757843000	3.167431000	-3.209697000
H	2.845270000	0.427718000	1.622067000
H	3.948319000	-0.875081000	3.421147000
H	2.513784000	-2.225991000	5.015481000
H	0.029277000	-2.190403000	4.706047000
C	-0.203594000	-1.674331000	-0.784100000
C	-1.604993000	-0.380307000	1.361799000
C	-1.314413000	-0.819829000	2.678958000
C	-2.345082000	-1.171569000	3.564415000
C	-3.672419000	-1.086694000	3.154599000
C	-3.973248000	-0.648296000	1.861189000
C	-2.949473000	-0.298123000	0.976852000
H	-2.110833000	-1.497383000	4.581617000
H	-4.475469000	-1.355890000	3.845458000
H	-5.015595000	-0.583024000	1.536195000
C	-0.923734000	-1.667796000	-1.995250000
C	-1.087425000	-2.856899000	-2.721948000
C	-0.544981000	-4.048068000	-2.250439000
C	0.171247000	-4.058130000	-1.050864000
C	0.347195000	-2.878431000	-0.324284000
H	-1.642189000	-2.858074000	-3.663169000
H	-0.677944000	-4.970775000	-2.820099000
H	0.600105000	-4.991642000	-0.675952000
H	0.914409000	-2.907300000	0.611825000
H	-3.211826000	0.032276000	-0.031600000
C	2.189244000	1.920362000	-0.872495000
H	-1.421186000	2.673074000	-1.166622000
N	0.230998000	2.219049000	0.531464000
C	-0.371718000	3.214610000	1.243542000
H	-1.276791000	3.050376000	1.824358000
C	0.355738000	4.368754000	1.073728000
H	0.217793000	5.373923000	1.464369000
N	1.399274000	4.046602000	0.253915000
H	2.126392000	4.688904000	-0.048423000
C	1.304746000	2.739599000	-0.058205000
N	1.867606000	0.605830000	-0.878526000
C	2.630917000	-0.244068000	-1.570835000
H	2.333712000	-1.295171000	-1.542895000
C	3.745794000	0.169576000	-2.292485000

H	4.337736000	-0.565241000	-2.840410000
C	4.075716000	1.522988000	-2.297997000
H	4.940693000	1.884889000	-2.858210000
C	3.288077000	2.414095000	-1.577477000
H	3.522828000	3.479791000	-1.565722000

(^{3}MLCT/^{3}MC$_{ax\ 1}$)$_{ci\text{-}neb}$ UPBE0/DEF2-SVP CPCM (CH_3CN).

Ir	-0.091578274	-0.010070993	-0.191978746
N	0.687490391	-0.905530433	1.747033589
N	-0.318242766	0.647129770	-2.122033133
C	0.047262183	-1.688693893	3.910534166
C	-0.283213449	-1.185121565	2.646734817
C	1.968125603	-1.127893518	2.054983740
C	-1.647047267	-0.915669757	2.182450087
C	-1.762083937	-0.379768198	0.884722243
C	-3.034410223	-0.073312464	0.383487397
C	-4.171632664	-0.308113656	1.159859637
C	-4.053665890	-0.847588051	2.443751278
C	-2.795353130	-1.147987397	2.954663993
C	-0.680138567	-0.337513081	-3.030468503
C	-1.229113838	0.014082948	-4.261247030
C	-1.099106241	2.338257699	-3.641705985
C	-0.543684904	1.956750640	-2.441600982
C	-0.465543509	-1.690330218	-2.538105264
C	-0.213639636	-1.787370159	-1.144178516
C	-0.023809693	-3.061802199	-0.595432442
C	-0.073751226	-4.205340965	-1.397099800
C	-0.316354908	-4.096466669	-2.770652765
C	-0.513336613	-2.843259413	-3.340114915
H	-4.945199036	-1.033218321	3.047173936
H	-2.711086658	-1.564452920	3.961360366
H	-3.149745846	0.351038433	-0.618927673
H	-5.158963185	-0.066477347	0.756139206
H	0.163822368	-3.173746600	0.475550128
H	-0.690511272	-2.759550465	-4.415771592
H	0.076911620	-5.190252845	-0.946182461
H	-0.348031494	-4.991310064	-3.397368673
H	2.703355356	-0.898876064	1.282502132
C	1.379313989	-1.909547313	4.235004476
H	-0.739013204	-1.903674676	4.634829873
H	1.647833071	-2.299180040	5.219860582
C	2.363444672	-1.629391782	3.288395878
H	3.422655555	-1.793875066	3.493064973
C	-1.438356693	1.352793872	-4.589228171
H	-0.232599850	2.696257297	-1.701499838
H	-1.239255540	3.401013445	-3.850563617
H	-1.858302530	1.628103785	-5.558606361
H	-1.496758394	-0.775122009	-4.966821195
C	1.541289594	2.473509822	0.578084143
C	1.931853061	3.694414544	1.132621449
C	1.008942668	4.417100871	1.879358704
C	-0.275464843	3.903262713	2.051003384
C	-0.590708802	2.681144838	1.465624393
N	0.289957551	1.980094155	0.745803682
H	2.948240396	4.065337973	0.990214319
H	1.293730218	5.372179384	2.326604795
H	-1.029721683	4.436128447	2.632193115
C	2.414399704	1.624664441	-0.217200242
N	3.631684636	1.890484598	-0.734088444
N	2.048663610	0.392332158	-0.563355491
H	-1.585662618	2.242447701	1.574789369
C	3.046070255	-0.138696765	-1.327176768
C	4.047853392	0.795399276	-1.437035848
H	2.987714153	-1.143633215	-1.739710383
H	5.005839064	0.767778353	-1.950099752
H	4.148635272	2.761130957	-0.650145260

(^{3}LC$_{ppy\ 1}$/^{3}MC$_{ax\ 1}$)$_{ci\text{-}neb}$ UPBE0/DEF2-SVP CPCM (CH_3CN).

Ir	-0.135725391	-0.037745137	-0.184843121
N	-0.723042651	0.414773994	-2.079023389

C	-1.094241403	-0.673430985	-2.861365045
C	-1.776373405	-0.471045000	-4.058177900
C	-2.098656307	0.815457113	-4.487616165
C	-1.743284815	1.905424155	-3.665062467
C	-1.066900332	1.673964629	-2.490986470
N	1.105845254	-0.692805498	1.554704631
C	0.397027724	-1.024128437	2.659130155
C	1.051418063	-1.432961308	3.826827323
C	2.437759955	-1.510116196	3.844698672
C	3.149333664	-1.181782264	2.692128808
C	2.439448827	-0.778124465	1.569853568
H	-2.044416470	-1.336710676	-4.666814556
H	-2.609833784	0.974694660	-5.439267172
H	-1.964324689	2.934273747	-3.956681029
H	2.956887515	-0.515808200	0.647903612
H	4.238402168	-1.235153776	2.649052352
H	2.959983602	-1.829036488	4.749495246
H	0.471727196	-1.692233684	4.713856607
C	-0.224205258	-1.900499674	-0.960267791
C	-1.501105456	-0.493150675	1.236643292
C	-1.053728121	-0.930113725	2.499063758
C	-1.973551645	-1.243024090	3.510279507
C	-3.338468234	-1.129680476	3.268963442
C	-3.788048227	-0.698752972	2.017887515
C	-2.877752731	-0.375031396	1.008124761
H	-1.625436906	-1.578769046	4.490221740
H	-4.053201579	-1.375593113	4.057983608
H	-4.860654935	-0.610762925	1.825171888
C	-0.713660356	-1.956366741	-2.292029052
C	-0.833157259	-3.188272467	-2.958342956
C	-0.482480319	-4.368141794	-2.311196989
C	-0.009630142	-4.326065826	-0.995238424
C	0.119250146	-3.103597076	-0.331120041
H	-1.199460084	-3.224989802	-3.987768454
H	-0.577591919	-5.324153978	-2.832359006
H	0.255547048	-5.253037539	-0.479713911
H	0.480105469	-3.098425492	0.700494149
N	1.839096854	0.690292983	-0.944812511
H	-3.252919900	-0.036120841	0.037782908
N	0.068178639	2.064017896	0.558218243
C	1.203302657	2.721050745	0.218775607
C	1.461852334	4.020602616	0.661389579
C	0.521974636	4.654011092	1.465833076
C	-0.648859035	3.977143720	1.802284658
C	-0.831675247	2.682827928	1.327866983
H	-1.732867389	2.114737562	1.570090315
H	0.703322423	5.669181564	1.825903139
H	-1.415886143	4.437036254	2.427133213
H	2.387502840	4.526677291	0.382437221
C	2.087657302	1.963224221	-0.651426481
C	2.799580155	0.270110429	-1.817120626
N	3.200211428	2.363873687	-1.302031327
C	3.664723972	1.314477365	-2.041906957
H	-0.743239353	2.498066826	-1.852400346
H	3.617835834	3.289765922	-1.277981318
H	2.818139817	-0.740964988	-2.218209279
H	4.558263965	1.390910352	-2.656436794

$(^3LC_{ppy\ 2}/^3MC_{ax\ 1})_{ci\text{-}neb}$ UPBE0/DEF2-SVP CPCM (CH_3CN).

Ir	0.042944748	-0.035674271	0.198542525
N	-1.163054695	0.586523542	-1.636977756
C	-1.433286755	-0.438290089	-2.476978679
C	-2.170804159	-0.217499831	-3.646450243
C	-2.629562924	1.060793136	-3.937062141
C	-2.343848642	2.102092411	-3.055369834
C	-1.604517701	1.815524382	-1.914569379
N	0.915392255	-0.185867674	2.058992070
C	0.099162306	-0.764504823	3.018642383
C	0.656283243	-1.369326797	4.142391327
C	2.039208860	-1.405813756	4.315492141
C	2.855412353	-0.822916010	3.328239585

C	2.269541774	-0.222506018	2.236147487
H	-2.385994438	-1.046507227	-4.321595363
H	-3.209301786	1.242583272	-4.845294773
H	-2.686455663	3.121624441	-3.239596838
H	2.870551269	0.276748980	1.473130192
H	3.942933568	-0.811369295	3.425035923
H	2.476730614	-1.881188861	5.195531242
H	-0.005715778	-1.822131544	4.883333477
C	-0.218947049	-1.758978885	-0.822768751
C	-1.593483576	-0.429254535	1.319255042
C	-1.316845853	-0.719564906	2.680135843
C	-2.359579687	-0.962296009	3.589617627
C	-3.681098275	-0.930044416	3.156915831
C	-3.966119389	-0.658752998	1.814418673
C	-2.932174221	-0.409860404	0.908265901
H	-2.137028062	-1.161217043	4.641530902
H	-4.492611279	-1.111863561	3.866157817
H	-5.003912168	-0.641909720	1.469516385
C	-0.911746574	-1.740823454	-2.049392486
C	-1.067987159	-2.923793644	-2.788083565
C	-0.544410101	-4.121176451	-2.311766489
C	0.139157468	-4.143843966	-1.093094350
C	0.302491706	-2.971419728	-0.351765509
H	-1.600083431	-2.914320165	-3.742352821
H	-0.668896968	-5.039513234	-2.890383447
H	0.549871634	-5.083420378	-0.712873216
H	0.840585220	-3.011910325	0.600712319
H	-3.182554131	-0.204133912	-0.135752160
C	2.173328561	1.910168195	-0.842136060
H	-1.360177342	2.596101096	-1.192087576
N	0.196419188	2.149348179	0.550920962
C	-0.351602618	3.072441196	1.393090436
H	-1.283976713	2.894243418	1.924483924
C	0.463683174	4.178700831	1.412505521
H	0.389074088	5.121030628	1.949559576
N	1.505765913	3.902304989	0.573668307
H	2.296947150	4.516227475	0.401385117
C	1.324229307	2.666186807	0.066485211
N	1.841487987	0.601320135	-0.949764396
C	2.555844884	-0.184255430	-1.760366973
H	2.250159160	-1.232135929	-1.812305162
C	3.630266301	0.290309191	-2.505654341
H	4.179278481	-0.390710025	-3.157790132
C	3.974545042	1.636719146	-2.401841412
H	4.806661434	2.046737580	-2.978558612
C	3.239138076	2.460221139	-1.557084511
H	3.478681374	3.520845144	-1.464386772

$(^3MC_{ax\ 1})_{min}$ UPBE0/DEF2-SVP CPCM (CH_3CN).

Ir	0.148048000	-0.225613000	0.227361000
N	-1.252133000	0.737141000	-1.669301000
C	-1.543747000	-0.230135000	-2.555522000
C	-2.301748000	0.064550000	-3.700111000
C	-2.750308000	1.363178000	-3.907239000
C	-2.440742000	2.349307000	-2.971540000
C	-1.684568000	1.980159000	-1.863342000
N	1.073505000	-0.300948000	2.296469000
C	0.193328000	-0.698533000	3.241056000
C	0.640450000	-0.937140000	4.548182000
C	1.983637000	-0.761973000	4.857131000
C	2.872072000	-0.351705000	3.861609000
C	2.366229000	-0.133159000	2.587305000
H	-2.540222000	-0.715350000	-4.423580000
H	-3.340967000	1.603277000	-4.794803000
H	-2.775672000	3.381359000	-3.090993000
H	3.013855000	0.192508000	1.767942000
H	3.933706000	-0.201621000	4.064611000
H	2.338456000	-0.948001000	5.873893000
H	-0.059986000	-1.264497000	5.317052000
C	-0.287828000	-1.736336000	-1.038967000
C	-1.440256000	-0.688608000	1.390951000

C	-1.195657000	-0.855028000	2.780843000
C	-2.244297000	-1.153068000	3.666814000
C	-3.545764000	-1.292989000	3.197646000
C	-3.804074000	-1.132939000	1.833424000
C	-2.765807000	-0.836194000	0.950272000
H	-2.048739000	-1.270353000	4.735567000
H	-4.356838000	-1.523894000	3.892601000
H	-4.824475000	-1.242529000	1.454524000
C	-1.028575000	-1.573318000	-2.230821000
C	-1.254561000	-2.679621000	-3.067673000
C	-0.763714000	-3.938638000	-2.739980000
C	-0.032082000	-4.106990000	-1.562578000
C	0.202367000	-3.015818000	-0.724905000
H	-1.822939000	-2.561618000	-3.992890000
H	-0.950502000	-4.787232000	-3.402384000
H	0.357756000	-5.092248000	-1.291830000
H	0.777191000	-3.172148000	0.194369000
H	-2.994905000	-0.718539000	-0.112147000
C	2.233240000	1.712264000	-0.899253000
H	-1.421287000	2.717316000	-1.098961000
N	0.335336000	1.937997000	0.596292000
C	-0.278865000	2.912366000	1.325720000
H	-1.149881000	2.714369000	1.946647000
C	0.390843000	4.096039000	1.119030000
H	0.227197000	5.097360000	1.509468000
N	1.411307000	3.811347000	0.257793000
H	2.091086000	4.483376000	-0.086848000
C	1.357541000	2.499133000	-0.043045000
N	1.917914000	0.396896000	-0.940502000
C	2.669327000	-0.424549000	-1.679414000
H	2.375983000	-1.477111000	-1.681740000
C	3.764257000	0.019873000	-2.413474000
H	4.346642000	-0.692625000	-2.999877000
C	4.087546000	1.374154000	-2.381415000
H	4.937768000	1.759728000	-2.948369000
C	3.312086000	2.235772000	-1.612756000
H	3.542466000	3.301603000	-1.568312000

$(^{3}MC_{ax\,1}/S_0)_{stc\text{-}mecp}$ UPBE0/DEF2-SVP CPCM (CH_3CN).

Ir	0.375391000	-0.182280000	-0.106233000
N	-0.157488000	-0.994417000	2.345865000
N	1.868822000	0.523370000	-1.664611000
C	-1.873863000	-2.138632000	3.541092000
C	-1.354773000	-1.602561000	2.351250000
C	0.549039000	-0.899171000	3.468482000
C	-2.045504000	-1.668568000	1.050207000
C	-1.413052000	-1.105006000	-0.079992000
C	-2.070341000	-1.174042000	-1.320538000
C	-3.316054000	-1.789453000	-1.450103000
C	-3.937895000	-2.341424000	-0.328823000
C	-3.305263000	-2.276081000	0.907630000
C	2.583044000	-0.494577000	-2.192069000
C	3.450979000	-0.250595000	-3.265596000
C	2.815569000	2.070290000	-3.214472000
C	1.974928000	1.761588000	-2.153456000
C	2.362758000	-1.808384000	-1.565715000
C	1.360352000	-1.889272000	-0.561410000
C	1.156171000	-3.140733000	0.042937000
C	1.902844000	-4.261954000	-0.320240000
C	2.884645000	-4.164591000	-1.309690000
C	3.111153000	-2.940208000	-1.928901000
H	-4.915805000	-2.820686000	-0.417020000
H	-3.806204000	-2.708618000	1.776387000
H	-1.602868000	-0.743582000	-2.213864000
H	-3.800373000	-1.834303000	-2.429406000
H	0.393113000	-3.243395000	0.818590000
H	3.886250000	-2.869099000	-2.695924000
H	1.717118000	-5.220973000	0.172193000
H	3.473387000	-5.039693000	-1.595476000
H	1.518800000	-0.397593000	3.398381000
C	-1.136821000	-2.038392000	4.714754000

H	-2.845457000	-2.633156000	3.551373000
H	-1.530408000	-2.453474000	5.646021000
C	0.105454000	-1.406800000	4.686357000
H	0.721301000	-1.307839000	5.582236000
C	3.565534000	1.036242000	-3.776971000
H	1.365696000	2.531973000	-1.671670000
H	2.878062000	3.094140000	-3.586707000
H	4.238272000	1.232359000	-4.615568000
H	4.027984000	-1.065920000	-3.702641000
C	0.132495000	2.604192000	1.172274000
C	-0.364424000	3.853038000	1.547397000
C	-1.613302000	4.246639000	1.079686000
C	-2.326923000	3.383443000	0.252091000
C	-1.766622000	2.152406000	-0.072657000
N	-0.567613000	1.765840000	0.373676000
H	0.219731000	4.508955000	2.194939000
H	-2.022398000	5.220102000	1.358953000
H	-3.308845000	3.650732000	-0.141732000
C	1.433920000	2.088442000	1.570756000
N	2.402488000	2.681206000	2.298224000
N	1.838495000	0.882091000	1.190458000
H	-2.297158000	1.442913000	-0.712119000
C	3.097175000	0.696891000	1.678529000
C	3.461280000	1.823692000	2.379530000
H	3.661869000	-0.216600000	1.504978000
H	4.370698000	2.078316000	2.918190000
H	2.367542000	3.607926000	2.714194000

$(^{3}MLCT/^{3}MC_{ax\,2})_{ts}$ UPBE0/DEF2-SVP CPCM (CH_3CN).

Ir	-0.038800000	0.046069000	-0.222434000
N	-1.078880000	-0.779386000	1.634164000
N	1.384953000	0.677460000	-1.593064000
C	-3.057186000	-1.856220000	2.431424000
C	-2.309726000	-1.283669000	1.395239000
C	-0.565595000	-0.833278000	2.865058000
C	-2.755680000	-1.174162000	0.003088000
C	-1.854853000	-0.548197000	-0.881231000
C	-2.228891000	-0.359114000	-2.218320000
C	-3.461566000	-0.829275000	-2.677182000
C	-4.346467000	-1.461351000	-1.799225000
C	-3.998900000	-1.626934000	-0.461313000
C	1.962575000	-0.355531000	-2.302115000
C	2.576176000	-0.110445000	-3.529110000
C	2.038890000	2.223727000	-3.310105000
C	1.436194000	1.936382000	-2.102627000
C	1.817525000	-1.660299000	-1.662607000
C	0.883988000	-1.709229000	-0.591743000
C	0.704796000	-2.937919000	0.063402000
C	1.414496000	-4.074923000	-0.326050000
C	2.327160000	-4.010241000	-1.384678000
C	2.527512000	-2.806528000	-2.052833000
H	-5.314801000	-1.818501000	-2.157776000
H	-4.703689000	-2.110659000	0.219274000
H	-1.553655000	0.146234000	-2.916128000
H	-3.736448000	-0.692086000	-3.726670000
H	-0.007615000	-3.019940000	0.888072000
H	3.250987000	-2.756805000	-2.871052000
H	1.253211000	-5.021403000	0.197677000
H	2.886196000	-4.900194000	-1.684746000
H	0.431782000	-0.413147000	2.999823000
C	-2.521698000	-1.915018000	3.711518000
H	-4.053225000	-2.252710000	2.231109000
H	-3.093857000	-2.363399000	4.527240000
C	-1.248168000	-1.395211000	3.937215000
H	-0.783291000	-1.420293000	4.924168000
C	2.624660000	1.181928000	-4.046787000
H	0.987392000	2.723494000	-1.493575000
H	2.066394000	3.256584000	-3.662554000
H	3.105421000	1.376741000	-5.007600000
H	3.012675000	-0.943826000	-4.082611000
C	3.450961000	1.295477000	2.449265000

C	-0.218775000	2.779406000	1.070649000
N	-0.885517000	3.936034000	1.261700000
N	-0.884164000	2.014731000	0.207418000
C	1.056347000	2.346630000	1.618720000
C	1.929515000	3.155556000	2.351292000
C	3.138388000	2.616364000	2.775894000
C	2.531729000	0.556532000	1.714465000
N	1.361081000	1.066417000	1.322278000
H	1.671213000	4.191490000	2.577407000
H	3.837524000	3.226641000	3.351926000
H	4.390811000	0.837719000	2.762223000
H	2.726603000	-0.482575000	1.433992000
C	-2.004645000	2.699606000	-0.158642000
C	-2.015565000	3.908300000	0.497869000
H	-0.611410000	4.697223000	1.876328000
H	-2.728868000	2.294107000	-0.861481000
H	-2.725224000	4.731618000	0.484361000

($^{3}LC_{ppy\ 1}$/$^{3}MC_{ax\ 2}$)$_{ts}$ UPBE0/DEF2-SVP CPCM (CH_3CN).

Ir	0.119532000	-0.177856000	0.085660000
N	-1.120021000	0.459630000	-1.723192000
C	-1.329981000	-0.529418000	-2.620019000
C	-2.094182000	-0.296469000	-3.769776000
C	-2.649611000	0.958968000	-3.980475000
C	-2.427462000	1.964582000	-3.041180000
C	-1.651435000	1.666885000	-1.927536000
N	0.958522000	-0.304903000	1.978247000
C	0.153815000	-0.944482000	2.898123000
C	0.707100000	-1.504508000	4.048161000
C	2.077119000	-1.416723000	4.285509000
C	2.882269000	-0.758871000	3.341986000
C	2.294158000	-0.221146000	2.215718000
H	-2.250035000	-1.096270000	-4.494632000
H	-3.251346000	1.151448000	-4.871961000
H	-2.844559000	2.965861000	-3.160911000
H	2.884703000	0.314338000	1.469664000
H	3.958244000	-0.647958000	3.489271000
H	2.513152000	-1.855187000	5.185449000
H	0.056405000	-2.019423000	4.757387000
C	0.056965000	-1.819713000	-1.089916000
C	-1.523525000	-0.643199000	1.160524000
C	-1.253403000	-0.994794000	2.511261000
C	-2.290800000	-1.366844000	3.380324000
C	-3.605286000	-1.390211000	2.925230000
C	-3.889080000	-1.042980000	1.599741000
C	-2.860233000	-0.673581000	0.732149000
H	-2.074286000	-1.625694000	4.420125000
H	-4.412820000	-1.674197000	3.604792000
H	-4.921567000	-1.064011000	1.239667000
C	-0.699359000	-1.807908000	-2.278793000
C	-0.790998000	-2.964517000	-3.065898000
C	-0.133696000	-4.128303000	-2.675803000
C	0.631675000	-4.139620000	-1.506511000
C	0.741184000	-2.987021000	-0.725333000
H	-1.375851000	-2.964558000	-3.988882000
H	-0.210711000	-5.028247000	-3.290549000
H	1.155428000	-5.050469000	-1.203451000
H	1.355219000	-3.009017000	0.180653000
N	1.935513000	0.441351000	-0.966622000
H	-3.109417000	-0.415375000	-0.300162000
N	0.350193000	2.109301000	0.411976000
C	1.482685000	2.650371000	-0.081495000
C	1.860750000	3.961081000	0.221527000
C	1.041311000	4.714983000	1.053526000
C	-0.120119000	4.139848000	1.572607000
C	-0.425101000	2.828510000	1.227820000
H	-1.324096000	2.332876000	1.605333000
H	1.312344000	5.743205000	1.303428000
H	-0.787346000	4.697742000	2.231627000
H	2.785167000	4.378965000	-0.181347000
C	2.256916000	1.731336000	-0.898813000

C	2.864522000	-0.164178000	-1.759837000
N	3.368185000	1.963572000	-1.626309000
C	3.769305000	0.783006000	-2.180186000
H	-1.451666000	2.425940000	-1.170338000
H	3.826344000	2.861068000	-1.758063000
H	2.832982000	-1.228594000	-1.981017000
H	4.646283000	0.711379000	-2.818476000

($^3LC_{ppy\ 2}$/$^3MC_{ax\ 2}$)$_{ts}$ UPBE0/DEF2-SVP CPCM (CH_3CN).

Ir	0.119903000	-0.177885000	0.085564000
N	-1.121255000	0.455357000	-1.723774000
C	-1.331373000	-0.535651000	-2.618395000
C	-2.096568000	-0.305492000	-3.768053000
C	-2.652759000	0.949231000	-3.980976000
C	-2.430332000	1.956932000	-3.043985000
C	-1.653367000	1.661931000	-1.930277000
N	0.959915000	-0.300110000	1.977996000
C	0.156320000	-0.938479000	2.899646000
C	0.710704000	-1.495194000	4.050770000
C	2.080694000	-1.405174000	4.287396000
C	2.884699000	-0.748532000	3.342064000
C	2.295522000	-0.214185000	2.214742000
H	-2.252541000	-1.106909000	-4.491089000
H	-3.255265000	1.139550000	-4.872407000
H	-2.847957000	2.957772000	-3.165532000
H	2.885134000	0.320261000	1.467201000
H	3.960588000	-0.635948000	3.488700000
H	2.517594000	-1.841025000	5.188185000
H	0.060890000	-2.009239000	4.761436000
C	0.057668000	-1.822019000	-1.086880000
C	-1.522162000	-0.642632000	1.162206000
C	-1.251010000	-0.991126000	2.513544000
C	-2.287577000	-1.362326000	3.383953000
C	-3.602283000	-1.387837000	2.929627000
C	-3.887106000	-1.043618000	1.603573000
C	-2.859074000	-0.675104000	0.734638000
H	-2.070254000	-1.618764000	4.424182000
H	-4.409195000	-1.671107000	3.610221000
H	-4.919773000	-1.066310000	1.244117000
C	-0.699714000	-1.813076000	-2.275104000
C	-0.791310000	-2.971314000	-3.059818000
C	-0.132867000	-4.133881000	-2.668013000
C	0.633591000	-4.142356000	-1.499410000
C	0.742996000	-2.988143000	-0.720597000
H	-1.376992000	-2.973584000	-3.982275000
H	-0.209836000	-5.035097000	-3.280899000
H	1.158251000	-5.052244000	-1.195035000
H	1.357843000	-3.007905000	0.184891000
N	1.934675000	0.441081000	-0.968938000
H	-3.109090000	-0.419275000	-0.298063000
N	0.348701000	2.110056000	0.407701000
C	1.480744000	2.651102000	-0.086851000
C	1.858129000	3.962457000	0.214241000
C	1.038479000	4.717049000	1.045402000
C	-0.122476000	4.141954000	1.565616000
C	-0.426781000	2.829954000	1.222774000
H	-1.325376000	2.334319000	1.601246000
H	1.308979000	5.745779000	1.293785000
H	-0.789853000	4.700390000	2.224026000
H	2.782213000	4.380297000	-0.189441000
C	2.255242000	1.731384000	-0.903144000
C	2.863716000	-0.164966000	-1.761730000
N	3.366021000	1.963310000	-1.631491000
C	3.767667000	0.782217000	-2.183864000
H	-1.453414000	2.422673000	-1.174823000
H	3.823535000	2.860915000	-1.764729000
H	2.832766000	-1.229719000	-1.981373000
H	4.644382000	0.710257000	-2.822476000

(^3MLCT/$^3MC_{ax\ 2}$)$_{ci\text{-}neb}$ UPBE0/DEF2-SVP CPCM (CH_3CN).

Ir	-0.097965838	0.026357692	-0.298932519
N	-1.079018887	-0.754514443	1.576014582
N	1.320287570	0.678908047	-1.634124062
C	-3.065449266	-1.718906418	2.487524373
C	-2.333787438	-1.227250713	1.400091372
C	-0.527089055	-0.769190050	2.791659663
C	-2.815647425	-1.170800585	0.018224564
C	-1.928289475	-0.590688317	-0.908770225
C	-2.326469231	-0.456981719	-2.245260778
C	-3.571761864	-0.937329380	-2.657941270
C	-4.444553140	-1.523358254	-1.736413008
C	-4.072197554	-1.632633217	-0.399717026
C	2.041669725	-0.321099892	-2.255089271
C	2.837564949	-0.023732395	-3.360103325
C	2.120174726	2.270043760	-3.241633926
C	1.354572560	1.938795519	-2.144284702
C	1.860071000	-1.637678977	-1.654354015
C	0.848435244	-1.714300921	-0.658250926
C	0.626892706	-2.955199453	-0.040520113
C	1.367254094	-4.082570585	-0.399198713
C	2.352433812	-3.994074415	-1.389525556
C	2.599193947	-2.776294293	-2.014579667
H	-5.421399837	-1.889540048	-2.061274948
H	-4.765808718	-2.080764606	0.315980399
H	-1.662130740	0.011255550	-2.978411241
H	-3.865851608	-0.845062169	-3.707272026
H	-0.142038788	-3.052716869	0.730318029
H	3.376168405	-2.712087133	-2.780949654
H	1.173502445	-5.039509072	0.093749128
H	2.929831787	-4.878127744	-1.671627657
H	0.488166530	-0.382341387	2.873513475
C	-2.492175143	-1.729994781	3.752430510
H	-4.080427796	-2.087944794	2.337306645
H	-3.053346024	-2.111470361	4.608598297
C	-1.193517258	-1.249218286	3.911114111
H	-0.695161178	-1.240494955	4.881847396
C	2.897995057	1.275271946	-3.858114626
H	0.754262027	2.685985127	-1.622256567
H	2.126262749	3.301932362	-3.597258255
H	3.535018357	1.512115012	-4.712549183
H	3.416481885	-0.821549304	-3.828387411
C	3.472032720	1.223382720	2.276806899
C	-0.256472247	2.721383293	1.072509304
N	-0.931056514	3.861315919	1.326920432
N	-0.934257568	1.986997670	0.190959481
C	1.036296515	2.282385122	1.573697052
C	1.921187538	3.064255295	2.322530790
C	3.148115408	2.519701640	2.685410000
C	2.537830534	0.507268316	1.540571956
N	1.345673098	1.017748375	1.219979510
H	1.656582133	4.082241943	2.613962556
H	3.854171319	3.106436339	3.277091397
H	4.428595401	0.764991743	2.533588954
H	2.731420936	-0.515801344	1.204977737
C	-2.070354013	2.674007116	-0.119263571
C	-2.078628614	3.853482105	0.588136316
H	-0.649365415	4.599117731	1.965585052
H	-2.806770794	2.289473279	-0.821393592
H	-2.797153750	4.668373261	0.626357854

$(^3LC_{ppy\ 1}/^3MC_{ax\ 2})_{ci\text{-}neb}$ UPBE0/DEF2-SVP CPCM (CH_3CN).

Ir	0.198428105	-0.176878243	0.032380334
N	-0.998784534	0.424857452	-1.832372892
C	-1.251428514	-0.614127211	-2.660882117
C	-1.995242633	-0.420863264	-3.831066662
C	-2.470284506	0.846672096	-4.140839681
C	-2.190303240	1.906436818	-3.279264351
C	-1.449009501	1.645147438	-2.133480125
N	1.047676119	-0.334579805	1.902640268
C	0.226775509	-0.933913593	2.842313572
C	0.777642376	-1.552678279	3.963033865

C	2.158003400	-1.587324532	4.142954516
C	2.979977777	-0.984621154	3.174505506
C	2.399096975	-0.366947788	2.088452176
H	-2.199665081	-1.263971080	-4.491834217
H	-3.055490304	1.005630743	-5.049914624
H	-2.539537039	2.919959198	-3.483494606
H	3.000897742	0.139916572	1.331578152
H	4.066645757	-0.974960554	3.278858218
H	2.591514889	-2.082574743	5.014169218
H	0.113076522	-2.021695513	4.690811376
C	-0.000988453	-1.897321871	-1.007082385
C	-1.447364645	-0.605077343	1.125120923
C	-1.185749747	-0.900578003	2.488136766
C	-2.235718506	-1.145460158	3.388391683
C	-3.553572877	-1.099068796	2.946685188
C	-3.826448973	-0.814352123	1.604106154
C	-2.784593007	-0.571606278	0.706096164
H	-2.021604196	-1.353526374	4.440280450
H	-4.371907912	-1.276123262	3.649234359
H	-4.861939108	-0.780598646	1.253925092
C	-0.698640299	-1.902570969	-2.229174963
C	-0.822892921	-3.093807528	-2.961829713
C	-0.262879138	-4.272998844	-2.481748134
C	0.425714787	-4.270346112	-1.265291352
C	0.557407995	-3.090643268	-0.530462725
H	-1.357479698	-3.105845415	-3.914643380
H	-0.362944047	-5.197531429	-3.055164471
H	0.865142713	-5.195635830	-0.882213831
H	1.098016967	-3.110800156	0.421481893
N	1.944348716	0.570878126	-1.040478298
H	-3.027302139	-0.352187964	-0.337009289
N	0.253063408	2.072170200	0.456445312
C	1.367477370	2.690271696	0.005915823
C	1.707905930	3.976909732	0.430790920
C	0.876421517	4.621673686	1.340596323
C	-0.268206247	3.970228049	1.803812754
C	-0.540438088	2.691194084	1.335685855
H	-1.419474794	2.132471536	1.668998243
H	1.127764134	5.623218545	1.696853314
H	-0.942781057	4.442148491	2.520253278
H	2.618160013	4.458188418	0.068540495
C	2.169977470	1.876691229	-0.896143432
C	2.871073198	0.095873977	-1.920479407
N	3.218523820	2.242881536	-1.660375998
C	3.675951830	1.138802528	-2.317659783
H	-1.211501438	2.443545088	-1.428238486
H	3.593359944	3.182276042	-1.758441068
H	2.908741886	-0.950059100	-2.216464889
H	4.515385775	1.183231948	-3.006997310

$(^3LC_{ppy\ 2}/^3MC_{ax\ 2})_{ci\text{-}neb}$ UPBE0/DEF2-SVP CPCM (CH_3CN).

Ir	0.135502466	-0.236988699	0.053445321
N	-1.091032105	0.415551301	-1.717146869
C	-1.311315031	-0.549912935	-2.638695548
C	-2.045130353	-0.272102925	-3.797991619
C	-2.562980710	1.001739575	-3.992853862
C	-2.337758264	1.979243716	-3.025427464
C	-1.591760845	1.639410198	-1.904686622
N	0.982936776	-0.330800137	1.921232626
C	0.165996018	-0.859086383	2.902041371
C	0.710752921	-1.278574537	4.114392587
C	2.080016345	-1.172024944	4.348131814
C	2.897747076	-0.644066452	3.334588983
C	2.320869084	-0.236874865	2.151075017
H	-2.203212608	-1.051649016	-4.544187700
H	-3.137644503	1.231347490	-4.893150620
H	-2.727985955	2.993075259	-3.127317575
H	2.919687378	0.198972469	1.349562313
H	3.975095617	-0.531131562	3.470224755
H	2.507348581	-1.496289693	5.298890872
H	0.052154862	-1.694393009	4.879012292

C	0.054323516	-1.877117447	-1.130570512
C	-1.499494473	-0.692105550	1.138066745
C	-1.237039545	-0.945372235	2.512264671
C	-2.281474939	-1.265282286	3.394553299
C	-3.589882487	-1.339978833	2.927603624
C	-3.862155252	-1.102642559	1.575343922
C	-2.827775613	-0.782671449	0.694992903
H	-2.075149626	-1.444960244	4.452923186
H	-4.402319196	-1.579989866	3.618309608
H	-4.889009048	-1.169379912	1.204950958
C	-0.717789860	-1.846879719	-2.309159638
C	-0.858891490	-3.001371329	-3.091469844
C	-0.226650941	-4.181399752	-2.709416836
C	0.561309570	-4.209737808	-1.555306174
C	0.714329356	-3.060701326	-0.775549224
H	-1.465714044	-2.986329373	-4.000304191
H	-0.341009426	-5.081200592	-3.318379327
H	1.067916128	-5.133327263	-1.261683829
H	1.339653111	-3.100458559	0.121924179
N	1.936287363	0.425402191	-1.001312968
H	-3.064768781	-0.611928521	-0.358259211
N	0.324718418	2.042248010	0.416982324
C	1.458396722	2.604055178	-0.052931530
C	1.839829132	3.897168048	0.316560428
C	1.021386889	4.612446745	1.182428081
C	-0.141995223	4.014454516	1.674824538
C	-0.447473127	2.721693457	1.271407659
H	-1.345852610	2.208045591	1.626089925
H	1.292916481	5.627627160	1.479841107
H	-0.811195580	4.542808887	2.355906022
H	2.766653388	4.330614631	-0.063649557
C	2.228830819	1.722225792	-0.913781683
C	2.865174219	-0.143070193	-1.822257477
N	3.318800295	1.994098364	-1.659762828
C	3.737507939	0.833445922	-2.243068343
H	-1.388862528	2.376674403	-1.128659553
H	3.745875278	2.906526841	-1.792469297
H	2.858994620	-1.204941659	-2.057640253
H	4.602313795	0.795865888	-2.900480977

($^{3}MC_{ax\,2}$)$_{min}$ UPBE0/DEF2-SVP CPCM (CH_3CN).

Ir	0.404045000	-0.182426000	-0.078440000
N	-0.185835000	-0.955290000	2.247712000
N	1.900029000	0.465409000	-1.597450000
C	-1.941001000	-2.046163000	3.438200000
C	-1.402852000	-1.529034000	2.249271000
C	0.519780000	-0.883123000	3.374309000
C	-2.089709000	-1.576990000	0.947131000
C	-1.422077000	-1.034606000	-0.172218000
C	-2.068834000	-1.063750000	-1.418813000
C	-3.335577000	-1.631412000	-1.561782000
C	-3.990561000	-2.167376000	-0.450621000
C	-3.370900000	-2.134230000	0.793646000
C	2.587547000	-0.564646000	-2.142644000
C	3.425651000	-0.335170000	-3.242020000
C	2.816437000	1.992211000	-3.185473000
C	2.005266000	1.701137000	-2.098005000
C	2.360514000	-1.869011000	-1.505874000
C	1.353452000	-1.920763000	-0.504115000
C	1.128276000	-3.164140000	0.110595000
C	1.860610000	-4.299101000	-0.238477000
C	2.849029000	-4.226971000	-1.224410000
C	3.095554000	-3.013367000	-1.856574000
H	-4.985397000	-2.607384000	-0.553371000
H	-3.897615000	-2.549478000	1.655744000
H	-1.577065000	-0.642595000	-2.302262000
H	-3.813602000	-1.651270000	-2.545165000
H	0.359146000	-3.251051000	0.882439000
H	3.874071000	-2.959361000	-2.621771000
H	1.658592000	-5.250632000	0.262113000
H	3.426405000	-5.113849000	-1.496766000

H	1.504405000	-0.413325000	3.312588000
C	-1.206797000	-1.967302000	4.614709000
H	-2.927767000	-2.509776000	3.442671000
H	-1.616975000	-2.367813000	5.545123000
C	0.055077000	-1.375798000	4.589480000
H	0.671242000	-1.294695000	5.486777000
C	3.539223000	0.947166000	-3.764218000
H	1.420286000	2.480447000	-1.601601000
H	2.879225000	3.013525000	-3.564487000
H	4.188351000	1.131672000	-4.623702000
H	3.978132000	-1.161307000	-3.690642000
C	4.073246000	1.262770000	2.256714000
C	0.231716000	2.548149000	1.175854000
N	-0.469433000	3.672852000	1.425975000
N	-0.479300000	1.740195000	0.392921000
C	1.568041000	2.179582000	1.616246000
C	2.415043000	3.001200000	2.364472000
C	3.682178000	2.529274000	2.689365000
C	3.170291000	0.504853000	1.517841000
N	1.950541000	0.951027000	1.215839000
H	2.092020000	3.993986000	2.682272000
H	4.363567000	3.151412000	3.274035000
H	5.061468000	0.861171000	2.486558000
H	3.425185000	-0.495548000	1.157202000
C	-1.663919000	2.364061000	0.138512000
C	-1.667776000	3.580243000	0.782524000
H	-0.169743000	4.458361000	1.996328000
H	-2.436356000	1.912689000	-0.480172000
H	-2.414074000	4.369034000	0.833326000

($^3MC_{ax\,2}/S_0)_{stc\text{-}mecp}$ UPBE0/DEF2-SVP CPCM (CH_3CN).

Ir	0.381677000	-0.176702000	-0.153571000
N	-0.146602000	-0.982084000	2.271377000
N	1.934651000	0.476531000	-1.614540000
C	-1.882702000	-2.083947000	3.481646000
C	-1.365139000	-1.549774000	2.290435000
C	0.579254000	-0.930657000	3.385710000
C	-2.084537000	-1.572203000	1.004840000
C	-1.453243000	-1.007096000	-0.123915000
C	-2.140995000	-1.009451000	-1.349120000
C	-3.409470000	-1.578376000	-1.467662000
C	-4.027226000	-2.139768000	-0.348329000
C	-3.368902000	-2.129101000	0.876387000
C	2.636741000	-0.552278000	-2.142446000
C	3.530199000	-0.315221000	-3.195681000
C	2.941743000	2.017363000	-3.132772000
C	2.075741000	1.718018000	-2.090618000
C	2.366430000	-1.864815000	-1.538432000
C	1.323687000	-1.920489000	-0.573983000
C	1.058305000	-3.171278000	0.008222000
C	1.785631000	-4.310828000	-0.336900000
C	2.809324000	-4.235047000	-1.285472000
C	3.096808000	-3.013383000	-1.884289000
H	-5.023715000	-2.580720000	-0.428404000
H	-3.868569000	-2.563722000	1.744830000
H	-1.679550000	-0.563999000	-2.237735000
H	-3.917399000	-1.577018000	-2.436037000
H	0.260867000	-3.259420000	0.750488000
H	3.903113000	-2.957611000	-2.619848000
H	1.552121000	-5.268418000	0.137693000
H	3.382996000	-5.125300000	-1.554633000
H	1.564255000	-0.462371000	3.315244000
C	-1.124870000	-2.028380000	4.644348000
H	-2.872056000	-2.541573000	3.499853000
H	-1.518267000	-2.442379000	5.576122000
C	0.138268000	-1.441070000	4.602840000
H	0.773025000	-1.376261000	5.488479000
C	3.681847000	0.973779000	-3.691235000
H	1.474225000	2.495836000	-1.611994000
H	3.031991000	3.043342000	-3.493103000
H	4.374456000	1.164422000	-4.514718000

H	4.096180000	-1.139642000	-3.630217000
C	3.997349000	1.240905000	2.289119000
C	0.194967000	2.555352000	1.112321000
N	-0.503224000	3.686375000	1.341160000
N	-0.496425000	1.755676000	0.303825000
C	1.513830000	2.174914000	1.593006000
C	2.343825000	2.985044000	2.372279000
C	3.599973000	2.504173000	2.725452000
C	3.109688000	0.493634000	1.521025000
N	1.899994000	0.948258000	1.194055000
H	2.016092000	3.975800000	2.691475000
H	4.268418000	3.117202000	3.334193000
H	4.978087000	0.833100000	2.539247000
H	3.369262000	-0.504659000	1.157757000
C	-1.665854000	2.391600000	0.011247000
C	-1.679970000	3.606480000	0.657313000
H	-0.214341000	4.468927000	1.921231000
H	-2.421791000	1.949393000	-0.633826000
H	-2.419702000	4.402606000	0.683674000

(^{3}MLCT/^{3}MC$_{eq\ 1}$)$_{ts}$ UPBE0/DEF2-SVP CPCM (CH_3CN).

Ir	-0.235563000	-0.244696000	0.218964000
N	-1.115685000	0.315046000	-1.553484000
C	-0.992291000	-0.567633000	-2.582750000
C	-1.534860000	-0.263802000	-3.836015000
C	-2.195709000	0.941521000	-4.028930000
C	-2.310130000	1.833562000	-2.960599000
C	-1.758707000	1.479378000	-1.739693000
N	0.655786000	-0.843213000	1.968861000
C	-0.173459000	-1.108143000	3.011760000
C	0.357582000	-1.514462000	4.239520000
C	1.734460000	-1.624656000	4.390600000
C	2.567105000	-1.321981000	3.312314000
C	1.986079000	-0.934695000	2.113641000
H	-1.429401000	-0.973814000	-4.657135000
H	-2.618778000	1.186488000	-5.005860000
H	-2.822413000	2.791292000	-3.063674000
H	2.575400000	-0.687576000	1.226159000
H	3.653427000	-1.386366000	3.390819000
H	2.157180000	-1.939088000	5.347705000
H	-0.309931000	-1.731963000	5.074011000
C	0.287170000	-1.821508000	-0.924820000
C	-1.878117000	-0.347502000	1.432548000
C	-1.593058000	-0.902305000	2.711832000
C	-2.627195000	-1.214030000	3.603520000
C	-3.951524000	-0.980616000	3.239601000
C	-4.248515000	-0.441439000	1.983055000
C	-3.223453000	-0.134370000	1.088478000
H	-2.408266000	-1.645249000	4.583714000
H	-4.757113000	-1.225199000	3.936170000
H	-5.289642000	-0.260186000	1.701374000
C	-0.268968000	-1.787232000	-2.235064000
C	-0.126599000	-2.881320000	-3.099461000
C	0.569889000	-4.012281000	-2.683174000
C	1.119772000	-4.060106000	-1.395272000
C	0.979218000	-2.980263000	-0.526704000
H	-0.565489000	-2.859034000	-4.100171000
H	0.681018000	-4.862948000	-3.359869000
H	1.665142000	-4.950542000	-1.069671000
H	1.417393000	-3.045714000	0.472607000
N	2.425045000	0.419717000	-1.145778000
H	-3.482214000	0.281125000	0.110181000
N	0.590782000	1.843677000	0.570486000
C	1.461464000	2.491227000	-0.230586000
C	1.725659000	3.855549000	-0.057677000
C	1.101329000	4.549375000	0.970690000
C	0.237764000	3.860309000	1.818918000
C	0.013438000	2.510886000	1.579759000
H	-0.673855000	1.937701000	2.206813000
H	1.302907000	5.612698000	1.119225000
H	-0.264344000	4.352701000	2.653216000

H	2.443998000	4.357110000	-0.708437000
C	2.156247000	1.708435000	-1.250072000
C	3.112629000	0.086897000	-2.272227000
N	2.661250000	2.217500000	-2.404311000
C	3.269348000	1.198684000	-3.073210000
H	-1.830669000	2.137566000	-0.871143000
H	2.562322000	3.168956000	-2.744996000
H	3.468735000	-0.926208000	-2.455908000
H	3.746411000	1.342742000	-4.039847000

$(^3LC_{ppy\ 1}/^3MC_{eq\ 1})_{ts}$ UPBE0/DEF2-SVP CPCM (CH_3CN).

Ir	-0.228033000	-0.244787000	0.218228000
N	-1.097787000	0.316854000	-1.558083000
C	-0.974083000	-0.567978000	-2.585596000
C	-1.509267000	-0.263102000	-3.841815000
C	-2.163007000	0.945286000	-4.039469000
C	-2.277899000	1.839427000	-2.972822000
C	-1.734090000	1.484206000	-1.748844000
N	0.654729000	-0.846880000	1.971324000
C	-0.179690000	-1.113724000	3.009662000
C	0.345199000	-1.523605000	4.238932000
C	1.721220000	-1.634955000	4.396398000
C	2.559328000	-1.330008000	3.322938000
C	1.984241000	-0.939631000	2.122480000
H	-1.403515000	-0.974879000	-4.661382000
H	-2.580191000	1.191096000	-5.018715000
H	-2.784819000	2.799593000	-3.079692000
H	2.578977000	-0.691787000	1.238992000
H	3.645215000	-1.395184000	3.406512000
H	2.139142000	-1.952033000	5.354731000
H	-0.326432000	-1.742822000	5.069660000
C	0.292699000	-1.825145000	-0.920424000
C	-1.875429000	-0.345768000	1.424579000
C	-1.597502000	-0.905233000	2.703493000
C	-2.636515000	-1.218436000	3.588995000
C	-3.958636000	-0.981561000	3.219379000
C	-4.248627000	-0.437407000	1.963276000
C	-3.218791000	-0.129144000	1.074708000
H	-2.423130000	-1.653514000	4.568693000
H	-4.768045000	-1.227267000	3.911105000
H	-5.288070000	-0.253276000	1.677266000
C	-0.258601000	-1.790545000	-2.232894000
C	-0.118949000	-2.887160000	-3.094532000
C	0.570850000	-4.020569000	-2.673835000
C	1.116648000	-4.068429000	-1.384100000
C	0.978261000	-2.986411000	-0.518006000
H	-0.554630000	-2.864672000	-4.096650000
H	0.680106000	-4.873083000	-3.348511000
H	1.657102000	-4.960653000	-1.055219000
H	1.412947000	-3.052106000	0.482820000
N	2.401458000	0.434733000	-1.174456000
H	-3.471985000	0.290148000	0.096561000
N	0.597752000	1.841700000	0.584849000
C	1.462220000	2.496731000	-0.216856000
C	1.739692000	3.855645000	-0.024341000
C	1.131828000	4.537100000	1.022144000
C	0.271679000	3.840845000	1.867895000
C	0.035686000	2.496743000	1.610414000
H	-0.648671000	1.918627000	2.236136000
H	1.343335000	5.596220000	1.186121000
H	-0.218544000	4.323599000	2.714806000
H	2.455046000	4.362361000	-0.674482000
C	2.134212000	1.725062000	-1.259895000
C	3.060290000	0.110546000	-2.320495000
N	2.612008000	2.243832000	-2.421275000
C	3.200603000	1.229528000	-3.114430000
H	-1.806565000	2.143716000	-0.881351000
H	2.506283000	3.198362000	-2.751322000
H	3.408600000	-0.901779000	-2.522489000
H	3.654147000	1.381572000	-4.091113000

($^3LC_{ppy\ 2}/^3MC_{eq\ 1})_{ts}$ UPBE0/DEF2-SVP CPCM (CH_3CN).

Ir	-0.236449000	-0.243622000	0.217720000
N	-1.104717000	0.321623000	-1.558726000
C	-0.980711000	-0.561160000	-2.587874000
C	-1.515336000	-0.253882000	-3.843717000
C	-2.168949000	0.954932000	-4.039330000
C	-2.284218000	1.846974000	-2.971071000
C	-1.740877000	1.489320000	-1.747564000
N	0.643502000	-0.847987000	1.971363000
C	-0.191953000	-1.109718000	3.010079000
C	0.331334000	-1.519889000	4.239896000
C	1.706937000	-1.637178000	4.397242000
C	2.546113000	-1.337714000	3.323128000
C	1.972614000	-0.946378000	2.122149000
H	-1.409372000	-0.964090000	-4.664609000
H	-2.585756000	1.202615000	-5.018265000
H	-2.791060000	2.807369000	-3.076219000
H	2.567196000	-0.701542000	1.237523000
H	3.631719000	-1.407695000	3.406604000
H	2.123621000	-1.954635000	5.355996000
H	-0.341082000	-1.734863000	5.071102000
C	0.283657000	-1.822484000	-0.924097000
C	-1.885295000	-0.338983000	1.423520000
C	-1.609105000	-0.896446000	2.703603000
C	-2.649058000	-1.203852000	3.590010000
C	-3.970440000	-0.963326000	3.220042000
C	-4.258704000	-0.421232000	1.962715000
C	-3.227845000	-0.118518000	1.073336000
H	-2.436953000	-1.637271000	4.570717000
H	-4.780567000	-1.204592000	3.912495000
H	-5.297534000	-0.234299000	1.676304000
C	-0.265623000	-1.784670000	-2.237160000
C	-0.124695000	-2.879105000	-3.101347000
C	0.563549000	-4.013981000	-2.682042000
C	1.106592000	-4.065346000	-1.391352000
C	0.967446000	-2.985164000	-0.523009000
H	-0.558302000	-2.853901000	-4.104294000
H	0.673617000	-4.864914000	-3.358577000
H	1.645530000	-4.958839000	-1.063427000
H	1.400253000	-3.053359000	0.478452000
N	2.431211000	0.410810000	-1.134420000
H	-3.479773000	0.299382000	0.094270000
N	0.596591000	1.840808000	0.575560000
C	1.473282000	2.485506000	-0.221238000
C	1.743377000	3.848250000	-0.045192000
C	1.118517000	4.543375000	0.982012000
C	0.248452000	3.857114000	1.825836000
C	0.018564000	2.509146000	1.583683000
H	-0.673878000	1.938263000	2.207179000
H	1.324677000	5.605461000	1.133097000
H	-0.254472000	4.350596000	2.658997000
H	2.466643000	4.347404000	-0.692365000
C	2.167543000	1.700510000	-1.239433000
C	3.118329000	0.074712000	-2.260187000
N	2.675692000	2.207177000	-2.393327000
C	3.280313000	1.185632000	-3.061322000
H	-1.813934000	2.147331000	-0.879000000
H	2.580519000	3.158782000	-2.734672000
H	3.470400000	-0.939912000	-2.443243000
H	3.758871000	1.327454000	-4.027552000

($^3MLCT/^3MC_{eq\ 1})_{ci\text{-}neb}$ UPBE0/DEF2-SVP CPCM (CH_3CN).

Ir	-0.195846314	-0.259431588	0.173448021
N	-1.120780919	0.291376459	-1.578198416
C	-1.056850125	-0.604194569	-2.600495925
C	-1.641544331	-0.302302570	-3.835641953
C	-2.275343943	0.918744821	-4.018966441
C	-2.321458801	1.827410752	-2.959645099
C	-1.737901678	1.470178391	-1.755193385
N	0.716962338	-0.828944237	1.925781374

C	-0.103821430	-1.087170245	2.977994307
C	0.436296820	-1.461179351	4.212304187
C	1.814768776	-1.547794324	4.362175582
C	2.638428615	-1.257941412	3.273484471
C	2.047885617	-0.904514291	2.069384589
H	-1.587560162	-1.024525815	-4.651175442
H	-2.730324163	1.163717778	-4.981346141
H	-2.804820729	2.800540833	-3.055832967
H	2.632049495	-0.670543510	1.176389840
H	3.725765520	-1.305312974	3.347604635
H	2.245302780	-1.834121941	5.324603544
H	-0.225861154	-1.669195648	5.053668662
C	0.280442222	-1.855484384	-0.986885953
C	-1.822371098	-0.335336061	1.409723900
C	-1.526312897	-0.910355181	2.680666164
C	-2.549528997	-1.284022489	3.561747970
C	-3.881274017	-1.095495441	3.200115502
C	-4.193587158	-0.530452798	1.958160449
C	-3.178958648	-0.157610447	1.076501148
H	-2.316483230	-1.737320358	4.529007311
H	-4.678837573	-1.397446322	3.883843162
H	-5.240861036	-0.385156069	1.677411551
C	-0.345420570	-1.834040034	-2.266521307
C	-0.284684471	-2.947843260	-3.115949590
C	0.398084134	-4.092360308	-2.715462617
C	1.025165846	-4.127334879	-1.462923520
C	0.972617651	-3.023259750	-0.612999508
H	-0.779231882	-2.931254061	-4.090694277
H	0.438337913	-4.962168368	-3.376170019
H	1.560300443	-5.029065409	-1.151061421
H	1.478962779	-3.080072393	0.354583055
N	2.224857746	0.457495874	-1.090582338
H	-3.456561249	0.279427764	0.112933865
N	0.530481426	1.960300771	0.539549874
C	1.471145561	2.595054278	-0.188144042
C	1.755081227	3.952340206	0.005693132
C	1.058959533	4.658896479	0.978842863
C	0.103260145	3.990604757	1.740569145
C	-0.124721064	2.642689432	1.484719415
H	-0.875416890	2.082081353	2.047723299
H	1.270387536	5.717441818	1.148437114
H	-0.464521555	4.498449502	2.521935733
H	2.531765349	4.442704470	-0.584469868
C	2.194524915	1.775785673	-1.152781771
C	3.001135632	0.042639500	-2.128241653
N	2.934712379	2.225294671	-2.197527142
C	3.454720653	1.137313222	-2.833996876
H	-1.761202764	2.137334102	-0.891661073
H	3.051825275	3.189252282	-2.495693424
H	3.204213308	-1.010877298	-2.313952133
H	4.083647208	1.227052596	-3.716789559

($^{3}LC_{ppy\ 1}$/$^{3}MC_{eq\ 1}$)$_{ci\text{-}neb}$ UPBE0/DEF2-SVP CPCM (CH_3CN).

Ir	-0.163637456	-0.202044149	0.148794863
N	-1.072444785	0.352759196	-1.608831703
C	-1.062857834	-0.584707695	-2.599855543
C	-1.679791342	-0.314151283	-3.827599790
C	-2.285050501	0.915117830	-4.042235314
C	-2.271130865	1.868114378	-3.018536010
C	-1.661094200	1.543531173	-1.818809421
N	0.750027736	-0.794701908	1.891514197
C	-0.076064164	-1.096717759	2.927559509
C	0.461878765	-1.517195516	4.147976379
C	1.840396357	-1.598050368	4.301791505
C	2.668693324	-1.251455705	3.233033824
C	2.081706818	-0.856011003	2.040444110
H	-1.675969267	-1.072384058	-4.611813133
H	-2.765615042	1.133575476	-4.998609449
H	-2.733539744	2.848893348	-3.140373467
H	2.666841521	-0.579889500	1.159657282
H	3.756188417	-1.291481393	3.312712320

H	2.268130859	-1.925063533	5.252370428
H	-0.202495621	-1.770892189	4.974553197
C	0.245671650	-1.818992295	-0.951328376
C	-1.792013432	-0.357187813	1.349313160
C	-1.498228425	-0.921436089	2.624754804
C	-2.529930256	-1.272925489	3.503918353
C	-3.857683061	-1.080437292	3.128524014
C	-4.163119236	-0.552661620	1.867697561
C	-3.142238283	-0.201127737	0.987217125
H	-2.306164805	-1.701890324	4.484042746
H	-4.660759442	-1.349559235	3.818653734
H	-5.207485279	-0.412464102	1.574776816
C	-0.379963862	-1.817905765	-2.234857701
C	-0.335087211	-2.961185485	-3.046097952
C	0.333327280	-4.099909918	-2.609127422
C	0.946997042	-4.115710029	-1.346226242
C	0.902453382	-2.992698470	-0.528110877
H	-0.825429821	-2.967119217	-4.022914834
H	0.376026611	-4.984477083	-3.249086077
H	1.468534665	-5.014433420	-1.005503290
H	1.384702254	-3.028467719	0.452131201
N	2.294025109	0.461418519	-1.054749757
H	-3.402531421	0.204968861	0.005251224
N	0.530988852	1.926625910	0.579153473
C	1.469594076	2.572775309	-0.140986787
C	1.749302953	3.925617748	0.077910071
C	1.053758933	4.612372129	1.065282750
C	0.106950770	3.927818540	1.823933288
C	-0.118912946	2.585318121	1.546837543
H	-0.862739894	2.011077391	2.104950437
H	1.259955663	5.669031496	1.249432430
H	-0.457656376	4.418329142	2.618540964
H	2.524323323	4.423809329	-0.506838579
C	2.193104550	1.776234637	-1.124832897
C	3.042430995	0.078126231	-2.125107215
N	2.861539157	2.254061652	-2.204053196
C	3.406212597	1.188782728	-2.857223305
H	-1.643842996	2.248186490	-0.984881953
H	2.908449772	3.220572318	-2.511879737
H	3.292419954	-0.965156273	-2.314022768
H	3.988844179	1.303373483	-3.768236513

($(^3LC_{ppy\ 2}/^3MC_{eq\ 1})_{ci\text{-}neb}$ UPBE0/DEF2-SVP CPCM (CH_3CN).

Ir	-0.198523374	-0.213072893	0.158990644
N	-1.113367763	0.346929970	-1.595395258
C	-1.076388062	-0.567495416	-2.604882548
C	-1.688701806	-0.283430317	-3.831245528
C	-2.320907899	0.936655352	-4.022582172
C	-2.332841110	1.867390613	-2.980158833
C	-1.722833830	1.529423332	-1.783753185
N	0.715968901	-0.801773431	1.902424543
C	-0.106210199	-1.086431706	2.946055478
C	0.435792106	-1.492115767	4.169418466
C	1.814554873	-1.578234944	4.317275951
C	2.639051980	-1.252281461	3.239521177
C	2.047602097	-0.869508884	2.044738842
H	-1.661619296	-1.022135645	-4.633303183
H	-2.802610223	1.164035378	-4.976173608
H	-2.813568759	2.841300473	-3.083990545
H	2.626743943	-0.604538753	1.156315550
H	3.726542437	-1.296286483	3.313422062
H	2.245932233	-1.892985124	5.270551235
H	-0.225300861	-1.729757097	5.003584781
C	0.245067212	-1.811621860	-0.974206128
C	-1.829182805	-0.352657475	1.371477069
C	-1.530326099	-0.909865527	2.648278202
C	-2.556367118	-1.257902526	3.534999493
C	-3.886783165	-1.073370007	3.165196857
C	-4.198418674	-0.546419163	1.906231582
C	-3.181761364	-0.193336562	1.019868159
H	-2.326396197	-1.678276293	4.517677237

H	-4.686008052	-1.347032896	3.858401913
H	-5.244339998	-0.407214257	1.618892948
C	-0.370817485	-1.795874126	-2.260403875
C	-0.300129161	-2.917760103	-3.098258275
C	0.375508065	-4.059376826	-2.678500202
C	0.978406506	-4.091635984	-1.412391212
C	0.916660610	-2.983432554	-0.572772803
H	-0.775644442	-2.905912304	-4.082263479
H	0.431434598	-4.930819721	-3.335164086
H	1.507137536	-4.991425339	-1.085271435
H	1.398244784	-3.033007400	0.407351839
N	2.359878992	0.413763756	-0.988703436
H	-3.449850926	0.215832362	0.041532431
N	0.533304281	1.927273629	0.546260140
C	1.500593619	2.549180515	-0.158457028
C	1.784239055	3.905347525	0.040858193
C	1.065287112	4.620317173	0.991060646
C	0.087801868	3.961376405	1.733049224
C	-0.140696869	2.615029570	1.476987143
H	-0.907609032	2.060953156	2.024600483
H	1.277074566	5.678478679	1.160940596
H	-0.496678527	4.474603208	2.498367542
H	2.581589847	4.387673023	-0.527474409
C	2.252367569	1.725358759	-1.100935042
C	3.138608287	0.003647412	-2.026620352
N	2.946535052	2.174380618	-2.177482601
C	3.514277722	1.093009506	-2.783276078
H	-1.720825145	2.216558691	-0.935435715
H	2.999024132	3.130282445	-2.514937764
H	3.399948797	-1.042947936	-2.177566617
H	4.119529464	1.181135232	-3.682725028

$(^3MC_{eq\ 1})_{min}$ UPBE0/DEF2-SVP CPCM (CH_3CN).

Ir	-0.295560000	-0.230041000	0.264784000
N	-1.191571000	0.369073000	-1.490389000
C	-1.023449000	-0.456766000	-2.558697000
C	-1.567118000	-0.115653000	-3.801363000
C	-2.273170000	1.071345000	-3.943890000
C	-2.430802000	1.906583000	-2.836650000
C	-1.875958000	1.515877000	-1.628078000
N	0.600655000	-0.854732000	2.003791000
C	-0.226048000	-1.130404000	3.045765000
C	0.307765000	-1.544703000	4.269316000
C	1.685328000	-1.653208000	4.416979000
C	2.514878000	-1.339951000	3.339733000
C	1.931975000	-0.943247000	2.144563000
H	-1.427840000	-0.781192000	-4.653920000
H	-2.697693000	1.345383000	-4.912439000
H	-2.978426000	2.848059000	-2.899705000
H	2.515881000	-0.678084000	1.256727000
H	3.601553000	-1.401328000	3.416514000
H	2.110357000	-1.973889000	5.371019000
H	-0.357779000	-1.769778000	5.103349000
C	0.285121000	-1.740847000	-0.947269000
C	-1.938879000	-0.374144000	1.472700000
C	-1.646990000	-0.928173000	2.750138000
C	-2.677310000	-1.244605000	3.644437000
C	-4.003903000	-1.019431000	3.283680000
C	-4.306727000	-0.482799000	2.027840000
C	-3.285438000	-0.168328000	1.131120000
H	-2.453211000	-1.673301000	4.624553000
H	-4.806336000	-1.268793000	3.982168000
H	-5.349477000	-0.309465000	1.747339000
C	-0.250805000	-1.661070000	-2.262519000
C	-0.039995000	-2.696840000	-3.182838000
C	0.696792000	-3.818285000	-2.812130000
C	1.219116000	-3.914631000	-1.516268000
C	1.013997000	-2.890166000	-0.594374000
H	-0.455641000	-2.638141000	-4.191883000
H	0.860093000	-4.624357000	-3.531471000
H	1.793823000	-4.798711000	-1.225782000

H	1.433824000	-2.990558000	0.409878000
N	2.710913000	0.328966000	-0.918077000
H	-3.549645000	0.244326000	0.153350000
N	0.611217000	1.808632000	0.550231000
C	1.504417000	2.389990000	-0.276155000
C	1.751288000	3.766800000	-0.215094000
C	1.095051000	4.540410000	0.733956000
C	0.219211000	3.917785000	1.620217000
C	0.007294000	2.551818000	1.489469000
H	-0.691276000	2.025791000	2.145314000
H	1.283823000	5.614690000	0.794963000
H	-0.303494000	4.474136000	2.399877000
H	2.483853000	4.214085000	-0.889346000
C	2.256452000	1.537269000	-1.199559000
C	3.412885000	-0.069689000	-2.014477000
N	2.655362000	1.930771000	-2.437567000
C	3.386956000	0.916618000	-2.977758000
H	-1.979499000	2.130302000	-0.730884000
H	2.414531000	2.800051000	-2.904326000
H	3.907388000	-1.039217000	-2.063977000
H	3.812241000	0.975764000	-3.976886000

($(^3MC_{eq\ 1}/S_0)_{stc\text{-}mecp}$ UPBE0/DEF2-SVP CPCM (CH_3CN).

Ir	-0.348795000	0.536710000	0.208951000
N	0.693904000	-1.879032000	-1.950228000
N	-0.850525000	-1.612556000	0.594741000
N	1.358070000	0.351430000	1.350755000
N	-2.037967000	0.717434000	-0.944927000
N	2.114544000	-3.126589000	-0.807753000
C	0.896424000	-2.523375000	-0.815130000
C	-1.849304000	-1.773851000	1.476293000
C	-2.106311000	-2.981809000	2.110302000
C	-1.288061000	-4.070583000	1.819197000
C	-0.279506000	-3.917210000	0.876138000
C	-0.091189000	-2.675203000	0.258000000
C	3.752354000	0.054056000	2.713217000
C	-3.378567000	0.143521000	-2.831156000
C	-4.379357000	1.005851000	-2.382852000
C	3.393210000	1.806204000	-1.355850000
C	3.141358000	2.301118000	-2.632449000
C	1.845948000	2.246339000	-3.159937000
C	0.804164000	1.689147000	-2.419634000
C	1.034718000	1.169012000	-1.135442000
C	2.351299000	1.245436000	-0.604711000
C	1.374438000	-0.160086000	2.591536000
C	2.549209000	-0.328153000	3.308044000
C	3.735420000	0.582708000	1.429281000
C	2.520645000	0.723169000	0.750789000
C	-3.501274000	3.130849000	1.427471000
C	-3.121901000	3.678227000	2.651217000
C	-1.929289000	3.274490000	3.259980000
C	-1.114961000	2.320196000	2.649164000
C	-1.479550000	1.741348000	1.423216000
C	-2.689624000	2.170033000	0.811239000
C	-2.217195000	0.026344000	-2.080733000
C	-3.009869000	1.541397000	-0.473843000
C	-4.194820000	1.705462000	-1.196352000
H	-2.446874000	-0.885492000	1.696590000
H	-2.929220000	-3.053927000	2.823226000
H	-1.447300000	-5.036617000	2.303797000
H	0.345620000	-4.763233000	0.584811000
H	4.664490000	0.881021000	0.942616000
H	4.698386000	-0.061784000	3.247079000
H	2.513065000	-0.748434000	4.314155000
H	0.406618000	-0.436005000	3.016240000
H	-3.488369000	-0.433373000	-3.750685000
H	-5.304495000	1.125838000	-2.951511000
H	-4.972949000	2.370631000	-0.820599000
H	-1.390615000	-0.632823000	-2.366431000
H	3.954441000	2.739085000	-3.216328000
H	1.649443000	2.641663000	-4.160608000

H	4.404341000	1.869307000	-0.945765000
H	-0.197276000	1.658530000	-2.856746000
H	-1.633241000	3.709622000	4.218655000
H	-0.184298000	2.026322000	3.142360000
H	-4.429207000	3.463413000	0.955644000
H	-3.755555000	4.428796000	3.129881000
C	2.725222000	-2.838544000	-1.990741000
H	2.521661000	-3.660059000	-0.045503000
C	1.822637000	-2.064791000	-2.689147000
H	3.725208000	-3.193293000	-2.229231000
H	1.937889000	-1.641018000	-3.686213000

(^{3}MLCT/^{3}MC$_{eq\ 2}$)$_{ts}$ UPBE0/DEF2-SVP CPCM (CH_3CN).

Ir	-0.041545000	-0.339908000	-0.194733000
N	-1.100053000	0.066976000	-1.916403000
C	-1.234446000	-0.964044000	-2.791089000
C	-1.942170000	-0.783610000	-3.983508000
C	-2.485557000	0.460188000	-4.278991000
C	-2.310564000	1.512524000	-3.379002000
C	-1.610991000	1.272070000	-2.205478000
N	0.945555000	-0.749550000	1.551554000
C	0.153371000	-0.901041000	2.655403000
C	0.729690000	-1.198731000	3.897543000
C	2.105108000	-1.326407000	4.010348000
C	2.899286000	-1.159232000	2.868322000
C	2.280533000	-0.877662000	1.663509000
H	-2.052923000	-1.616456000	-4.678933000
H	-3.037553000	0.610687000	-5.209696000
H	-2.712297000	2.507795000	-3.574835000
H	2.855703000	-0.753613000	0.743706000
H	3.985781000	-1.251164000	2.907155000
H	2.560458000	-1.553507000	4.976927000
H	0.088282000	-1.319867000	4.771555000
C	0.211823000	-2.095426000	-1.179999000
C	-1.591044000	-0.310813000	1.055925000
C	-1.262176000	-0.720190000	2.388710000
C	-2.276344000	-0.946816000	3.332930000
C	-3.608071000	-0.750200000	2.988767000
C	-3.946464000	-0.338647000	1.686741000
C	-2.956708000	-0.126497000	0.736944000
H	-2.026708000	-1.283449000	4.342670000
H	-4.392533000	-0.920665000	3.730133000
H	-4.996199000	-0.181815000	1.422930000
C	-0.565994000	-2.196195000	-2.368034000
C	-0.656171000	-3.408807000	-3.062750000
C	0.030874000	-4.527727000	-2.597013000
C	0.803674000	-4.443828000	-1.432198000
C	0.885811000	-3.244177000	-0.727667000
H	-1.263842000	-3.490026000	-3.967567000
H	-0.038350000	-5.472549000	-3.141577000
H	1.344215000	-5.324531000	-1.073958000
H	1.489331000	-3.203959000	0.183970000
N	1.641028000	0.816630000	-1.049733000
H	-3.246409000	0.189735000	-0.268550000
N	0.180861000	2.337320000	0.731858000
C	1.366351000	2.823702000	0.350853000
C	1.918565000	3.981121000	0.909706000
C	1.193650000	4.643686000	1.896655000
C	-0.039849000	4.131879000	2.294221000
C	-0.502068000	2.966668000	1.680778000
H	-1.463708000	2.523777000	1.961800000
H	1.595373000	5.550068000	2.355795000
H	-0.636820000	4.621891000	3.065866000
H	2.895280000	4.353980000	0.594450000
C	2.032318000	2.033411000	-0.682845000
C	2.489665000	0.397642000	-2.037750000
N	3.110266000	2.394978000	-1.408008000
C	3.417485000	1.382916000	-2.269634000
H	-1.451293000	2.051453000	-1.456865000
H	3.600639000	3.282553000	-1.345167000
H	2.387196000	-0.576387000	-2.510613000

H	4.250678000	1.443846000	-2.965131000

$(^{3}LC_{ppy\ 1}/^{3}MC_{eq\ 2})_{ts}$ UPBE0/DEF2-SVP CPCM (CH_3CN).

Ir	-0.280519000	-0.079576000	0.256626000
N	-1.197951000	0.432667000	-1.498944000
C	-1.273059000	-0.558629000	-2.437140000
C	-1.910091000	-0.317692000	-3.662178000
C	-2.449722000	0.930723000	-3.928288000
C	-2.352911000	1.935475000	-2.956175000
C	-1.725952000	1.643007000	-1.758395000
N	0.640867000	-0.660033000	2.007755000
C	-0.180329000	-0.896169000	3.064457000
C	0.355723000	-1.290377000	4.294401000
C	1.731598000	-1.418271000	4.435861000
C	2.557637000	-1.145749000	3.344233000
C	1.969857000	-0.770494000	2.145051000
H	-1.970012000	-1.116689000	-4.402547000
H	-2.943396000	1.125882000	-4.882962000
H	-2.763374000	2.933018000	-3.119944000
H	2.563314000	-0.556595000	1.253128000
H	3.643550000	-1.226461000	3.412005000
H	2.158788000	-1.723493000	5.393918000
H	-0.308216000	-1.484406000	5.137561000
C	0.020101000	-1.767388000	-0.757573000
C	-1.886917000	-0.138374000	1.490374000
C	-1.598657000	-0.676161000	2.777065000
C	-2.630560000	-0.961734000	3.679888000
C	-3.953708000	-0.710896000	3.323476000
C	-4.253804000	-0.182990000	2.061431000
C	-3.233261000	0.092575000	1.153613000
H	-2.411106000	-1.385529000	4.663206000
H	-4.757307000	-0.932981000	4.029840000
H	-5.294359000	0.013722000	1.788104000
C	-0.652336000	-1.803510000	-2.022409000
C	-0.708018000	-2.995036000	-2.763309000
C	-0.086166000	-4.142908000	-2.288068000
C	0.586663000	-4.120562000	-1.052404000
C	0.634686000	-2.955766000	-0.298876000
H	-1.243592000	-3.028721000	-3.715677000
H	-0.124937000	-5.064824000	-2.873407000
H	1.078048000	-5.026247000	-0.685733000
H	1.160171000	-2.966861000	0.659651000
H	-3.491418000	0.498462000	0.171062000
C	2.246730000	1.959397000	-1.051287000
H	-1.640410000	2.386371000	-0.963011000
N	0.475387000	1.963044000	0.657202000
C	0.006302000	2.826189000	1.609292000
H	-0.822909000	2.567325000	2.263795000
C	0.741009000	3.984858000	1.554772000
H	0.689381000	4.903074000	2.134253000
N	1.654603000	3.801014000	0.558357000
H	2.365437000	4.472585000	0.283809000
C	1.479036000	2.571929000	0.031794000
N	2.019875000	0.650942000	-1.202992000
C	2.645132000	-0.008765000	-2.170541000
H	2.417928000	-1.076996000	-2.254796000
C	3.548551000	0.606476000	-3.037963000
H	4.046276000	0.026051000	-3.817248000
C	3.792487000	1.969138000	-2.879671000
H	4.489081000	2.490827000	-3.540164000
C	3.131261000	2.667276000	-1.872347000
H	3.295519000	3.738853000	-1.741931000

$(^{3}LC_{ppy\ 2}/^{3}MC_{eq\ 2})_{ts}$ UPBE0/DEF2-SVP CPCM (CH_3CN).

Ir	-0.041672000	-0.338337000	-0.195347000
N	-1.097676000	0.068682000	-1.918486000
C	-1.233899000	-0.963361000	-2.791699000
C	-1.940676000	-0.783195000	-3.984726000
C	-2.481067000	0.461376000	-4.282406000
C	-2.304083000	1.514732000	-3.383972000

C	-1.605715000	1.274492000	-2.209695000
N	0.942492000	-0.747835000	1.552537000
C	0.148489000	-0.897984000	2.655310000
C	0.722697000	-1.195496000	3.898498000
C	2.097818000	-1.324274000	4.013473000
C	2.893876000	-1.158428000	2.872524000
C	2.277209000	-0.877045000	1.666621000
H	-2.052982000	-1.616901000	-4.678877000
H	-3.032279000	0.611690000	-5.213606000
H	-2.703433000	2.510613000	-3.581564000
H	2.853885000	-0.754034000	0.747625000
H	3.980234000	-1.251255000	2.913062000
H	2.551514000	-1.551202000	4.980869000
H	0.079868000	-1.315582000	4.771611000
C	0.209255000	-2.095210000	-1.178214000
C	-1.592900000	-0.306978000	1.052818000
C	-1.266436000	-0.716031000	2.386360000
C	-2.282326000	-0.941454000	3.329039000
C	-3.613314000	-0.743776000	2.982701000
C	-3.949336000	-0.332441000	1.679938000
C	-2.957952000	-0.121615000	0.731613000
H	-2.034581000	-1.277981000	4.339282000
H	-4.399093000	-0.913242000	3.722897000
H	-4.998516000	-0.174704000	1.414466000
C	-0.568284000	-2.196236000	-2.366458000
C	-0.660698000	-3.409750000	-3.059302000
C	0.023929000	-4.529312000	-2.591583000
C	0.796500000	-4.445170000	-1.426601000
C	0.880750000	-3.244660000	-0.723840000
H	-1.268215000	-3.491109000	-3.964208000
H	-0.046976000	-5.474833000	-3.134714000
H	1.335200000	-5.326397000	-1.066878000
H	1.483996000	-3.204244000	0.187968000
N	1.644237000	0.813692000	-1.050189000
H	-3.245756000	0.194485000	-0.274469000
N	0.186403000	2.334717000	0.731569000
C	1.372715000	2.819932000	0.351587000
C	1.925996000	3.976254000	0.911591000
C	1.201321000	4.638900000	1.898672000
C	-0.033026000	4.128283000	2.295155000
C	-0.496354000	2.964124000	1.680553000
H	-1.458680000	2.522112000	1.960598000
H	1.603868000	5.544444000	2.358742000
H	-0.629821000	4.618423000	3.066853000
H	2.903299000	4.348179000	0.597057000
C	2.038019000	2.029321000	-0.682242000
C	2.492547000	0.393451000	-2.037916000
N	3.117230000	2.389015000	-1.406427000
C	3.422729000	1.376821000	-2.268533000
H	-1.444766000	2.054651000	-1.462164000
H	3.609603000	3.275410000	-1.342577000
H	2.388292000	-0.580051000	-2.511480000
H	4.256540000	1.436325000	-2.963414000

$(^3MLCT/^3MC_{eq\ 2})_{ci\text{-}neb}$ UPBE0/DEF2-SVP CPCM (CH_3CN).

Ir	-0.066739598	-0.347072133	-0.184136412
N	-1.119287226	0.064955719	-1.912131582
C	-1.206519499	-0.947919409	-2.813642024
C	-1.879669862	-0.753192499	-4.023826445
C	-2.441007354	0.485744215	-4.306540060
C	-2.316967040	1.519301052	-3.376928421
C	-1.646581197	1.265670971	-2.189134786
N	0.931110718	-0.739943301	1.556207776
C	0.146896997	-0.860995498	2.672692899
C	0.736757644	-1.135246779	3.915188267
C	2.111725129	-1.270984866	4.017042431
C	2.895968133	-1.138599279	2.861091541
C	2.267036518	-0.879368286	1.657679860
H	-1.950260470	-1.570261868	-4.742892802
H	-2.967544881	0.647430481	-5.250072220
H	-2.733348662	2.510708102	-3.561960102

H	2.834110070	-0.784260958	0.729228140
H	3.981861659	-1.241988468	2.889398214
H	2.575572281	-1.479623452	4.983862435
H	0.103662817	-1.234196094	4.798261197
C	0.188646805	-2.093880603	-1.169061398
C	-1.609104427	-0.323898323	1.066735237
C	-1.268064533	-0.681909943	2.413685918
C	-2.279123742	-0.858186598	3.373812903
C	-3.611002955	-0.664733710	3.030946776
C	-3.960259108	-0.318816338	1.710727761
C	-2.977349234	-0.158285319	0.745924527
H	-2.026158769	-1.149937816	4.396768877
H	-4.390231571	-0.787242861	3.787289189
H	-5.011361638	-0.170462618	1.447645729
C	-0.533132732	-2.178332314	-2.392997202
C	-0.568178562	-3.375639119	-3.118247987
C	0.108508342	-4.496260773	-2.640609901
C	0.813264769	-4.431177332	-1.432206967
C	0.847733003	-3.245001618	-0.701945625
H	-1.124806489	-3.444073592	-4.056290061
H	0.082620747	-5.428895855	-3.209453430
H	1.339093428	-5.315142215	-1.060609049
H	1.400783804	-3.215759765	0.241320999
N	1.606371640	0.776563520	-1.068459594
H	-3.270833842	0.105636842	-0.273554923
N	0.189569738	2.260371190	0.763684408
C	1.356085897	2.768488897	0.353034159
C	1.901586034	3.934667109	0.899220450
C	1.192033932	4.579597222	1.908625211
C	-0.019513213	4.042928602	2.339209038
C	-0.479183962	2.872277666	1.733923056
H	-1.424365092	2.409532604	2.037846117
H	1.588748582	5.492591534	2.359363014
H	-0.601134239	4.519663463	3.130426376
H	2.860986919	4.329015400	0.557331626
C	2.005614206	1.989448774	-0.698601967
C	2.435896048	0.363299622	-2.074344593
N	3.070616816	2.354930069	-1.438797748
C	3.361205160	1.349342086	-2.313798803
H	-1.523352871	2.031899137	-1.420431266
H	3.564056738	3.240611091	-1.373250986
H	2.323812191	-0.607365739	-2.552346681
H	4.183146003	1.413979973	-3.021901099

$(^3LC_{ppy\ 1}/^3MC_{eq\ 2})_{ci\text{-}neb}$ UPBE0/DEF2-SVP CPCM (CH_3CN).

Ir	-0.292915958	-0.073802176	0.243038058
N	-1.236959256	0.461473117	-1.491130305
C	-1.322376021	-0.510641709	-2.448661497
C	-1.998005435	-0.252246991	-3.649605478
C	-2.567280092	0.991732341	-3.870650209
C	-2.455032195	1.978043886	-2.881251314
C	-1.788037118	1.669733597	-1.709946319
N	0.655518687	-0.655598259	1.979743108
C	-0.149148877	-0.886551348	3.050016681
C	0.406742935	-1.278049993	4.271893136
C	1.784927152	-1.407382877	4.391344063
C	2.593300697	-1.138203240	3.286183529
C	1.986214040	-0.766170207	2.095780520
H	-2.068852752	-1.034590986	-4.406697111
H	-3.096735871	1.198274403	-4.803527912
H	-2.883683249	2.973002708	-3.010096405
H	2.562485793	-0.551253821	1.193245074
H	3.680269202	-1.218515340	3.335664837
H	2.227607390	-1.711990845	5.342517647
H	-0.243298368	-1.471950468	5.125874913
C	-0.001339498	-1.741042404	-0.808294390
C	-1.887426900	-0.162704760	1.490766850
C	-1.573374939	-0.674194388	2.782497616
C	-2.588108714	-0.947566537	3.708164690
C	-3.920028354	-0.724749691	3.364936082
C	-4.246434650	-0.240447071	2.092159130

C	-3.242082590	0.032996924	1.165519230
H	-2.348890337	-1.339497181	4.700129246
H	-4.709588472	-0.935681032	4.090357188
H	-5.294283353	-0.074170065	1.826293418
C	-0.677684155	-1.755216302	-2.071959100
C	-0.712805445	-2.926581099	-2.845511368
C	-0.075207868	-4.077879250	-2.400052615
C	0.587762280	-4.081372027	-1.158731799
C	0.620814725	-2.935215518	-0.375560267
H	-1.243332121	-2.941450077	-3.801540436
H	-0.094368634	-4.982757014	-3.012499632
H	1.084567800	-4.991714684	-0.811089193
H	1.137328403	-2.964986954	0.587056688
H	-3.519131199	0.407705556	0.175820856
C	2.278503849	1.916099549	-1.005055894
H	-1.687830184	2.398240840	-0.903046098
N	0.426913167	1.989976665	0.610473207
C	-0.102444135	2.895629510	1.488790064
H	-0.961340486	2.660734833	2.113327137
C	0.618913497	4.061223565	1.408443543
H	0.525862334	5.007219689	1.935481100
N	1.584721920	3.839228866	0.470548074
H	2.304191515	4.504918071	0.203409594
C	1.452119118	2.581255646	0.002294060
N	2.108146023	0.590700371	-1.045128527
C	2.803742656	-0.129505099	-1.917210609
H	2.619376664	-1.209311881	-1.908575471
C	3.725819041	0.439559574	-2.795355834
H	4.290043424	-0.190971244	-3.485225278
C	3.898201573	1.822278303	-2.766087108
H	4.597991530	2.310651650	-3.448183762
C	3.160471110	2.583269267	-1.862367502
H	3.265470702	3.670013603	-1.838727907

$(^3LC_{ppy\ 2}/^3MC_{eq\ 2})_{ci\text{-}neb}$ UPBE0/DEF2-SVP CPCM (CH_3CN).

Ir	-0.068799385	-0.304116618	-0.231652867
N	-1.125508547	0.056964163	-1.970946846
C	-1.231657784	-0.993574420	-2.826780968
C	-1.930086694	-0.850984510	-4.029725582
C	-2.492890231	0.376164860	-4.356419780
C	-2.344072105	1.450398178	-3.477734734
C	-1.652533110	1.246940563	-2.292135814
N	0.918764673	-0.670014314	1.516499395
C	0.122596580	-0.807147148	2.624183182
C	0.701569196	-1.081621427	3.872030789
C	2.076171066	-1.200590405	3.989500108
C	2.872801229	-1.051054812	2.842729009
C	2.255653819	-0.793111037	1.633504300
H	-2.021448267	-1.700591501	-4.707432025
H	-3.040373599	0.496337075	-5.294175028
H	-2.760965197	2.434058740	-3.699040494
H	2.832070444	-0.686715846	0.711862118
H	3.959433419	-1.140942938	2.884618428
H	2.531247463	-1.409336559	4.960260107
H	0.058975137	-1.194878374	4.746363439
C	0.176380962	-2.075931437	-1.152102358
C	-1.624934094	-0.309739102	0.995639476
C	-1.289909237	-0.647958579	2.349958773
C	-2.307743304	-0.824464163	3.303210049
C	-3.637908623	-0.651324194	2.946192746
C	-3.981253258	-0.329592109	1.617322257
C	-2.992640502	-0.170552067	0.659079581
H	-2.058709016	-1.097096262	4.332185554
H	-4.421533819	-0.770057197	3.698437376
H	-5.031766761	-0.196993227	1.344217449
C	-0.551305331	-2.205844408	-2.368322396
C	-0.580852880	-3.428162328	-3.050564306
C	0.106662468	-4.525994962	-2.537047656
C	0.813333190	-4.415861024	-1.332653010
C	0.840841684	-3.206237235	-0.643101298
H	-1.138719803	-3.532649112	-3.984338281

H	0.089398452	-5.476370096	-3.075693922
H	1.347565797	-5.282400765	-0.933292618
H	1.391998961	-3.140544462	0.299297472
N	1.580598553	0.836766012	-1.121602998
H	-3.280085438	0.074054286	-0.366900105
N	0.207809432	2.285636135	0.797417097
C	1.405333629	2.747609327	0.421618851
C	2.030493633	3.824928417	1.056249854
C	1.371630146	4.432175314	2.121505688
C	0.128370431	3.943971994	2.517591704
C	-0.412295931	2.861062608	1.822098028
H	-1.386574820	2.444178797	2.099327516
H	1.833297531	5.275042154	2.640993818
H	-0.417295442	4.391433799	3.350659447
H	3.014316488	4.175571070	0.738281222
C	2.008705323	2.020422717	-0.692646726
C	2.371223639	0.476228709	-2.178928924
N	3.054898222	2.416740182	-1.443839294
C	3.301901903	1.463615771	-2.388148050
H	-1.514662335	2.046978525	-1.561237083
H	3.560481303	3.291954970	-1.344594418
H	2.231182618	-0.460787366	-2.713406963
H	4.100818127	1.564005636	-3.118370292

($^{3}MC_{eq\ 2}$)$_{min}$ UPBE0/DEF2-SVP CPCM (CH_3CN).

Ir	-0.145757000	-0.429658000	-0.343554000
N	-1.189243000	-0.158395000	-2.096682000
C	-1.212892000	-1.211084000	-2.956044000
C	-1.883869000	-1.101813000	-4.177872000
C	-2.509045000	0.091586000	-4.516655000
C	-2.452908000	1.166877000	-3.629410000
C	-1.781793000	0.998560000	-2.427307000
N	0.853267000	-0.711680000	1.424196000
C	0.089160000	-0.685414000	2.550589000
C	0.687043000	-0.847792000	3.805208000
C	2.060674000	-1.025064000	3.898522000
C	2.826558000	-1.040311000	2.730517000
C	2.182216000	-0.883107000	1.513820000
H	-1.904112000	-1.950003000	-4.863209000
H	-3.032659000	0.184827000	-5.470917000
H	-2.921934000	2.125258000	-3.857120000
H	2.731608000	-0.897339000	0.569796000
H	3.908791000	-1.176299000	2.754983000
H	2.533819000	-1.149230000	4.875309000
H	0.069922000	-0.827159000	4.704181000
C	0.230843000	-2.216983000	-1.261213000
C	-1.689118000	-0.231696000	0.939269000
C	-1.332932000	-0.474220000	2.297785000
C	-2.314976000	-0.513979000	3.297225000
C	-3.652071000	-0.309120000	2.969332000
C	-4.016850000	-0.072621000	1.637136000
C	-3.049827000	-0.037074000	0.635488000
H	-2.041368000	-0.709877000	4.337173000
H	-4.415098000	-0.338678000	3.750891000
H	-5.068352000	0.086588000	1.381599000
C	-0.475315000	-2.385501000	-2.483668000
C	-0.439478000	-3.607571000	-3.167273000
C	0.295085000	-4.671659000	-2.648862000
C	0.992110000	-4.521909000	-1.444858000
C	0.954504000	-3.309722000	-0.756087000
H	-0.986610000	-3.739411000	-4.104135000
H	0.321107000	-5.624349000	-3.183390000
H	1.568038000	-5.359980000	-1.042216000
H	1.502981000	-3.218160000	0.185805000
N	1.362907000	0.976596000	-1.101773000
H	-3.363690000	0.146331000	-0.395417000
N	0.116849000	2.493027000	0.990797000
C	1.383205000	2.750673000	0.655452000
C	2.206491000	3.599748000	1.406208000
C	1.674169000	4.194123000	2.546843000
C	0.356097000	3.917256000	2.901574000

C	-0.379619000	3.054234000	2.087549000
H	-1.419243000	2.809969000	2.332708000
H	2.289475000	4.859213000	3.157458000
H	-0.098510000	4.358210000	3.791017000
H	3.245044000	3.779520000	1.120646000
C	1.867169000	2.083355000	-0.557532000
C	2.089335000	0.714423000	-2.234690000
N	2.896246000	2.518630000	-1.314136000
C	3.054177000	1.677985000	-2.376414000
H	-1.711093000	1.803009000	-1.691503000
H	3.436116000	3.362999000	-1.148561000
H	1.884195000	-0.147732000	-2.865401000
H	3.819163000	1.831593000	-3.133177000

($^{3}MC_{eq\ 2}/S_0$)$_{stc\text{-}mecp}$ UPBE0/DEF2-SVP CPCM (CH_3CN).

Ir	0.898950000	0.633984000	-1.119302000
N	1.154632000	-1.539689000	-1.266147000
N	-0.295726000	-1.508596000	1.298378000
N	2.604302000	0.745398000	0.021539000
N	-0.818502000	0.543269000	-2.252833000
N	0.895179000	-3.707771000	-1.262056000
C	0.496179000	-2.548223000	-0.697682000
C	-1.170442000	-1.390520000	2.292561000
C	-2.282885000	-2.220963000	2.435506000
C	-2.490391000	-3.215597000	1.483237000
C	-1.588187000	-3.338503000	0.430126000
C	-0.499383000	-2.458687000	0.381065000
C	4.970719000	0.927692000	1.438617000
C	-2.074467000	-0.147575000	-4.164919000
C	-3.221309000	0.440450000	-3.629264000
C	4.308144000	2.739079000	-2.570505000
C	3.982553000	3.205356000	-3.842225000
C	2.743243000	2.888318000	-4.408047000
C	1.828991000	2.103156000	-3.705487000
C	2.135495000	1.608277000	-2.427162000
C	3.395977000	1.947210000	-1.861517000
C	2.717177000	0.153514000	1.219820000
C	3.886966000	0.223147000	1.963256000
C	4.854387000	1.522269000	0.187872000
C	3.655088000	1.413606000	-0.521561000
C	-2.678558000	2.442896000	0.300941000
C	-2.375621000	3.005445000	1.537574000
C	-1.092422000	2.855090000	2.077344000
C	-0.116186000	2.136434000	1.389291000
C	-0.399496000	1.545817000	0.145555000
C	-1.702274000	1.719927000	-0.398207000
C	-0.893079000	-0.072034000	-3.443257000
C	-1.927667000	1.111124000	-1.707776000
C	-3.145400000	1.070930000	-2.394543000
H	-0.975395000	-0.595561000	3.020791000
H	-2.967055000	-2.085098000	3.275611000
H	-3.352280000	-3.883838000	1.550697000
H	-1.738639000	-4.090503000	-0.347473000
H	5.696031000	2.062118000	-0.247441000
H	5.905992000	1.006002000	1.997885000
H	3.939318000	-0.269187000	2.935516000
H	1.828611000	-0.387691000	1.561635000
H	-2.086515000	-0.656280000	-5.129968000
H	-4.169717000	0.404472000	-4.170233000
H	-4.031216000	1.528787000	-1.953133000
H	0.033477000	-0.509885000	-3.821275000
H	4.695388000	3.824243000	-4.392535000
H	2.489183000	3.259027000	-5.405112000
H	5.275187000	3.002768000	-2.135033000
H	0.864424000	1.876126000	-4.167937000
H	-0.854011000	3.303768000	3.045937000
H	0.876979000	2.036593000	1.834885000
H	-3.678669000	2.578022000	-0.118488000
H	-3.137752000	3.569907000	2.080159000
C	1.840296000	-3.441737000	-2.208890000
H	0.583780000	-4.635688000	-0.988220000

C	1.996207000	-2.080264000	-2.205406000
H	2.320716000	-4.224741000	-2.789798000
H	2.647839000	-1.460542000	-2.817480000

Table S9. Cartesian coordinates (Å) of the optimized geometries of complex **2**.

$(S_0)_{min}$ PBE0/DEF2-SVP CPCM (CH_3CN).

C	3.353899000	3.152454000	-0.885670000
C	2.405157000	2.121451000	-0.955453000
C	2.097599000	1.391715000	0.227926000
C	2.753448000	1.726747000	1.418923000
C	3.682865000	2.758774000	1.427689000
C	4.005165000	3.489912000	0.290012000
C	1.670264000	1.718486000	-2.153338000
N	0.783611000	0.706544000	-1.937944000
C	0.044188000	0.230245000	-2.950176000
C	0.136202000	0.729451000	-4.239984000
C	1.033818000	1.767103000	-4.483697000
C	1.802400000	2.262556000	-3.438838000
Ir	0.674521000	-0.004452000	-0.008293000
C	2.117165000	-1.383248000	-0.232146000
C	2.422139000	-2.109808000	0.953953000
C	3.385125000	-3.128146000	0.893672000
C	4.054235000	-3.455138000	-0.274818000
C	3.735389000	-2.726546000	-1.415039000
C	2.791374000	-1.707674000	-1.415927000
C	1.671800000	-1.715967000	2.145230000
N	0.775392000	-0.714357000	1.922375000
C	0.022442000	-0.246352000	2.928525000
C	0.109855000	-0.744066000	4.219200000
C	1.017377000	-1.771242000	4.470465000
C	1.799912000	-2.258074000	3.431994000
N	-1.030027000	-1.309247000	-0.310390000
C	-2.249766000	-0.743155000	-0.166342000
C	-3.411734000	-1.498127000	-0.302831000
C	-3.354613000	-2.868585000	-0.590488000
C	-2.077383000	-3.419370000	-0.731146000
C	-0.954576000	-2.613900000	-0.583671000
C	-2.256201000	0.705604000	0.137986000
N	-1.045581000	1.281344000	0.282996000
C	-0.981745000	2.590112000	0.558658000
C	-2.110517000	3.379153000	0.706695000
C	-3.386529000	2.814442000	0.565278000
C	-3.430909000	1.448754000	0.275117000
H	-0.524306000	-0.328294000	5.003418000
H	-0.669759000	0.559432000	2.680881000
H	-0.486959000	0.306789000	-5.029331000
H	-0.640807000	-0.583386000	-2.708157000
C	-4.638978000	3.669956000	0.733038000
C	-4.639292000	-3.681849000	-0.725693000
H	0.018974000	3.013867000	0.669015000
H	0.049731000	-3.029732000	-0.693573000
H	2.508988000	3.070798000	-3.610110000
H	2.514490000	-3.057913000	3.609326000
H	2.556671000	1.200576000	2.354999000
H	2.598310000	-1.182896000	-2.353581000
H	4.797044000	-4.253261000	-0.290791000
H	4.736739000	4.298173000	0.313257000
F	3.665065000	3.863253000	-1.974669000
F	4.296307000	3.068600000	2.570919000
F	4.367607000	-3.025943000	-2.550819000
F	3.693819000	-3.836010000	1.985306000
H	-4.379598000	-1.012901000	-0.179969000
H	-1.928823000	-4.475080000	-0.955817000
H	-4.390232000	0.950894000	0.151593000
H	-1.977892000	4.438262000	0.933783000
H	1.137429000	2.190712000	-5.485389000
H	1.118008000	-2.193262000	5.473129000
C	-5.918780000	2.872808000	0.475972000
C	-4.669397000	4.212239000	2.171966000
C	-4.576142000	4.842320000	-0.259511000
H	-5.474680000	5.468967000	-0.148596000
H	-3.698349000	5.481802000	-0.084315000
H	-4.536960000	4.479998000	-1.298640000
H	-6.789604000	3.534374000	0.596075000

H	-5.948622000	2.464294000	-0.546075000
H	-6.034604000	2.039762000	1.186348000
H	-5.566194000	4.835296000	2.314255000
H	-4.703799000	3.390353000	2.903887000
H	-3.788622000	4.833641000	2.393346000
C	-4.352644000	-5.143070000	-1.076025000
C	-5.395060000	-3.629887000	0.612643000
C	-5.508773000	-3.068340000	-1.835516000
H	-6.437392000	-3.650606000	-1.939980000
H	-5.790247000	-2.028191000	-1.612575000
H	-4.984730000	-3.083696000	-2.803725000
H	-6.326498000	-4.212184000	0.534842000
H	-4.789184000	-4.059358000	1.425604000
H	-5.665385000	-2.600417000	0.892169000
H	-5.303577000	-5.687816000	-1.173815000
H	-3.814173000	-5.235820000	-2.031787000
H	-3.760779000	-5.643744000	-0.294474000

$(^3MLCT)_{min}$ UPBE0/DEF2-SVP CPCM (CH_3CN).

C	3.290879000	3.217213000	-0.840071000
C	2.363169000	2.173510000	-0.919554000
C	2.067676000	1.442169000	0.272931000
C	2.678114000	1.800411000	1.485209000
C	3.581216000	2.851190000	1.503124000
C	3.905563000	3.570550000	0.354672000
C	1.647182000	1.752484000	-2.123067000
N	0.800072000	0.707724000	-1.914687000
C	0.052773000	0.224254000	-2.913506000
C	0.114276000	0.749143000	-4.197328000
C	0.980275000	1.811819000	-4.439187000
C	1.750524000	2.318722000	-3.398370000
Ir	0.731894000	-0.008851000	0.014245000
C	2.055467000	-1.472519000	-0.249246000
C	2.351324000	-2.202737000	0.943416000
C	3.272089000	-3.252633000	0.861911000
C	3.877569000	-3.613770000	-0.335141000
C	3.551213000	-2.895950000	-1.483858000
C	2.655519000	-1.838707000	-1.463830000
C	1.643304000	-1.774685000	2.149424000
N	0.801210000	-0.725479000	1.942871000
C	0.060577000	-0.235869000	2.943675000
C	0.124873000	-0.758367000	4.228305000
C	0.986333000	-1.825148000	4.468600000
C	1.748998000	-2.338777000	3.425481000
N	-0.969943000	-1.308210000	-0.264016000
C	-2.207845000	-0.704869000	-0.131956000
C	-3.379746000	-1.499395000	-0.258379000
C	-3.324555000	-2.859229000	-0.488583000
C	-2.027946000	-3.437745000	-0.602207000
C	-0.914390000	-2.634021000	-0.481372000
C	-2.205706000	0.692469000	0.133558000
N	-0.967305000	1.288162000	0.288274000
C	-0.909253000	2.617478000	0.505311000
C	-2.019864000	3.422365000	0.600596000
C	-3.322591000	2.848722000	0.458169000
C	-3.381041000	1.491960000	0.233045000
H	-0.492960000	-0.327393000	5.017285000
H	-0.593444000	0.601850000	2.695124000
H	-0.509474000	0.323191000	-4.984372000
H	-0.604597000	-0.610746000	-2.664949000
C	-4.556559000	3.742121000	0.550813000
C	-4.608733000	-3.687413000	-0.592489000
H	0.089372000	3.044843000	0.619931000
H	0.083695000	-3.067342000	-0.576478000
H	2.429277000	3.150987000	-3.568292000
H	2.423858000	-3.174446000	3.594244000
H	2.465992000	1.274352000	2.417304000
H	2.441248000	-1.314060000	-2.396247000
H	4.586137000	-4.443038000	-0.366983000
H	4.619924000	4.394901000	0.385009000
F	3.614976000	3.923666000	-1.919863000

F	4.162897000	3.192793000	2.646120000
F	4.123046000	-3.245223000	-2.629543000
F	3.597454000	-3.957973000	1.942122000
H	-4.347599000	-1.007711000	-0.153533000
H	-1.882092000	-4.500209000	-0.789439000
H	-4.344614000	1.000104000	0.109869000
H	-1.880438000	4.487521000	0.788683000
H	1.057989000	2.250307000	-5.436591000
H	1.066328000	-2.261719000	5.466675000
C	-5.855423000	2.958115000	0.359067000
C	-4.585675000	4.414194000	1.933733000
C	-4.469195000	4.822722000	-0.540074000
H	-5.343398000	5.490896000	-0.482928000
H	-3.566005000	5.441597000	-0.430450000
H	-4.451367000	4.368257000	-1.543255000
H	-6.714035000	3.643240000	0.429724000
H	-5.896871000	2.471991000	-0.628000000
H	-5.983550000	2.182061000	1.129681000
H	-5.463201000	5.075496000	2.014088000
H	-4.649833000	3.662160000	2.735862000
H	-3.687447000	5.024956000	2.110213000
C	-4.316160000	-5.154533000	-0.917646000
C	-5.352281000	-3.622961000	0.751616000
C	-5.505175000	-3.113515000	-1.701076000
H	-6.430237000	-3.706448000	-1.781683000
H	-5.792479000	-2.071060000	-1.497341000
H	-4.995376000	-3.143132000	-2.676889000
H	-6.278167000	-4.218666000	0.702700000
H	-4.729791000	-4.025930000	1.566072000
H	-5.628754000	-2.590261000	1.012866000
H	-5.263274000	-5.709647000	-0.998378000
H	-3.782388000	-5.262006000	-1.874600000
H	-3.714002000	-5.636726000	-0.132410000

$(^3LC_{ppy\ 1})_{min}$ UPBE0/DEF2-SVP CPCM (CH_3CN).

C	-3.364142000	-3.165929000	-0.801681000
C	-2.423008000	-2.132002000	-0.914851000
C	-2.090335000	-1.384211000	0.249759000
C	-2.707838000	-1.701555000	1.464907000
C	-3.630855000	-2.738031000	1.515356000
C	-3.980143000	-3.486980000	0.397472000
C	-1.713459000	-1.754292000	-2.137068000
N	-0.811669000	-0.749403000	-1.959501000
C	-0.079553000	-0.306422000	-2.991086000
C	-0.199722000	-0.831763000	-4.268417000
C	-1.119621000	-1.857735000	-4.475810000
C	-1.878135000	-2.320808000	-3.408663000
Ir	-0.672189000	0.003844000	-0.038358000
C	-2.076115000	1.386063000	-0.294548000
C	-2.376852000	2.136760000	0.945253000
C	-3.377678000	3.175916000	0.855999000
C	-4.022463000	3.472002000	-0.307897000
C	-3.702686000	2.728022000	-1.469109000
C	-2.757014000	1.705338000	-1.464205000
C	-1.664459000	1.768996000	2.085575000
N	-0.723571000	0.720254000	1.866546000
C	0.028723000	0.303007000	2.876872000
C	-0.051955000	0.829324000	4.167037000
C	-0.991682000	1.868533000	4.430706000
C	-1.777830000	2.323859000	3.411337000
N	1.050092000	1.299409000	-0.372874000
C	2.265033000	0.732389000	-0.201226000
C	3.431153000	1.480096000	-0.341396000
C	3.381929000	2.843838000	-0.660792000
C	2.108534000	3.395216000	-0.831706000
C	0.981620000	2.596282000	-0.680114000
C	2.265248000	-0.709892000	0.133781000
N	1.053445000	-1.285319000	0.265385000
C	0.985081000	-2.589889000	0.558599000
C	2.111397000	-3.374251000	0.744457000
C	3.389023000	-2.807925000	0.626670000

C	3.437581000	-1.448011000	0.310953000
H	0.599771000	0.432040000	4.945894000
H	0.732688000	-0.503070000	2.658854000
H	0.417284000	-0.436675000	-5.076604000
H	0.620642000	0.502078000	-2.775750000
C	4.639069000	-3.653420000	0.850588000
C	4.671234000	3.649108000	-0.798686000
H	-0.016900000	-3.014022000	0.655158000
H	-0.020670000	3.009890000	-0.817052000
H	-2.600315000	-3.120633000	-3.552430000
H	-2.505006000	3.114564000	3.583554000
H	-2.483024000	-1.158836000	2.384829000
H	-2.572748000	1.170349000	-2.398044000
H	-4.769164000	4.267323000	-0.341839000
H	-4.704979000	-4.299765000	0.453942000
F	-3.698597000	-3.897800000	-1.868641000
F	-4.208197000	-3.035433000	2.679620000
F	-4.343357000	3.035835000	-2.596624000
F	-3.684866000	3.882662000	1.949598000
H	4.395600000	0.994196000	-0.196633000
H	1.966669000	4.445792000	-1.083182000
H	4.398610000	-0.950385000	0.201197000
H	1.975291000	-4.429754000	0.985680000
H	-1.248237000	-2.298173000	-5.467284000
H	-1.081834000	2.291910000	5.432938000
C	5.922545000	-2.859284000	0.602670000
C	4.632791000	-4.150621000	2.306134000
C	4.607291000	-4.857024000	-0.105261000
H	5.505762000	-5.474063000	0.050140000
H	3.728774000	-5.496067000	0.067781000
H	4.594248000	-4.528705000	-1.156257000
H	6.791923000	-3.514376000	0.762069000
H	5.975959000	-2.478795000	-0.429201000
H	6.018879000	-2.006744000	1.292457000
H	5.525559000	-4.768443000	2.490228000
H	4.648441000	-3.306506000	3.012937000
H	3.746395000	-4.765385000	2.523594000
C	4.394294000	5.105550000	-1.175598000
C	5.416316000	3.616219000	0.546180000
C	5.545949000	3.012496000	-1.891218000
H	6.478865000	3.587448000	-1.997862000
H	5.819038000	1.974480000	-1.648752000
H	5.029585000	3.014849000	-2.863641000
H	6.350720000	4.193313000	0.465775000
H	4.806071000	4.061694000	1.347157000
H	5.680423000	2.590610000	0.845067000
H	5.348474000	5.644711000	-1.272629000
H	3.865923000	5.185174000	-2.138124000
H	3.796671000	5.620992000	-0.408132000

(^{3}MLCT/^{3}MC$_{ax\ 1}$)$_{ts}$ UPBE0/DEF2-SVP CPCM (CH_3CN).

N	0.082773000	1.012619000	0.788218000
C	-1.152914000	0.543342000	0.487711000
C	-2.296654000	1.303726000	0.766655000
C	-2.198474000	2.552850000	1.373648000
C	-0.893194000	3.006390000	1.671019000
C	0.199805000	2.226561000	1.359558000
C	-1.200765000	-0.767651000	-0.168923000
C	-2.370789000	-1.521260000	-0.285110000
C	-2.379376000	-2.741794000	-0.965452000
C	-1.158240000	-3.156216000	-1.516828000
C	-0.024369000	-2.372282000	-1.355840000
N	-0.031272000	-1.209107000	-0.697754000
Ir	1.712871000	0.043096000	-0.332884000
C	3.235831000	1.314674000	0.057888000
C	3.338833000	2.392602000	-0.863696000
C	4.355250000	3.341039000	-0.676812000
C	5.262941000	3.264073000	0.366804000
C	5.136433000	2.194573000	1.246256000
C	4.147086000	1.227400000	1.113954000
C	2.345985000	2.403968000	-1.930393000

N	1.336375000	1.485272000	-1.743547000
C	0.345209000	1.371955000	-2.661332000
C	0.309014000	2.145202000	-3.803527000
C	1.331295000	3.078588000	-4.017728000
C	2.348395000	3.203446000	-3.076047000
C	3.057726000	-1.413106000	-0.799502000
C	3.494503000	-2.314990000	0.191637000
C	4.445174000	-3.279687000	-0.176154000
C	4.953438000	-3.374679000	-1.462211000
C	4.488579000	-2.469326000	-2.407983000
C	3.547955000	-1.490091000	-2.105119000
C	2.888772000	-2.155437000	1.515783000
C	3.152874000	-2.945559000	2.642579000
C	2.500940000	-2.673494000	3.839199000
C	1.592810000	-1.619912000	3.896803000
C	1.375335000	-0.877884000	2.742923000
N	2.001713000	-1.135238000	1.593815000
H	5.691248000	-4.136785000	-1.715412000
F	4.901974000	-4.156021000	0.721356000
H	3.225185000	-0.807969000	-2.896394000
F	4.963161000	-2.548093000	-3.648638000
H	4.114885000	0.415668000	1.842706000
F	4.470443000	4.376104000	-1.514006000
F	6.006257000	2.096986000	2.252829000
H	6.040586000	4.018769000	0.489638000
H	0.674974000	-0.042093000	2.737119000
H	3.863941000	-3.765426000	2.575309000
H	2.703909000	-3.284902000	4.721637000
H	1.058755000	-1.366807000	4.814026000
H	-0.437799000	0.642894000	-2.445285000
H	-0.515409000	2.024666000	-4.508208000
H	1.336029000	3.702948000	-4.913740000
H	3.152837000	3.921096000	-3.224605000
H	-3.284044000	-1.147480000	0.178758000
C	-3.667230000	-3.555328000	-1.069666000
H	-1.065465000	-4.089821000	-2.070518000
H	0.934822000	-2.688714000	-1.772420000
H	-3.269236000	0.905049000	0.482499000
C	-3.410224000	3.418668000	1.702074000
H	-0.723079000	3.971250000	2.151687000
H	1.217749000	2.561500000	1.575416000
C	-4.725087000	2.737075000	1.319558000
C	-3.421000000	3.702265000	3.213437000
C	-3.297793000	4.743603000	0.929579000
H	-5.567092000	3.394429000	1.583432000
H	-4.785011000	2.538796000	0.238229000
H	-4.864714000	1.785236000	1.855014000
H	-4.162858000	5.383759000	1.163478000
H	-2.386506000	5.299832000	1.196476000
H	-3.286546000	4.567195000	-0.157320000
H	-4.281915000	4.341194000	3.464943000
H	-3.507987000	2.768259000	3.790107000
H	-2.509567000	4.224379000	3.541086000
C	-3.470535000	-4.832646000	-1.888199000
C	-4.121757000	-3.937956000	0.348710000
C	-4.749515000	-2.697073000	-1.744592000
H	-4.422642000	-5.381632000	-1.942519000
H	-3.152351000	-4.612499000	-2.918951000
H	-2.725864000	-5.502342000	-1.431265000
H	-5.055744000	-4.519030000	0.296165000
H	-3.362465000	-4.554291000	0.854759000
H	-4.312410000	-3.049688000	0.969579000
H	-5.682666000	-3.275910000	-1.827155000
H	-4.972163000	-1.785443000	-1.169704000
H	-4.441953000	-2.397338000	-2.758620000

$(^3MLCT/^3MC_{ax\ 1})_{ci\text{-}neb}$ UPBE0/DEF2-SVP CPCM (CH_3CN).

N	0.134189800	1.052772845	0.635314210
C	-1.115511878	0.548028661	0.412858227
C	-2.252907013	1.274518690	0.802945321
C	-2.145886495	2.514537988	1.418522890

C	-0.832002659	3.025600226	1.582367120
C	0.253479905	2.283365312	1.183090521
C	-1.173915509	-0.752691649	-0.239345289
C	-2.354421958	-1.492015428	-0.388848059
C	-2.360570584	-2.724901768	-1.038181980
C	-1.123974572	-3.180613144	-1.529461189
C	0.012380966	-2.405398088	-1.360250764
N	0.003551633	-1.221144245	-0.736937383
Ir	1.720053622	0.057776179	-0.414029064
C	3.224732617	1.362149695	-0.046846725
C	3.304565330	2.441999796	-0.968808809
C	4.264686846	3.442179294	-0.753703837
C	5.146443378	3.411003826	0.313851235
C	5.054824024	2.332548960	1.184801602
C	4.118629555	1.316358278	1.026312862
C	2.348651712	2.410282058	-2.071287931
N	1.420346532	1.408973676	-1.969055519
C	0.490606573	1.239300734	-2.930469388
C	0.429940158	2.053357595	-4.048025408
C	1.361199302	3.087342603	-4.168899587
C	2.320706322	3.265482393	-3.178660429
C	3.055989342	-1.457033095	-0.744809037
C	3.413096498	-2.347337073	0.288043483
C	4.299815228	-3.390022068	-0.025161192
C	4.831794510	-3.565713145	-1.292554989
C	4.455650175	-2.663105369	-2.278693257
C	3.574136900	-1.614475776	-2.033049141
C	2.804867723	-2.099883947	1.597762182
C	3.041185320	-2.839992106	2.764711198
C	2.399746463	-2.482933160	3.944408373
C	1.531130299	-1.394818030	3.944668931
C	1.343954756	-0.704572600	2.752525503
N	1.958399265	-1.045686734	1.618906490
H	5.519383932	-4.386923363	-1.498080077
F	4.667710156	-4.270274511	0.908628846
H	3.313212110	-0.945711975	-2.857578217
F	4.958909334	-2.811966341	-3.501815899
H	4.105093749	0.504739564	1.755486330
F	4.351313271	4.484947917	-1.583130340
F	5.901926753	2.278025792	2.211628613
H	5.877184059	4.207566469	0.459755562
H	0.676871466	0.157231768	2.706064985
H	3.724281767	-3.685570804	2.742396613
H	2.581556814	-3.054581630	4.858046237
H	1.005272748	-1.078386052	4.847280381
H	-0.223347140	0.427291007	-2.780711141
H	-0.339326665	1.882386713	-4.803089895
H	1.342907927	3.755065243	-5.033830123
H	3.054630825	4.064166563	-3.261415856
H	-3.280181984	-1.089954962	0.022533498
C	-3.664449593	-3.505408925	-1.192340923
H	-1.026780728	-4.134904667	-2.045677200
H	0.978759243	-2.746527147	-1.739058590
H	-3.234063357	0.840452484	0.617863208
C	-3.348252032	3.308085422	1.917559496
H	-0.654279395	3.997731806	2.045341993
H	1.273447399	2.649973435	1.319242285
C	-4.666343582	2.576738144	1.659330521
C	-3.193257573	3.522634543	3.433270578
C	-3.385714853	4.670206401	1.205863182
H	-5.501626341	3.182097617	2.042010879
H	-4.840100391	2.409190280	0.585219186
H	-4.699054636	1.602696787	2.171583146
H	-4.245023062	5.256264448	1.568040413
H	-2.475059068	5.257652482	1.396702841
H	-3.491332533	4.544494393	0.117059088
H	-4.047896937	4.100404902	3.818805846
H	-3.163317856	2.558995624	3.965379985
H	-2.275070921	4.077642145	3.677873236
C	-3.448698308	-4.844703089	-1.899400893
C	-4.259832223	-3.771500650	0.199771311

C	-4.646748088	-2.660852234	-2.021303367
H	-4.411331481	-5.370578675	-1.988070988
H	-3.047543792	-4.712350774	-2.916019678
H	-2.762021870	-5.497748711	-1.338956976
H	-5.204043497	-4.329386666	0.100445393
H	-3.571373254	-4.369982756	0.816518992
H	-4.478648399	-2.837620585	0.738976263
H	-5.595566598	-3.206260519	-2.145175769
H	-4.872530878	-1.700015695	-1.534498215
H	-4.239208606	-2.450712601	-3.022535933

($^{3}MC_{ax\ 1}$)$_{min}$ UPBE0/DEF2-SVP CPCM (CH_3CN).

N	0.914720000	-1.196816000	0.459694000
C	2.153181000	-0.703931000	0.280983000
C	3.277988000	-1.448699000	0.644335000
C	3.143655000	-2.723711000	1.199129000
C	1.833905000	-3.201479000	1.366232000
C	0.758467000	-2.415492000	0.988322000
C	2.237005000	0.641663000	-0.328255000
C	3.444411000	1.308014000	-0.515870000
C	3.488493000	2.575117000	-1.111603000
C	2.260276000	3.115394000	-1.503479000
C	1.087268000	2.401828000	-1.286532000
N	1.064080000	1.196608000	-0.712453000
Ir	-0.740228000	0.053972000	-0.311515000
C	-2.308663000	-1.168407000	0.078347000
C	-2.519498000	-2.243123000	-0.835285000
C	-3.526776000	-3.177825000	-0.547353000
C	-4.339775000	-3.090446000	0.571306000
C	-4.122742000	-2.021055000	1.430228000
C	-3.134749000	-1.071869000	1.205284000
C	-1.645880000	-2.302555000	-2.016518000
N	-0.607835000	-1.433547000	-1.986603000
C	0.252221000	-1.348524000	-3.005484000
C	0.134398000	-2.137158000	-4.141241000
C	-0.925505000	-3.041306000	-4.199200000
C	-1.818903000	-3.128586000	-3.137345000
C	-1.909778000	1.692603000	-0.177770000
C	-2.085566000	2.438448000	1.012941000
C	-2.919776000	3.568281000	0.942731000
C	-3.557595000	3.976832000	-0.218676000
C	-3.351381000	3.219634000	-1.363186000
C	-2.543249000	2.090435000	-1.362863000
C	-1.380714000	1.987183000	2.227995000
C	-1.466610000	2.596760000	3.491480000
C	-0.756239000	2.061474000	4.560177000
C	0.029814000	0.929643000	4.363253000
C	0.066286000	0.382423000	3.084668000
N	-0.611288000	0.894245000	2.061716000
H	-4.192593000	4.863363000	-0.221236000
F	-3.137176000	4.319208000	2.027299000
H	-2.425903000	1.536654000	-2.298865000
F	-3.948357000	3.589784000	-2.495048000
H	-3.020717000	-0.265022000	1.931346000
F	-3.731266000	-4.224499000	-1.354867000
F	-4.892634000	-1.913664000	2.514535000
H	-5.109310000	-3.838512000	0.764424000
H	0.667322000	-0.507968000	2.882517000
H	-2.083770000	3.479082000	3.637036000
H	-0.820767000	2.531491000	5.544599000
H	0.602513000	0.475181000	5.173663000
H	1.063086000	-0.622129000	-2.900270000
H	0.856359000	-2.040960000	-4.953776000
H	-1.062937000	-3.678835000	-5.076024000
H	-2.654135000	-3.823364000	-3.180333000
H	4.369199000	0.834034000	-0.188826000
C	4.825784000	3.282069000	-1.312836000
H	2.188179000	4.093362000	-1.978529000
H	0.121517000	2.816572000	-1.584514000
H	4.268311000	-1.028466000	0.483412000
C	4.339157000	-3.581502000	1.601265000

H	1.636086000	-4.187395000	1.790068000
H	-0.269396000	-2.766032000	1.110103000
C	5.669171000	-2.865425000	1.361691000
C	4.221866000	-3.925509000	3.095247000
C	4.312469000	-4.873692000	0.767258000
H	6.495799000	-3.522217000	1.670843000
H	5.819228000	-2.621151000	0.298777000
H	5.744970000	-1.936007000	1.946860000
H	5.167868000	-5.509650000	1.043328000
H	3.392840000	-5.453051000	0.938319000
H	4.384442000	-4.650476000	-0.308600000
H	5.072844000	-4.555700000	3.396771000
H	4.234595000	-3.014509000	3.713516000
H	3.298385000	-4.480184000	3.318490000
C	4.648528000	4.674834000	-1.919572000
C	5.532335000	3.419021000	0.045862000
C	5.688884000	2.431845000	-2.260672000
H	5.634829000	5.147539000	-2.039670000
H	4.176768000	4.632913000	-2.913302000
H	4.041005000	5.327675000	-1.273869000
H	6.500011000	3.925957000	-0.091245000
H	4.929600000	4.015954000	0.747727000
H	5.731565000	2.440931000	0.508987000
H	6.662078000	2.923126000	-2.416373000
H	5.880536000	1.428205000	-1.851956000
H	5.202571000	2.316277000	-3.241716000

$(^3MC_{ax\ 1}/S_0)_{stc\text{-}mecp}$ UPBE0/DEF2-SVP CPCM (CH_3CN).

N	0.867798000	-1.256152000	0.333845000
C	2.096248000	-0.734675000	0.195091000
C	3.230016000	-1.459382000	0.572773000
C	3.109749000	-2.746708000	1.102157000
C	1.807048000	-3.256160000	1.226443000
C	0.723839000	-2.486432000	0.834362000
C	2.163148000	0.618142000	-0.399087000
C	3.341279000	1.357542000	-0.448005000
C	3.374431000	2.627766000	-1.037819000
C	2.173079000	3.083108000	-1.588425000
C	1.027062000	2.301962000	-1.496475000
N	1.007141000	1.104651000	-0.905826000
Ir	-0.817132000	-0.025672000	-0.505804000
C	-2.426927000	-1.165092000	-0.065466000
C	-2.677719000	-2.283558000	-0.912725000
C	-3.764602000	-3.117941000	-0.605262000
C	-4.598225000	-2.902077000	0.481364000
C	-4.324843000	-1.803116000	1.285292000
C	-3.268303000	-0.940038000	1.030350000
C	-1.754899000	-2.491245000	-2.040309000
N	-0.688151000	-1.656064000	-2.049963000
C	0.227881000	-1.717337000	-3.019974000
C	0.145227000	-2.631609000	-4.061129000
C	-0.941899000	-3.503892000	-4.075439000
C	-1.896196000	-3.437829000	-3.066159000
C	-1.827682000	1.681507000	-0.191907000
C	-1.787142000	2.488458000	0.969743000
C	-2.534427000	3.680255000	0.942221000
C	-3.301081000	4.081720000	-0.140589000
C	-3.319454000	3.253973000	-1.253687000
C	-2.595589000	2.070436000	-1.298539000
C	-1.002938000	2.016730000	2.126035000
C	-0.788748000	2.743273000	3.310205000
C	-0.063200000	2.164307000	4.345217000
C	0.439503000	0.875535000	4.191840000
C	0.202055000	0.229096000	2.982325000
N	-0.486423000	0.781909000	1.988853000
H	-3.867271000	5.012957000	-0.105155000
F	-2.542233000	4.498666000	1.998906000
H	-2.652379000	1.464894000	-2.208657000
F	-4.055658000	3.607216000	-2.305580000
H	-3.111144000	-0.095311000	1.703231000
F	-4.035974000	-4.187036000	-1.362059000

F	-5.108367000	-1.575020000	2.340364000
H	-5.428423000	-3.577690000	0.690140000
H	0.584380000	-0.781616000	2.815182000
H	-1.184728000	3.748925000	3.421667000
H	0.104862000	2.723018000	5.269310000
H	1.005451000	0.378039000	4.981498000
H	1.057493000	-1.008014000	-2.951044000
H	0.913895000	-2.654276000	-4.835219000
H	-1.052480000	-4.238187000	-4.877216000
H	-2.750367000	-4.110134000	-3.076748000
H	4.245544000	0.942616000	-0.002966000
C	4.666427000	3.439611000	-1.043299000
H	2.098343000	4.050906000	-2.083319000
H	0.078999000	2.659559000	-1.904759000
H	4.213784000	-1.015299000	0.435564000
C	4.313480000	-3.586527000	1.518852000
H	1.621308000	-4.254174000	1.626970000
H	-0.299059000	-2.861644000	0.922367000
C	5.633124000	-2.838049000	1.325638000
C	4.167161000	-3.960080000	3.003169000
C	4.335660000	-4.863987000	0.662257000
H	6.466047000	-3.482916000	1.642999000
H	5.803861000	-2.570126000	0.271574000
H	5.674955000	-1.918297000	1.929312000
H	5.197956000	-5.486559000	0.947429000
H	3.425067000	-5.465634000	0.801536000
H	4.428091000	-4.620047000	-0.407520000
H	5.027684000	-4.571154000	3.316807000
H	4.136786000	-3.060347000	3.637173000
H	3.254462000	-4.544757000	3.191473000
C	4.491517000	4.784892000	-1.749718000
C	5.086948000	3.695231000	0.414056000
C	5.761909000	2.637905000	-1.764514000
H	5.444710000	5.333960000	-1.726807000
H	4.203058000	4.659599000	-2.804859000
H	3.734099000	5.412098000	-1.254832000
H	6.020501000	4.278914000	0.433210000
H	4.315055000	4.265137000	0.954310000
H	5.266837000	2.756901000	0.960180000
H	6.698710000	3.216442000	-1.771508000
H	5.966234000	1.677685000	-1.267067000
H	5.478922000	2.431502000	-2.808414000

(^{3}MLCT/^{3}MC$_{eq\ 1}$)$_{ci\text{-}neb}$ UPBE0/DEF2-SVP CPCM (CH_3CN).

Ir	1.522571276	-1.304731827	0.022629518
N	-0.736186950	-0.370073112	-1.549642424
N	2.717276303	-0.946840124	-1.609728072
N	0.311244447	-1.694314618	1.643526721
C	-1.319238859	-3.148623722	2.622215973
C	-0.448158729	-2.825007946	1.573219109
C	0.220675239	-0.889950047	2.714903261
C	-0.235673785	-3.583802780	0.346884625
C	0.627017902	-2.985179907	-0.620477579
C	0.915955611	-3.666649446	-1.813827982
C	0.363556962	-4.920449216	-2.027215322
C	-0.488395406	-5.531052272	-1.109398966
C	-0.771579036	-4.846246135	0.062721189
C	4.057881287	-1.091903164	-1.431959965
C	4.927367201	-0.849311587	-2.502252465
C	3.037931824	-0.287930422	-3.880040964
C	2.222269740	-0.545899137	-2.788946881
C	4.423962832	-1.474812308	-0.066916437
C	3.365482370	-1.500138841	0.889380667
C	3.637743890	-1.860977482	2.215416282
C	4.938056662	-2.187674360	2.579108080
C	5.991749750	-2.175932160	1.671131530
C	5.711387113	-1.819725762	0.359402571
H	-0.914844382	-6.517653741	-1.294552459
F	-1.582782197	-5.446486795	0.936587814
H	1.565227180	-3.242852738	-2.582389762
F	0.646059926	-5.574232075	-3.153504945

H	2.858313682	-1.895048774	2.980345566
F	6.729734847	-1.826081480	-0.503218020
F	5.196769588	-2.526364316	3.840936591
H	7.006191413	-2.443998134	1.969608391
H	0.859028396	-0.004134781	2.712952522
C	-1.411259575	-2.312065610	3.726329656
H	-1.920462523	-4.052689746	2.562468562
H	-2.090899470	-2.561546981	4.544592301
C	-0.629009780	-1.158177411	3.776439729
H	-0.668575124	-0.472240307	4.623808251
C	4.413591130	-0.449503096	-3.730913952
H	1.135903137	-0.431615980	-2.819080820
H	2.592007902	0.034408090	-4.822122299
H	5.089553863	-0.257328031	-4.567257931
H	5.999802017	-0.964101908	-2.364842830
C	1.855684218	2.989369565	0.929701402
C	-1.197186241	0.581426282	-0.736944479
C	-2.564329012	0.766272463	-0.495484650
C	-3.499512461	-0.063987466	-1.122951267
C	-2.988040883	-1.045040629	-1.985203519
C	-1.616307221	-1.157044084	-2.164937298
H	-2.884460187	1.544239979	0.197128748
H	-3.649295697	-1.730119095	-2.519215592
C	-0.157056271	1.440028147	-0.112569137
C	-0.387847420	2.785859991	0.172067252
C	0.615883510	3.599374261	0.718137522
C	2.023659617	1.645243726	0.628764755
N	1.045166776	0.878769080	0.135580548
H	-1.368534717	3.203821784	-0.061975936
H	2.706621015	3.535336475	1.335304863
H	2.982423488	1.152689018	0.809324081
H	-1.206182630	-1.917621314	-2.837599263
C	0.344811133	5.064766768	1.047880241
C	1.571487836	5.748637098	1.656079609
H	1.886129316	5.266011337	2.594292846
H	-1.018750529	6.189402220	2.308446914
C	-0.810398918	5.137067004	2.059976617
H	-0.551483228	4.610748377	2.992058321
H	-1.735699770	4.695679964	1.660751869
H	1.326590322	6.796340857	1.886274745
H	2.427334677	5.751410042	0.963609319
C	-0.045515701	5.803727684	-0.242499624
H	-0.244738634	6.862646425	-0.015229236
H	0.768279549	5.760517889	-0.983019452
H	-0.952580120	5.381368944	-0.700633349
C	-5.007195651	0.065023854	-0.923731635
C	-5.362546725	1.173070051	0.068054069
H	-4.940054122	0.977657189	1.065966781
H	-6.652017633	-1.186246124	-0.251759296
C	-5.563615069	-1.268687495	-0.398280279
H	-5.107366759	-1.534490297	0.568182832
H	-5.382999424	-2.095744849	-1.101254480
H	-6.456098908	1.233184651	0.174300739
H	-5.005780521	2.157431080	-0.272011893
C	-5.641272229	0.389228573	-2.286893351
H	-6.731568414	0.499853027	-2.179707072
H	-5.238429388	1.329790891	-2.694201722
H	-5.454454649	-0.408073154	-3.022100374

$(^{3}MC_{eq\ 1})_{min}$ UPBE0/DEF2-SVP CPCM (CH_3CN).

Ir	1.589802000	-1.215130000	-0.017948000
N	-0.723578000	-0.042948000	-2.022979000
N	2.930536000	-0.987914000	-1.554193000
N	0.228429000	-1.464934000	1.503790000
C	-1.702271000	-2.632837000	2.292861000
C	-0.706781000	-2.440339000	1.326858000
C	0.194135000	-0.684815000	2.595065000
C	-0.534481000	-3.187859000	0.084246000
C	0.503269000	-2.735515000	-0.782334000
C	0.770705000	-3.424156000	-1.973111000
C	0.009433000	-4.538800000	-2.294983000

C	-1.026560000	-4.996232000	-1.486124000
C	-1.278906000	-4.307496000	-0.308740000
C	4.238175000	-1.234666000	-1.270151000
C	5.203612000	-1.086087000	-2.272721000
C	3.475436000	-0.399455000	-3.799874000
C	2.558392000	-0.570073000	-2.772952000
C	4.467434000	-1.610782000	0.126399000
C	3.332911000	-1.560379000	0.987920000
C	3.467354000	-1.895729000	2.340230000
C	4.714653000	-2.266002000	2.827077000
C	5.841821000	-2.330352000	2.015427000
C	5.694751000	-2.002381000	0.674751000
H	-1.619836000	-5.870259000	-1.757592000
F	-2.273492000	-4.761768000	0.457835000
H	1.559120000	-3.113780000	-2.661424000
F	0.268993000	-5.200412000	-3.421370000
H	2.623226000	-1.875193000	3.032844000
F	6.785077000	-2.075861000	-0.090736000
F	4.845741000	-2.570885000	4.116708000
H	6.812975000	-2.631588000	2.410366000
H	0.975509000	0.074563000	2.674147000
C	-1.736107000	-1.821911000	3.420469000
H	-2.445596000	-3.414063000	2.152613000
H	-2.513923000	-1.968500000	4.173395000
C	-0.772448000	-0.826817000	3.578379000
H	-0.759206000	-0.169004000	4.448484000
C	4.818056000	-0.670227000	-3.542245000
H	1.488828000	-0.366784000	-2.892827000
H	3.134227000	-0.059036000	-4.778673000
H	5.569581000	-0.549774000	-4.326053000
H	6.249196000	-1.285445000	-2.050180000
C	1.911184000	3.085122000	0.716234000
C	-1.099818000	0.608621000	-0.920657000
C	-2.393398000	0.537289000	-0.392715000
C	-3.362856000	-0.244054000	-1.030521000
C	-2.956755000	-0.899989000	-2.201555000
C	-1.644713000	-0.775259000	-2.646658000
H	-2.621433000	1.082117000	0.523746000
H	-3.652063000	-1.518398000	-2.772872000
C	-0.066132000	1.469587000	-0.284212000
C	-0.346224000	2.796838000	0.030323000
C	0.640355000	3.645510000	0.552428000
C	2.123907000	1.749241000	0.404877000
N	1.158766000	0.949326000	-0.066077000
H	-1.353659000	3.168195000	-0.166628000
H	2.751982000	3.663815000	1.097244000
H	3.105072000	1.292879000	0.559789000
H	-1.322469000	-1.296477000	-3.554716000
C	0.313833000	5.095403000	0.895562000
C	1.520349000	5.827056000	1.486208000
H	1.874612000	5.351688000	2.414049000
H	-1.078644000	6.157067000	2.179000000
C	-0.830111000	5.115476000	1.922735000
H	-0.539061000	4.595262000	2.848654000
H	-1.741871000	4.639027000	1.532442000
H	1.234905000	6.861545000	1.729283000
H	2.361475000	5.872344000	0.777301000
C	-0.127236000	5.819760000	-0.387353000
H	-0.366004000	6.869353000	-0.155090000
H	0.674427000	5.809967000	-1.142148000
H	-1.023560000	5.360900000	-0.831129000
C	-4.791410000	-0.391467000	-0.510606000
C	-4.987019000	0.310322000	0.834528000
H	-4.319177000	-0.099000000	1.608713000
H	-6.133368000	-2.005504000	0.053227000
C	-5.111880000	-1.885388000	-0.340312000
H	-4.413598000	-2.364979000	0.363732000
H	-5.056529000	-2.427438000	-1.295915000
H	-6.023399000	0.165958000	1.175085000
H	-4.809711000	1.394383000	0.762686000
C	-5.751324000	0.227926000	-1.540004000

H	-6.791317000	0.123391000	-1.192542000
H	-5.543152000	1.300363000	-1.678609000
H	-5.671170000	-0.266154000	-2.520180000

($(^3MC_{eq\ 1}/S_0)_{stc\text{-}mecp}$ UPBE0/DEF2-SVP CPCM (CH_3CN).

Ir	0.978641000	-0.131384000	0.029492000
N	-1.761149000	-0.119457000	-1.974487000
N	2.129809000	0.371891000	-1.591432000
N	-0.166553000	-0.664005000	1.651927000
C	-1.500926000	-2.344818000	2.700740000
C	-0.715419000	-1.910504000	1.625938000
C	-0.404292000	0.160380000	2.682935000
C	-0.396465000	-2.659482000	0.412044000
C	0.382993000	-1.973789000	-0.564289000
C	0.760284000	-2.627855000	-1.743593000
C	0.344674000	-3.934566000	-1.956605000
C	-0.432729000	-4.630435000	-1.036372000
C	-0.787709000	-3.975613000	0.133943000
C	3.444380000	0.635600000	-1.357641000
C	4.277093000	0.995830000	-2.423384000
C	2.392147000	0.852000000	-3.912732000
C	1.613268000	0.490149000	-2.823320000
C	3.816795000	0.524456000	0.054372000
C	2.753379000	0.261158000	0.966334000
C	3.023318000	0.129860000	2.333445000
C	4.329260000	0.272709000	2.785359000
C	5.389857000	0.535478000	1.926094000
C	5.112047000	0.654590000	0.571328000
H	-0.754099000	-5.656564000	-1.218832000
F	-1.537293000	-4.657634000	1.003723000
H	1.362746000	-2.142732000	-2.513553000
F	0.689696000	-4.550058000	-3.086434000
H	2.241649000	-0.082661000	3.065707000
F	6.144845000	0.898712000	-0.237641000
F	4.583615000	0.155534000	4.087072000
H	6.410983000	0.640197000	2.294992000
H	0.062003000	1.146994000	2.632274000
C	-1.738256000	-1.492140000	3.772000000
H	-1.922528000	-3.346990000	2.690148000
H	-2.352861000	-1.828658000	4.610052000
C	-1.189648000	-0.210835000	3.763320000
H	-1.354214000	0.493593000	4.579865000
C	3.747803000	1.099906000	-3.704989000
H	0.540591000	0.285941000	-2.906730000
H	1.936324000	0.934818000	-4.900393000
H	4.394888000	1.382878000	-4.538472000
H	5.329301000	1.201510000	-2.241280000
C	-0.204543000	4.083835000	0.061668000
C	-2.226569000	0.491051000	-0.883803000
C	-3.338472000	0.041454000	-0.164149000
C	-4.020324000	-1.106640000	-0.582555000
C	-3.530522000	-1.724537000	-1.742689000
C	-2.413801000	-1.204262000	-2.389335000
H	-3.646676000	0.591874000	0.725142000
H	-4.008080000	-2.617530000	-2.151565000
C	-1.527010000	1.745158000	-0.486673000
C	-2.234401000	2.941457000	-0.410868000
C	-1.587659000	4.153923000	-0.133742000
C	0.442359000	2.857817000	-0.005145000
N	-0.198537000	1.709699000	-0.261964000
H	-3.308202000	2.911548000	-0.604969000
H	0.393661000	4.969536000	0.273512000
H	1.519405000	2.788307000	0.168806000
H	-2.027315000	-1.693350000	-3.290073000
C	-2.378307000	5.456990000	-0.080383000
C	-1.488002000	6.649757000	0.271055000
H	-1.008152000	6.526096000	1.254183000
H	-4.060108000	6.260750000	1.032707000
C	-3.479513000	5.326443000	0.984383000
H	-3.046631000	5.140526000	1.979632000
H	-4.178675000	4.508910000	0.753643000

H	-2.101048000	7.562558000	0.310610000
H	-0.700378000	6.809885000	-0.481274000
C	-3.016205000	5.700368000	-1.458925000
H	-3.587135000	6.641798000	-1.440989000
H	-2.246641000	5.781625000	-2.242246000
H	-3.708298000	4.892081000	-1.739162000
C	-5.234003000	-1.672692000	0.153120000
C	-5.515665000	-0.921747000	1.455871000
H	-4.662788000	-0.975004000	2.150513000
H	-5.837415000	-3.560081000	1.044224000
C	-4.983102000	-3.153425000	0.480772000
H	-4.076347000	-3.278906000	1.092241000
H	-4.864157000	-3.760539000	-0.428980000
H	-6.384873000	-1.373901000	1.956965000
H	-5.750515000	0.138961000	1.277800000
C	-6.461274000	-1.552417000	-0.765597000
H	-7.347839000	-1.969407000	-0.262096000
H	-6.670243000	-0.499305000	-1.010392000
H	-6.317333000	-2.102008000	-1.708148000

Bibliography

1 I. D. Dergachev, V. D. Dergachev, M. Rooein, A. Mirzanejad and S. A. Varganov, *Acc Chem Res*, 2023, **56**, 856–866.

2 M. and D. I. D. and L. A. O. and M. R. C. and V. S. A. Dergachev Vsevolod D. and Rooein, in *New Horizons in Computational Chemistry Software*, ed. C. H. and O. M. Filatov Michael and Choi, Springer International Publishing, Cham, 2022, pp. 79–103.

3 M. Martínez-Alonso, J. Cerdá, C. Momblona, A. Pertegás, J. M. Junquera-Hernández, A. Heras, A. M. Rodríguez, G. Espino, H. Bolink and E. Ortí, *Inorg Chem*, 2017, **56**, 10298–10310.

4 D. Tordera, M. Delgado, E. Ortí, H. J. Bolink, J. Frey, M. K. Nazeeruddin and E. Baranoff, *Chemistry of Materials*, 2012, **24**, 1896–1903.

5 I. Soriano-Díaz, E. Ortí and A. Giussani, *Dalton Transactions*, 2023, **52**, 10437–10447.

6 I. Soriano-Díaz, E. Ortí and A. Giussani, *Inorg Chem*, 2021, **60**, 13222–13232.

7 Y. Luo, L. Tang, Z. Chen, Z. Xu, J. Hu and D. Tang, *New Journal of Chemistry*, DOI:10.1039/d3nj01034a.

8 Y. Luo, Z. Chen, Z. Xu and D. Tang, *New Journal of Chemistry*, 2023, **47**, 3793–3801.

9 L. Tang, J. Gao, Y. Luo, Y. Cheng, L. Liu, D. Zheng, L. Liang, J. Hu and T. Luo, *New Journal of Chemistry*, 2023, **47**, 15076–15088.
